# Supplementary material for: Within-population variability in a moth sex pheromone blend, part 2: selection towards fixation
Source: R Soc Open Sci. 2019 Mar 13;6(3):182050. doi: 10.1098/rsos.182050 (PMC6458377; doi:10.1098/rsos.182050)
Supplement: Suppl Material 291118 [file rsos182050supp1.doc]

**Supplementary Material to**

Groot AT, van Wijk M, Villacis-Perez E, Kuperus P, Schöfl G, van Veldhuizen D, Heckel D. Within-population variability in a moth sex pheromone blend, part 2: Selection towards fixation. Royal Society Open Science.

a. Selection for ‘High’ and ‘Low’ pheromone

b. Heritability analysis

c. Phenotypic analysis

d. Genetic analysis

e. Comparison between 2007 and 2012

f. Assessing pleiotropic effects on male response

g. Assessment of pleiotropic effects on larval development and adult fertility

**Supplementary Tables**

-Table S1: Single pair matings (SPM) set up for the selection lines in 2012

-Table S2: Type of crosses conducted in 2012.

-Table S3: AFLP primer combinations used and number of markers per primer combination

-Table S4: Homologizing chromosomes between backcross families

-Table S5: Primer combinations used to map allelic variation delta-9-desaturase (KPSE) and delta-11-desaturase (LPAQ)onto the genetic map, and to sequence delta-11-desaturase.

**Supplementary Figures**

-Figure S1. Phenotype of all hybrid crosses and backcrosses

-Figure S2: Significant LOD scores for individual pheromone components

-Figure S3: Schematic overview of delta-11-desaturase gene structure with all primers used.

-Figure S4. Comparison between the QTL found in 2007 and 2012.

-Figure S5: Pheromone composition in backcross females homozygous HH, heterozygous HL or homozygous LL for C04 and C21.

-Figure S6: Correlation between the presence of the High band (large intron2) and the ratio 16:Ald/Z11-16:Ald in the field collected females (plate 1-4)

-Figure S7: Growth rate and fertility of the selection lines

-Figure S8: Fit of q, the frequency of the stop-codon allele, to a one-locus selection model.

**Supplementary files**

-Suppl. File 1: Numbers of single pair matings and females phenotyped per generation in the selection lines of 2007 and of those in 2012.

-Suppl. File 2: Gels showing the screens of the stop codon

-Suppl. File 3: Aligned nucleotide sequences of the entire LPAQ delta-11-desaturase gene (gDNA) of 47 individuals. “L allel cDNA” refers to the consensus sequence of the cds allele in the High selection line, “L allel gDNA” to the consensus genomic DNA sequence in the High selection line (the names “High” and “Low” are reversed in our rearing. To stay consistent with our previous publication, our reference to “High” and “Low” in this manuscript is the same as in Groot et al. 2014). H56 refers to plate H56 of DGH, containing individuals from the JEN2 rearing of Jena. Individuals with numbers starting with 9, 10, 13, 14, 15, 18 refer to individuals from our selection lines, where the first number is the rearing generation (i.e. 9 refers to the 9^th^ generation of rearing, which is the 5^th^ generation of selection). Individuals with numbers starting with 35 or 46 are from backcross families 35 and 46, respectively.

-Suppl. File 4: Aligned nucleotide sequences of the exons of LPAQ delta-11-desaturase of field-collected females: ‘nc’: North Carolina, ‘ms’: Mississippi, ‘mxe’: East coast Mexico (near Tampico), ‘tx’: Texas (see Groot et al. 2014 on specific locations of these field collections). “L allel cDNA” refers to the consensus sequence of the cds allele in the High selection line, “L allel gDNA” to the consensus genomic DNA sequence in the High selection line.

-Suppl. File 5: Aligned nucleotide sequences of the second intron after the start codon of LPAQ delta-11-desaturase of field-collected females. “L allel cDNA” refers to the consensus sequence of the cds allele in the High selection line, “L allel gDNA” to the consensus genomic DNA sequence in the High selection line. “H-allel fam 35-07 gDNA” refers to the genomic DNA sequence of the Low allele in individual 7 of backcross family 35.

-Suppl. File 6: Gels showing the intron-size polymorphism of the second intron of LPAQ delta-11-desaturase.

-Suppl. File 7: Detailed information on “Plate 1-4”, which contain field-collected females from different geographic locations, collected in 2005-2008 as larvae, after which pheromone glands were analysed in 2-5 day-old virgin females (see Groot et al. 2014 for more information).

-Suppl. File 8: Fertility of the selection lines

***a. Selection for ‘High’ and ‘Low’ pheromone***

Single pair matings were randomly grouped into three groups, a ‘baseline’ in which no selection took place, the ‘High’ line, to select for females with a high ratio of 16:Ald / Z11-16:Ald (the major sex pheromone component) and the ‘Low’ line, to select for a low ratio. The next generation, after a new round of single pair matings, the pheromone glands of the females used in the single pair matings were extracted and analysed, after which two groups of families with the highest ratio were chosen for the next generation of the High line, two groups of families with the lowest ratio for the Low line (see Table S1 for the numbers of families per line per generation). The females in the baseline were partly analysed, but there was no selection of specific families for the next generation. The pheromone glands of females were extracted after the females had been in a mating cup for one week to mate and oviposit eggs. To ensure pheromone production, females were injected with PBAN, as before (see [1]).

***b. Heritability analysis***

Heritability of pheromone composition was calculated using an animal-model framework, in the same way as described in[1] and summarized here. The heritability was estimated based on response to selection (realized heritability), as well as estimated from the resemblance between relatives. The cross-generational relationship matrix fully accounts for effects of non-random mating, selection-induced changes in gametic-phase disequilibrium and allele-frequency changes by drift over the generations [2-4].

Prior distributions for random effects were based on an inverted Wishart distribution [4]. All MCMCglmms were run for 500,000 iterations with a burn-in period of 100,000 interations and a thinning interval of 40. Realized heritability was estimated based on weighted least-square regression on the cumulative response and cumulative selection differential over 12 generations. Since the expected response to selection is zero if there is no selection, the regression line was force to pass through the origin. Data point sizes were scaled by number of individuals measured with each generation. Additive genetic and environmental variance components for the pheromone data were estimated separately for the High and Low lin,e using an animal model based on the Bayesian implementation of generalised linear mixed-effects models provided by the R package MCMCglmm [5]. Both animal and shared environmental (i.e. generational) effects were included as random effects in the final model. The relationship matrix was constructed from pedigrees back to the foundational families of the High and the Low line, respectively.

***c. Phenotypic analysis***

To determine a possible maternal effect or sex linkage, we first measured the pheromone composition of hybrid offspring females from H x L (n=50) and L x H crosses (n=47) (female parent always written first). Of each of the 10 backcross families, we phenotyped 14-74 females per family (see Table S2). Analysis of hybrid females showed that the Low phenotype is almost completely dominant (Figure S1).

***d. Genetic analysis***

The HL male and female parents were siblings and the H male and female parents were siblings, which facilitated finding AFLP markers that occurred in both families to homologize the linkage groups. For the QTL analysis, female-informative BC46 and male-informative BC35 were genotyped, using 174 and 111 AFLP markers from 44 and 22 primer combinations respectively (see Table S3). A linkage map was constructed using the female-informative backcross (BC46) to identify the 30 autosomes, as no crossing-over occurs in female Lepidoptera (Heckel 1993) and linkage groups can thus be considered chromosomes. The chromosomes in the female-informative cross contained at least two markers from two different primer combinations, and maximally 14 (average number of markers was 6 (5.9). As the female and male parents of these families were siblings, we were able to homologize the linkage groups between the two families: we found the same markers in the male-informative cross for 23 of the 30 chromosomes (Table S4).

After constructing the linkage maps, we mapped our main candidate gene LPAQ delta-11-desaturase, as well as the KPSE delta-9-desaturase that had mapped to the QTL in our previous cross (Groot et al. 2014). As LPAQ delta-11-desaturase mapped to QTL chromosome 21, this QTL is homologous to *Bombyx mori* chromosome 23, while KPSE delta-9-desaturase mapped to chromosome 4, which is homologous to *Bombyx mori* chromosome 12 (see [1]).

A two step-method of QTL analysis was chosen, following Belknap [6] and [7]: first, a preliminary QTL analysis was done with the female-informative backcross (family 46), on the ratio of 16:Ald / Z11-16:Ald. To confirm the QTL found in the first step, a second QTL analysis was done with the male-informative backcross, in which a fine-scale linkage map was constructed with markers common to the female- and male-informative backcross families to identify the 95% Bayes credible interval of the QTL location. Genetic map construction and QTL analysis was conducted in R (version R-3.1.2), using the package R/qtl and followed methods described in [8]. For the QTL analysis, we used multiple imputation, which is essentially a Bayesian method with the log posterior distribution (LPD) of the QTL location, which is similar to the LOD scores from standard interval mapping methods. The significance threshold for the LOD score was empirically obtained by 5000 permutations (Figure S2).

The explained phenotypic variance in the backcrosses to the High line can be estimated from the LOD score obtained for a QTL as 1-10^(2/n)*LOD))^ [8] and was 89% for BC46, 99% for BC35 and 68% in BC23. The QTL on chromosome 21 not only explained most of the selected variation in the 16:Ald / Z11-16:Ald ratio between the lines, it also explained variation in all other pheromone compounds, except Z9-14:Ald, in both BC46 and BC35 (Figure S2). Using multiple imputation, the LOD score for another QTL explaining variance in Z9-16:Ald was detected on chromosome 15 of BC46 (P = 0.052) and on chromosome 20 for BC35 (P = 0.029). In BC23, the allelic variation in delta-11-desaturase yielded significant LOD scores for 14:Ald (P < 0.0001), Z11-16:Ald (P < 0.0001), 16:Ald (P < 0.0001), but not for Z11-16:OH (P = 0.064), Z9-14:Ald (P = 0.742), Z7-16:Ald (P = 0.993) and Z9-16:Ald (P = 0.962).

To further differentiate alleles of the delta-11-desaturase gene, we sequenced the complete gene from start to stop codon, which included two introns (see Figure S3). In addition to the stop codon, we found an intron size polymorphism in delta-11-desaturase that occurred not only in the selection lines, but also in the JEN2 starting population and in field collected moths (see Suppl. File 6). This allelic variation was significantly correlated to the ratio of 16:Ald / Z11-16:Ald, as shown in Figure S6.

***e. Comparison to previous analysis***

We explained our previous finding that the HL heterozygote in the backcross unexpectedly produced more unsaturated compounds than the LL homozygote with a homodimer acting as a repressor: as we explained, the desaturase activity in HL females would be higher than in LL females, because its desaturase expression is not repressed in hybrids due to a mismatch of the dimer (see Figure S4 and Groot et al. 2014).

Pheromone compositions of all backcross individuals were compared to determine significant differences in the sex pheromone blend between LL, HL and HH females for chromosome 4 and chromosome 21 (Figure S5).

***f. Assessing pleiotropic effects on male response***

Male physiological EAG responses were measured as follows. Severed heads were placed on a glass reference probe, while a second glass electrode was mounted on the tip of the antenna that had its most distal segment removed. Both probes contained Ag-AgCl electrodes in a Ringer solution. Signals were initially preamplified (10x) by an AC/DC converter (Syntech IDAC4) and subsequently amplified (100x) before recording on a PC running EAG2000 software (Syntech). A constant air ﬂow (2.0 ml/s) of charcoal filtered, humidiﬁed air was provided to the preparation. Stimuli were provided through an independent glass pipette which altered the airflow 5%, stimulus duration was 1 s and the inter-stimulus interval was at least one minute to prevent adaptation. Responses to pheromone compounds were standardized using *Z*-3-hexen-1-ol as a reference stimulus. For the two major sex pheromone components (Z11-16:Ald and Z9-14:Ald) and their saturated counterparts (14:Ald and 16:Ald), a dose-response relation was measured for five concentrations (0.2, 2, 20, 200 and 20,000 ng/µl hexane). For each stimulus, 5 ul pheromone solution on filter paper was provided to the stimulus pipette. To compare EAG responses between the selection lines, linear mixed effects analysis (lme) was performed in R, using the lme4 package. The best fit of the model was obtained after arcsin square root transformation of the relative EAG response. The initial model contained the odor concentration, the time of the experiment and moth line (High or Low) as fixed effects, while moth was added to the model as a random effect. Selection of the model that best fitted the data was conducted using second-order AIC (AICc) criteria. The selected models contained moth line and concentration as covariates.

***g. Assessment of pleiotropic effects on larval development and adult fertility***

In discovering the premature stop codon in the delta-11-desaturase gene in the first coding exon, we were curious to determine whether the effective knock-out of this gene had any pleiotropic effect on larval development and adult fertility. Larval development in the Low and High lines was determined by measuring the larval growth rate and weight gain in the 4^th^ and 12^th^ generation after the start of selection. In the 4^th^ generation, the weights of third instar larvae were measured at three time points with 2 days in between, while in the 12^th^ generation with 6 days in between. The growth rate of the larvae was determined by taking the log weight of the second or third measuring day minus the log of the starting weight, divided by the number of days between the two measuring points. In addition, the mating success of single pair crosses within Low, High and backup lines was determined in the first 10 generations of selection. Every generation, we set up 15-45 single pair matings for each line and determined how many of these families produced fertile eggs.

To further explore possible pleiotropic effects of the LPAQ delta-11-desaturase knock-out through fixation by selection of the stop-codon in the first exon, we measured the growth rate of larvae from the the High and Low lines in generation 4 and 12, but found no difference in growth rate in either generation (Figure S7a). In addition, we determined whether the High and Low lines differed in the number of fertile families, which was not the case either (Figure S7b).

**References**

**Table S1**. Single pair matings (SPM) set up for the selection lines in 2012

| Generation  (=sel.gen) | Line | # SPM | # Females phenotyped | # Females w  ratio > 1 | % Females with  ratio > 1 |
| --- | --- | --- | --- | --- | --- |
| 5 (=1) | Base | 13 | 10 | 0 | - |
|  | High | 40 | 31 | 2 | 6.5 |
|  | Low | 27 | 21 | 1 | 4.8 |
| 6 (=2) | Base | 40 | 29 | 0 | - |
|  | High | 22 | 22 | 1 | 4.5 |
|  | Low | 20 | 18 | 1 | 5.5 |
| 7(=3) | Base | 21 | 21 | 0 | - |
|  | High | 70 | 70 | 7 | 10 |
|  | Low | 39 | 39 | 0 | - |
| 8(=4) | Base | 11 | 7 | 0 | - |
|  | High | 61 | 60 | 3 | 5 |
|  | Low | 35 | 35 | 2 | 5.7 |
| 9(=5) | Base | 9 | 0 | - | - |
|  | High | 47 | 47 | 8 | 17 |
|  | Low | 19 | 19 | 1 | 5.3 |
| 10(=6) | Base | 10 | 0 | - | - |
|  | High | 70 | 68 | 20 | 29.4 |
|  | Low | 47 | 47 | 0 | - |
| 11(=7) | Base | 7 | 0 | - | - |
|  | High | 76 | 73 | 28 | 38.4 |
|  | Low | 34 | 31 | 0 | - |
| 12(=8) | Base | 9 | 0 | - | - |
|  | High | 61 | 60 | 33 | 55 |
|  | Low | 37 | 30 | 0 | - |
| 13(=9) | Base | 15 | 0 | - | - |
|  | High | 52 | 53 | 37 | 72.5 |
|  | Low | 35 | 35 | 0 | - |
| 14(=10) | Base | 15 |  |  |  |
|  | High | 61 | 62 | 50 | 80.3 |
|  | Low | 23 | 24 | 1 | - |
| 15(=11) | Base | 17 | 0 | - | - |
|  | High | 58 | 58 | 53 | 91.4 |
|  | Low | 33 | 30 | 0 | - |
| 16(=12) | Base | - |  |  |  |
|  | High | 68 | 68 | 62 | 91.2 |
|  | Low | 70 | 70 | 1 | 1.4 |
| 17(=13) | Base | 27* |  |  |  |
|  | High | 124 | 56 | 54 | 97.9 |
|  | Low | 103 | 51 | 0 | - |
| 18(=14) | Base | 30 |  |  |  |
|  | High | 125 | 51 | 51 | 100 |
|  | Low | 98 | 48 | 2 | 4.2 |
| 19(=15) | Base | 25 |  |  |  |
|  | High | 97 | 29 | 28 | 96.5 |
|  | Low | 80 | 31 | 1 | 3.2 |

* Base line of generation 17 is a mix of generation 16 Low x Base generation 15 that were set cold

**Table S2**. Type of crosses conducted in 2012.


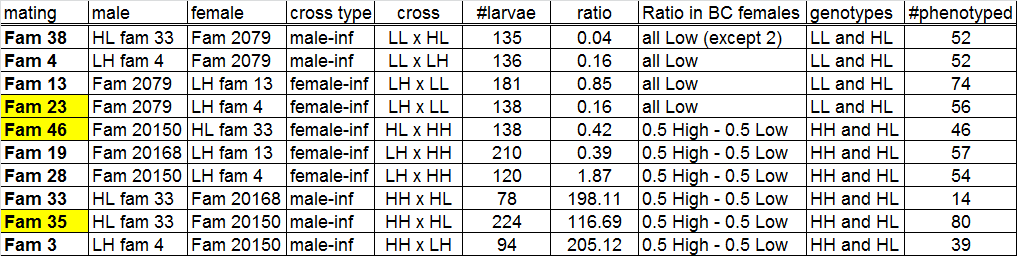


**Table S3**. AFLP primer combinations and number of markers scored per primer combination

| Mse-CNN | GATGAGTCCTGAGTAA CNN |
| --- | --- |
| Eco-ANN | GACTGCGTACCAATTC ANN |

| **Female-informative BC46** | | |  | **Male-informative BC35** | | |
| --- | --- | --- | --- | --- | --- | --- |
| Eco | Mse | # markers |  | Eco | Mse | # markers |
| AAC | CAA | 6 |  | AAC | CAC | 4 |
| AAC | CAC | 5 |  | AAC | CAG | 6 |
| AAC | CAG | 4 |  | AAC | CAT | 6 |
| AAC | CAT | 6 |  | AAC | CGT | 6 |
| AAC | CCT | 3 |  | ACA | CAC | 3 |
| AAC | CGT | 5 |  | ACA | CAG | 2 |
| ACA | CAC | 1 |  | ACA | CAT | 1 |
| ACA | CAG | 1 |  | ACA | CCA | 6 |
| ACA | CAT | 4 |  | ACA | CGA | 5 |
| ACA | CCA | 4 |  | ACA | CGT | 8 |
| ACA | CGA | 4 |  | ACT | CAA | 9 |
| ACA | CGT | 3 |  | ACT | CCA | 3 |
| ACA | CTA | 2 |  | ACT | CTA | 5 |
| ACT | CAA | 8 |  | AGA | CCT | 8 |
| ACT | CCA | 2 |  | AGA | CGA | 4 |
| ACT | CTA | 9 |  | AGT | CAC | 7 |
| ACT | CTT | 7 |  | AGT | CCA | 5 |
| AGA | CAC | 1 |  | ATC | CCT | 9 |
| AGA | CAT | 4 |  | ATG | CAA | 7 |
| AGA | CCT | 4 |  | ATG | CAC | 1 |
| AGA | CGA | 2 |  | ATG | CCA | 5 |
| AGA | CTA | 5 |  | ATG | CTA | 1 |
| AGA | CTC | 3 |  |  | | |
| AGA | CTG | 8 |  |  | | |
| AGT | CAC | 8 |  |  | | |
| AGT | CCA | 6 |  |  | | |
| AGT | CCT | 6 |  |  | | |
| AGT | CGA | 6 |  |  | | |
| AGT | CTC | 2 |  |  | | |
| ATC | CAA | 1 |  |  | | |
| ATC | CAG | 1 |  |  | | |
| ATC | CCA | 1 |  |  | | |
| ATC | CCT | 9 |  |  | | |
| ATC | CTC | 2 |  |  | | |
| ATC | CTT | 2 |  |  | | |
| ATG | CAA | 4 |  |  | | |
| ATG | CAC | 4 |  |  | | |
| ATG | CAT | 2 |  |  | | |
| ATG | CCA | 4 |  |  | | |
| ATG | CCT | 3 |  |  | | |
| ATG | CGA | 1 |  |  | | |
| ATG | CTA | 3 |  |  | | |
| ATG | CTC | 3 |  |  | | |
| ATG | CTG | 5 |  |  | | |

**Table S4.** **Homologizing chromosomes between backcross families.** “Nr. of overlapping AFLP markers” is number of AFLP markers scored in female-informative backcross family 46 that were found in male-informative backcross family 35.”Unlinked” is an artificial linkage group of unlinked markers in family 46 and “not homologized” is an artificial linkage group of linkage groups in family 35 that could not be homologized using overlapping markers with a chromosome from family 46.

| **Family 46 (fem. inf.)** | | **Family 35 (male inf.)** | |  |  |
| --- | --- | --- | --- | --- | --- |
| Chromosome nr. | Nr. of AFLP markers | Chromosome  nr. | Nr. of AFLP Markers | Nr. of overlapping AFLP markers | Mapped gene |
| **1** | 2 |  |  |  |  |
| **2** | 14 |  |  |  |  |
| **3** | 13 | **3** | 8 | 5 |  |
| **4** | 8 | **4** | 5 | 5 | **KPSE** |
| **5** | 11 | **5** | 6 | 5 |  |
| **6** | 10 | **6** | 3 | 2 |  |
| **7** | 11 | **7** | 3 | 2 |  |
| **8** | 7 | **8** | 1 | 1 |  |
| **9** | 6 | **9** | 2 | 2 |  |
| **10** | 6 | **10** | 8 | 3 |  |
| **11** | 6 |  | 0 |  |  |
| **12** | 5 | **12** | 3 | 2 |  |
| **13** | 5 | **13** | 1 | 1 |  |
| **14** | 5 | **14** | 1 | 1 |  |
| **15** | 5 |  |  |  |  |
| **16** | 5 | **16** | 1 | 1 |  |
| **17** | 5 | **17** | 1 | 1 |  |
| **18** | 5 | **18** | 7 | 5 |  |
| **19** | 5 | **19** | 5 | 3 |  |
| **20** | 4 | **20** | 2 | 1 |  |
| **21** | 14 | **21** | 10 | 10 | **d-11-desat** |
| **22** | 3 |  |  |  |  |
| **23** | 3 | **23** | 3 | 2 |  |
| **24** | 3 |  |  |  |  |
| **25** | 3 | **25** | 5 | 3 |  |
| **26** | 3 | **26** | 3 | 2 |  |
| **27** | 4 | **27** | 8 | 4 | **HR14** |
| **28** | 2 | **28** | 1 | 1 |  |
| **29** | 2 |  |  |  |  |
| **unlinked** | 2 | **Not homologized** | 27 | 1 |  |
|  |  |  |  |  |  |

**Table S5**. Primer combinations used to map allelic variation delta-9-desaturase (KPSE) and delta-11-desaturase (LPAQ)onto the genetic map, and to sequence delta-11-desaturase (see Figure S3 for a schematic overview of the primer locations). We designed primers such that we could distinguish heterozygous from homozygous backcross individuals in the PCR products, i.e. for LPAQ we designed a primer on top of the stop codon that we found at bp 40 of the cds, and for KPSE we designed primers that yielded one band in homozygous LL and HH females and two bands in heterozygous HL females in families 46, 35 and 23.

| **Primer overview** | **Primer lab name** | **Sequence** |
| --- | --- | --- |
| D11-58F | D11 PK H+L 58 F | GCGCAAAGCTATCAATCAACTAC |
| D11-nonstopF | D11 PK H-nonstop 94 F | ATCAACTACAGTTTTGAGTGAGG |
| D11-nonstopF | D11 new nonstop F | AACTACAGTTTTGAGTGAGG |
| D11-stopF | D11 PK L-stop 94 F | CAATCAACTACAATTTTGAGTGAGtA |
| D11-stopF | D11 new stop F | CAACTACAATTTTGAGTGAGT |
| D11-247R | D11 H+L 247- R | AAATTGTAGCCCATTTAGCAGA |
| D11-247R | D11 new 247 R | ATTGTAGCCCATTTAGCAG |
| D11-294R | D11 PK 294 R | AGCCGTGATCCCTATTTCTG |
| D11-2F | D11 exon 2 F | GCAGAAATAGGGATCACGGC |
| D11-2R | D11 exon 2 R | AAACCGCAGCACTGGATTG |
| D11-3F | D11 exon 3 F | GGAGCTGTTTGTTTCGTCTT |
| D11-3R | D11 exon 3 R | CGTTTACGCACCACTTCACT |
| D11-634F | D11-DN-634F | CGTGGGAAAGAACTCAATATGT |
| D11-891F | D11 AH 891F | GGACCATCGTCTCCATCATAAG |
| D11-1089R | D11-AH-1089R | GCCATCTCCAGTGCGTTTAG |
|  |  |  |
| KPSE-F | KPSE exon 1 F2 | AGCAACTGGTGTGGAGGAAC |
| KPSE-R | KPSE R1 | GGATGAATGCGTAACGGAAC |

**Supplementary Figures**

**Figure S1**. **Phenotype of all hybrid crosses and backcrosses** (families 35 and 46 also genotyped, and family 23 genotyped for KPSE and D11_LPAQ). Phenotype is the ratio of 16:Ald/Z11-16:Ald in the female sex pheromone gland. Females with a ratio < 1 were categorized as Low (L; light bars), females with a ratio > 1 were categorized as High (H, black bars).


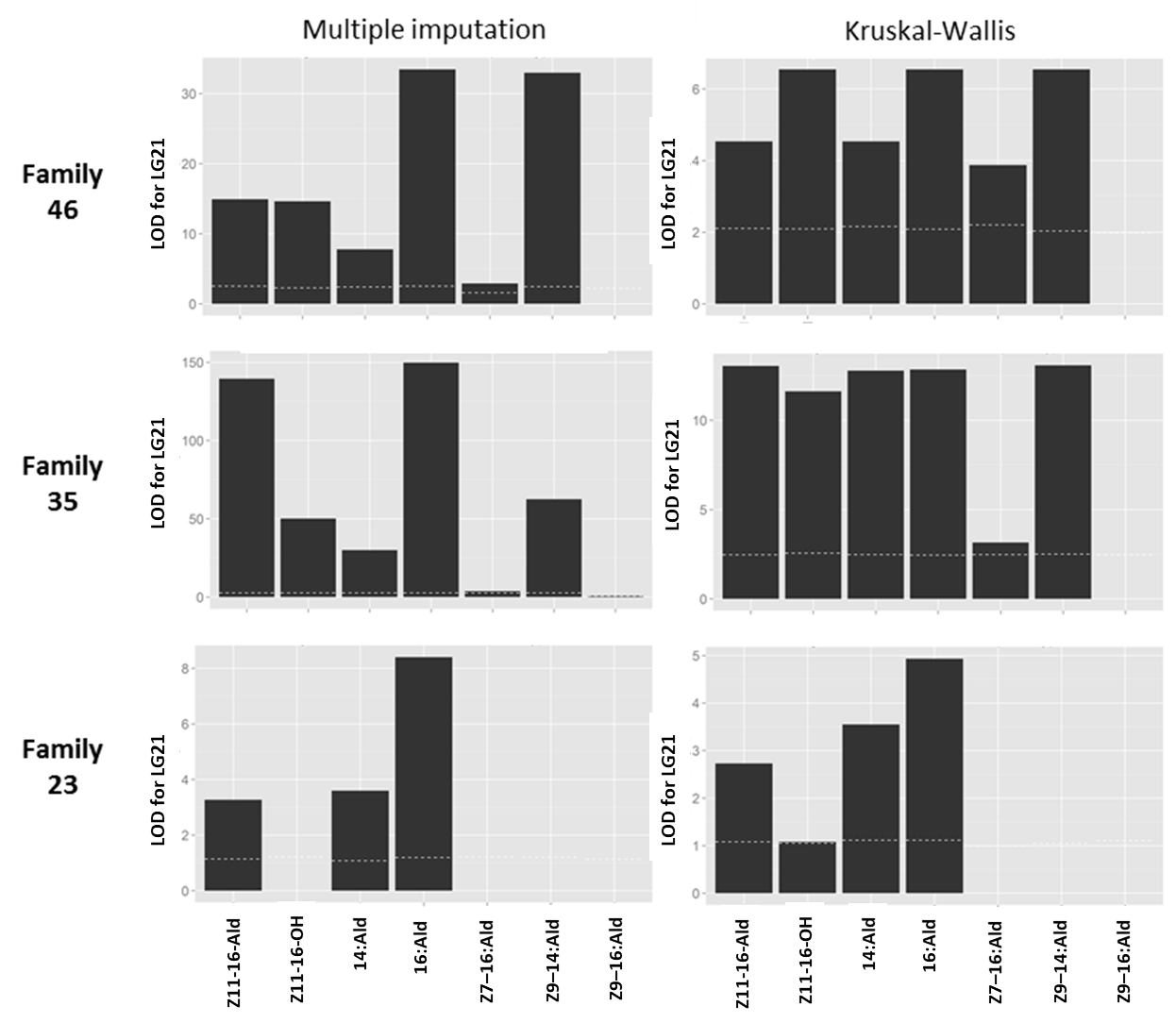


**Figure S2. Significant LOD scores for individual pheromone components**

Depicted are significant LOD sores for pheromone components in BC46, BC35 and BC23 at the QTL location on chromosome 21. LOD scores and significance levels of LOD were estimated using multiple imputation. The QTL location of all pheromone compounds is the same as the QTL we found for the selected 16:Ald / Z11-16:Ald ratio, with the exception of Z9-16:Ald. In BC23, the allelic variation in delta-11-desaturase yielded significant LOD scores for 14:Ald (P < 0.0001), Z11-16:Ald (P < 0.0001), 16:Ald (P < 0.0001), but not for Z11-16:OH (P = 0.064), Z9-14:Ald (P = 0.742), Z7-16:Ald (P = 0.993) and Z9-16:Ald (P = 0.962).

**Figure S3.** Schematic overview of LPAQ delta-11-desaturase from start to stop codon, with all primers used. Primer sequences are given in Table S5.

**Figure S4**. Comparison between the QTL found in 2007 (Groot et al. 2014) and 2012 (this study). In 2007 we mapped a trans-acting repressor of delta-11-desaturase, where we hypothesized that repression of delta-11-desaturase induces increase in Z9-14:Ald. In 2012 we mapped allelic differentiation of delta-11-desaturase, with a stop codon and thus a non-functional H allele, so that homozygous HH females have almost zero unsaturated compounds, and a functional L allele.

**Figure S5**. Pheromone composition in backcross females homozygous HH, heterozygous HL or homozygous LL for chr.4 (A, B, C) in the female-informative backcross 6Y-R (of 2007), BC23 and BC46 (of 2012) and chr.21 (D, E, F) in BC46, BC35 and BC23, respectively. Asterisks above bars indicate significant differences between homo- and heterozygous females.


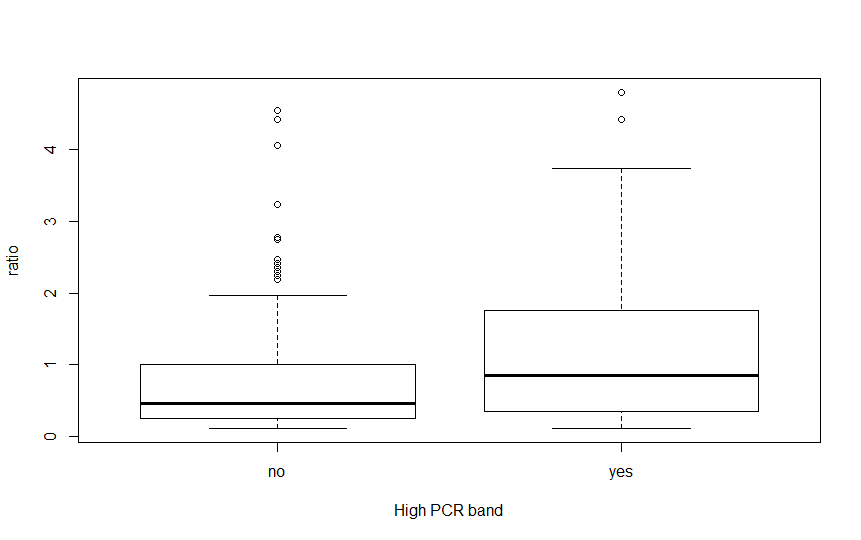


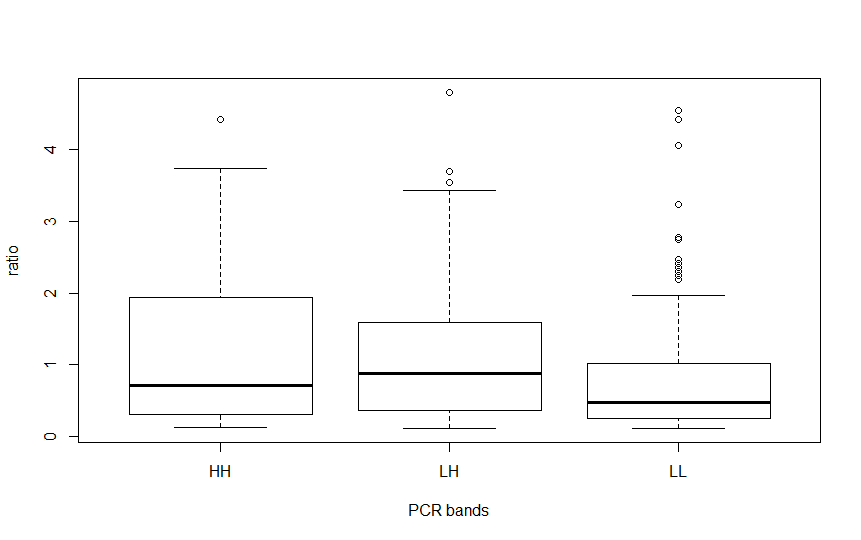


**Figure S6**. Correlation between the presence of the High band (large intron2) and the ratio 16:Ald/Z11-16:Ald in the field collected females (plate 1-4). Left graph: Comparison between females that did (yes) or did not (no) have the high PCR band (F value: 8.99, *P* = 0.0029). Right graph: Comparsion between females that only showed the high PCR band (HH), both the high and the low band (LH) or only the low band (LL) (F = 4.23, *P* = 0.0155).

**A**

**B**

**Figure S7.** Growth rate and fertility of the selection lines. Growth rate was calculated by measuring larval weight at three different days. Growth rate1 was calculated by subtracting the log weight of second weighed day from that of the first weighed day and dividing this by the number of days between the two measured points. Growth rate2 was calculated in the same way, but now subtracting the log weight of the third weighed day from that of the first weighed day. No significant differences were found between any of the growth rates (Growth rate1 generation 9: F = 0.629, P = 0.43; Growth rate2 generation 9: F = 0.093, P = 0.76; Growth rate1 generation 17: F = 2.098, P = 0.15; Growth rate2 generation 17: F = 0.008, P = 0.93. % Fertility refers to the number of single pair matings that contained fertile eggs divided by the total number of single pair matings set up (see Suppl. File 8 for details).


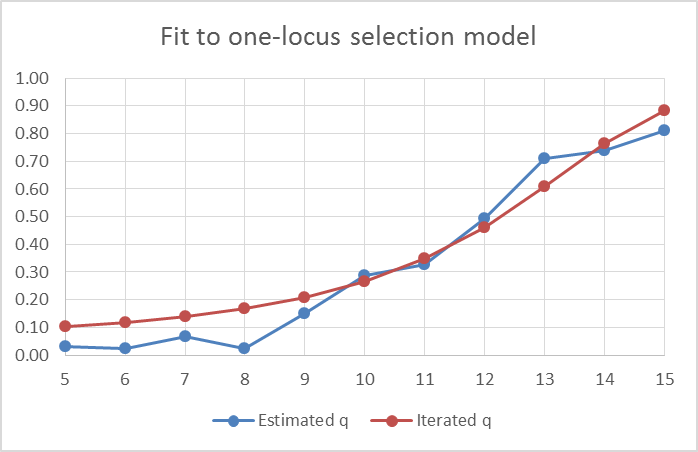


**Figure S8. Fit of q, the frequency of the stop-codon allele, to a one-locus selection model.** The model assumes relative fitnesses (1, 1, 2.76) for wild-type homozygotes, heterozygotes, and stop-allele homozygotes respectively, and a starting allele frequency of 0.01. Genotypes were assigned based on the assumption that wild-type homozygotes have a 16:Ald/Z11-16:Ald ratio of 0.04 - 0.99, heterozygotes have a ratio of 1.03 - 155.27, and stop-allele homozygotes have a ratio of 156.47 - 814.09. The iterated q follows from the model predictions, and the estimated q is based on the ratio of females that were phenotyped each generation starting with generation 5. Poor fit for the first four generations may be because it was not possible to determine the phenotypes of males.
