# Supplementary material for: Within-population variability in a moth sex pheromone blend, part 2: selection towards fixation
Source: R Soc Open Sci. 2019 Mar 13;6(3):182050. doi: 10.1098/rsos.182050 (PMC6458377; doi:10.1098/rsos.182050)
Supplement: Suppl. File 2 [file rsos182050supp3.docx]

Supplementary file 2 to Groot AT, van Wijk M, Villacis-Perez E, Kuperus P, Schöfl G, van Veldhuizen D, Heckel D. Within-population variability in a moth sex pheromone blend, part 2: Selection towards fixation. Royal Society Open Science.

Screening wild-caught *Heliothis virescens* individuals for the presence of the stop codon in the first exon on delta-11-desaturase


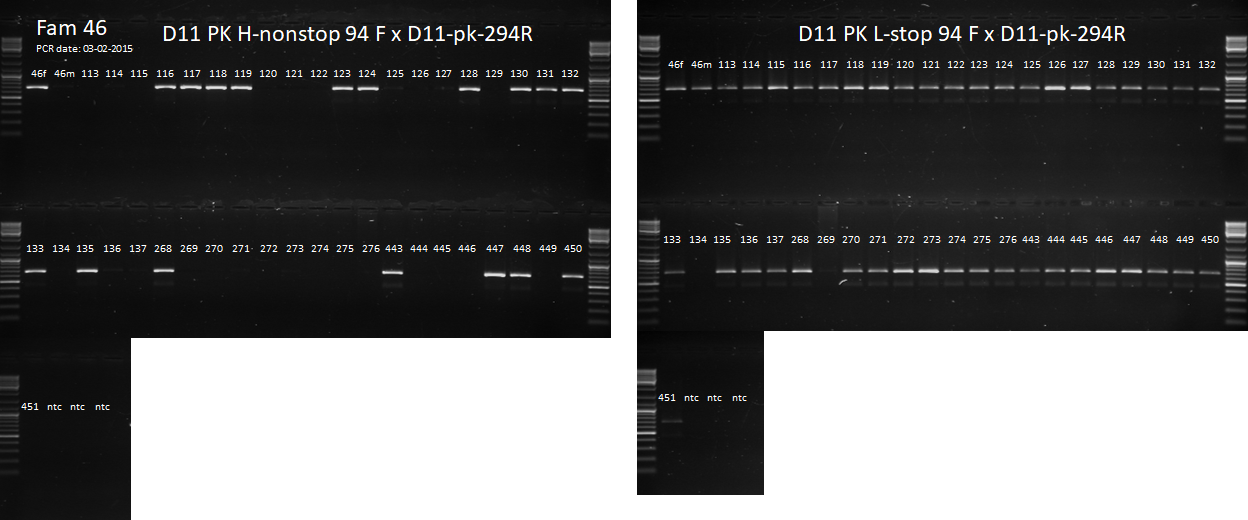


**Figure S2a**. Gels showing bands generated by a forward primer on the non-stop (left gel) or stop codon (right gel) of delta-11-desaturase in the female-informative BC46 (HL x H). The numbers above the slots are the numbers of the females.


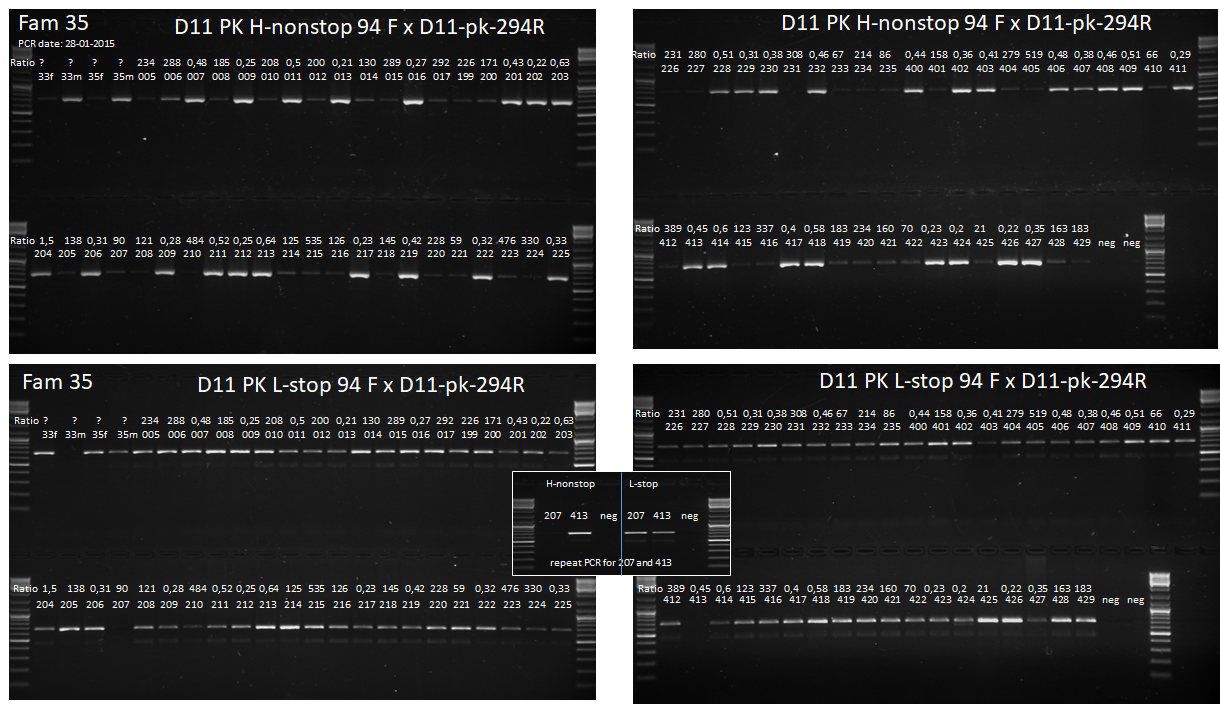


**Figure S2b**. Gels showing bands generated by a forward primer on the non-stop (upper gels) or stop codon (lower gels) of delta-11-desaturase in the male-informative BC35 (H x HL). The numbers above the slots are the ratios of 16:Ald/Z11-16:Ald and the numbers of the females.


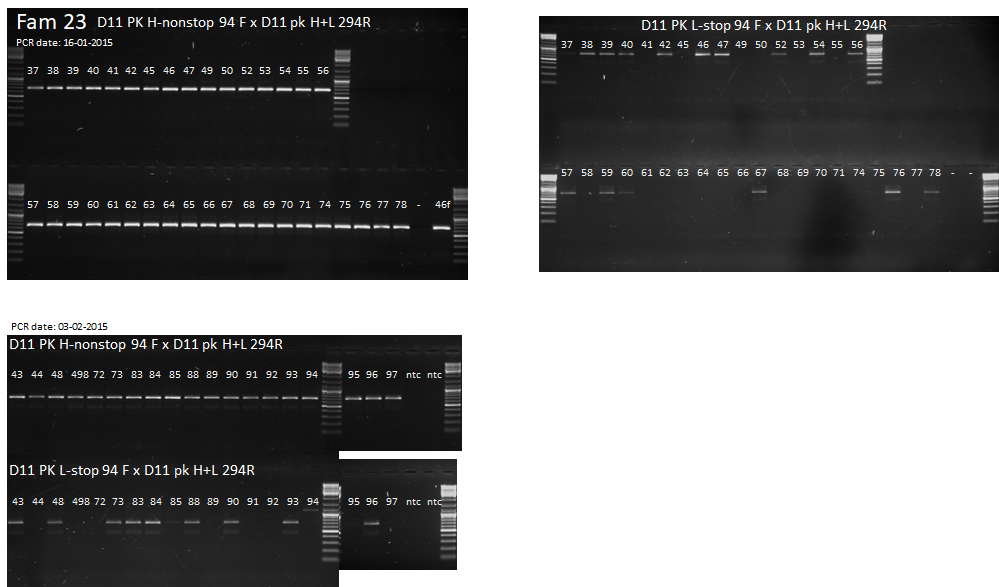


**Figure S2c**. Gels showing bands generated by a forward primer on the stop or non-stop codon of delta-11-desaturase in the female-informative BC23 (HL x L). The numbers above the slots are the numbers of the females.


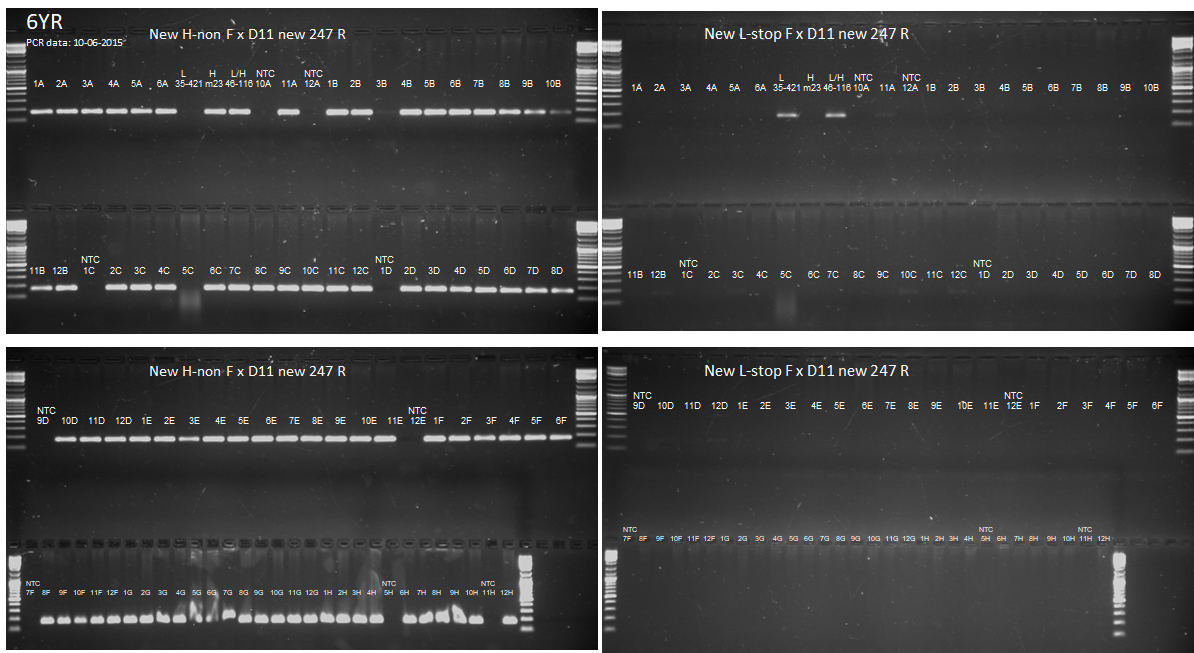


**Figure S2d**. Gels showing bands generated by a forward primer on the non-stop (left gels) or stop codon (right gels) of delta-11-desaturase in the previous female-informative backcross 6Y-R (HL x L). The numbers above the slots are the numbers of the females.


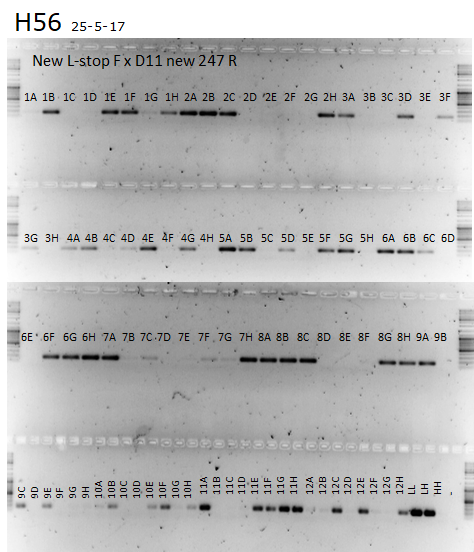


**Figure S2e**. Gels showing bands generated by a forward primer on the stop codon of delta-11-desaturase in *H. virescens* individuals from the Jen2 laboratory population of the Max Planck Institute for Chemical Ecology. The numbers above the slots are the plate slots from which DNA was extracted.


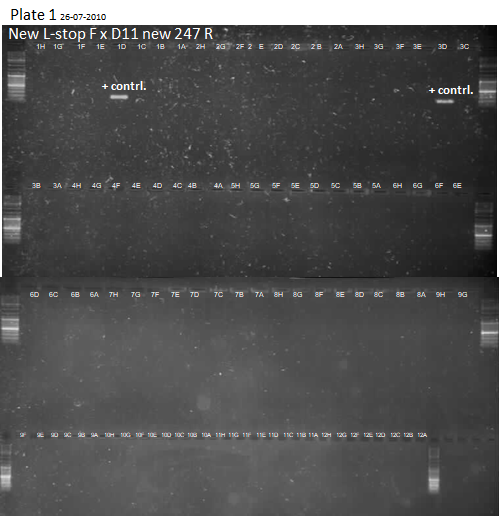

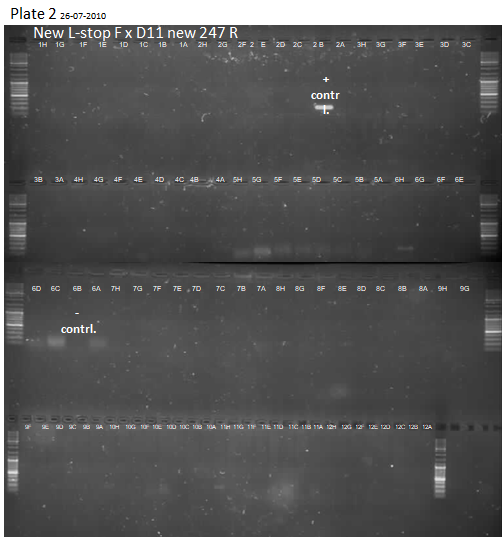


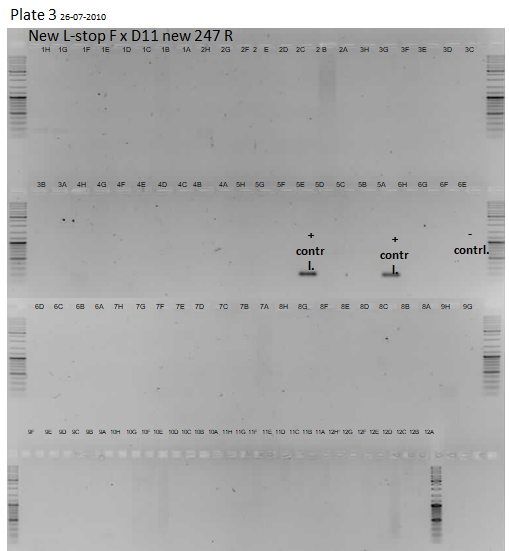

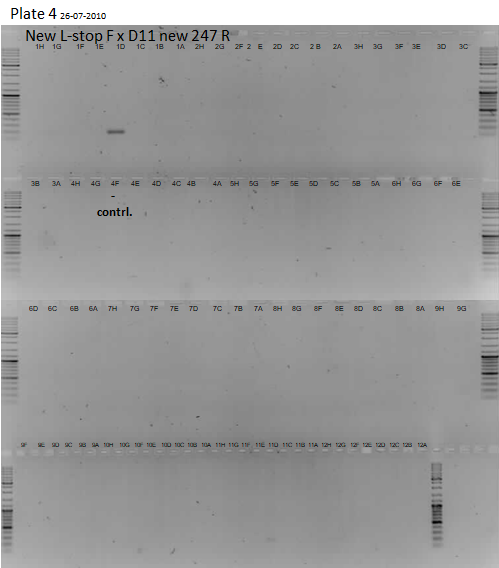


**Figure S2f**. Gels showing bands generated by a forward primer on the stop codon of delta-11-desaturase in the individuals of the four 96-well plates that were collected from the field in 2005 – 2008. The numbers above the slots are the plate slots from which DNA was extracted. Bands sequenced: plate 2 H6 + H11, plate 3 H1 + G1 + H2 + H10, plate 4 D1 + D11. The stopcodon was not found in the sequence of these samples, hence these were false positives.
