# Supplementary material for: Within-population variability in a moth sex pheromone blend, part 2: selection towards fixation
Source: R Soc Open Sci. 2019 Mar 13;6(3):182050. doi: 10.1098/rsos.182050 (PMC6458377; doi:10.1098/rsos.182050)
Supplement: Suppl. File 3 [file rsos182050supp4.docx]

Supplementary File 3 to Groot AT, van Wijk M, Villacis-Perez E, Kuperus P, Schöfl G, van Veldhuizen D, Heckel D. Within-population variability in a moth sex pheromone blend, part 2: Selection towards fixation. Royal Society Open Science.

Aligned nucleotide sequences of the entire LPAQ delta-11-desaturase gene (gDNA) of 47 individuals of BC families 35, 46 and H56

10 20 30 40 50 60 70 80 90 100

....|....|....|....|....|....|....|....|....|....|....|....|....|....|....|....|....|....|....|....|

**L allel gDNA**  **ATGGCTCAAGGCTATCAATCAACTACAATTTTGAGTGAGTAGAAAGAACCGACGCTGACCTTAGTGGTGCCCCAAGCAGCACCAAGGAAGTACCAAATAG**

**L allel cDNA**  **ATGGCTCAAGGCTATCAATCAACTACAATTTTGAGTGAGTAGAAAGAACCGACGCTGACCTTAGTGGTGCCCCAAGCAGCACCAAGGAAGTACCAAATAG**

**H allel fam35-07 gDNA** **ATGGCGCAAAGCTATCAATCAACTACAGTTTTGAGTGAGGAGAAAGAACCAACGCTGACCCTAGTGGTGCCCCAAGCAGCACCAAGGAAGTACCAAATAG**

**H56 01A**  **-----------------ATCAACTACAATTTTGAGTGAGkAGAAAGAACCGACGCTGACCTTAGTGGTGCCCCAAGCAGCACCAAGGAAGTACCAAATAG**

**H56 01B**  **-----------------ATCAACTACAATTTTGAGTGAGkAGAAAGAACCGACGCTGACCTTAGTGGTGCCCCAAGCAGCACCAAGGAAGTACCAAATAG**

**H56 01G**  **-----------------ATCAACTACAATTTTGAGTGAGGAGAAAGAACCGACGCTGACCTTAGTGGTGCCCCAAGCAGCACCAAGGAAGTACCAAATAG**

**H56 04A**  **-----------------ATCAACTACArTTTTGAGTGAGkAGAAAGAACyrACGCTGACCyTAGTGGTGCCCCAAGCAGCACCAAGGAAGTACCAAATAG**

**H56 04C**  **-----------------ATCAACTACArTTTTGAGTGAGkAGAAAGAACyrACGCTGACCyTAGTGGTGCCCCAAGCAGCACCAAGGAAGTACCAAATAG**

**H56 04D**  **-----------------ATCAACTACAATTTTGAGTGAGkAGAAAGAACCGACGCTGACCTTAGTGGTGCCCCAAGCAGCACCAAGGAAGTACCAAATAG**

**H56 07A**  **-----------------ATCAACTACArTTTTGAGTGAGkAGAAAGAACyrACGCTGACCyTAGTGGTGCCCCAAGCAGCACCAAGGAAGTACCAAATAG**

**H56 07B**  **-----------------ATCAACTACArTTTTGAGTGAGGAGAAAGAACyrACGCTGACCyTAGTGGTGCCCCAAGCAGCACCAAGGAAGTACCAAATAG**

**H56 07C**  **-----------------ATCAACTACArTTTTGAGTGAGGAGAAAGAACyrACGCTGACCyTAGTGGTGCCCCAAGCAGCACCAAGGAAGTACCAAATAG**

**H56 07F**  **-----------------ATCAACTACAGTTTTGAGTGAGGAGAAAGAACTAACGCTGACCCTAGTGGTGCCCCAAGCAGCACCAAGGAAGTACCAAATAG**

**H56 07G**  **-----------------ATCAACTACAGTTTTGAGTGAGGAGAAAGAACTAACGCTGACCCTAGTGGTGCCCCAAGCAGCACCAAGGAAGTACCAAATAG**

**H56 10A**  **-----------------ATCAACTACArTTTTGAGTGAGGAGAAAGAACyrACGCTGACCyTAGTGGTGCCCCAAGCAGCACCAAGGAAGTACCAAATAG**

**H56 10F**  **-----------------ATCAACTACArTTTTGAGTGAGkAGAAAGAACyrACGCTGACCyTAGTGGTGCCCCAAGCAGCACCAAGGAAGTACCAAATAG**

**H56 10H**  **-----------------ATCAACTACAGTTTTGAGTGAGGAGAAAGAACTAACGCTGACCCTAGTGGTGCCCCAAGCAGCACCAAGGAAGTACCAAATAG**

**H56 12A**  **-----------------ATCAACTACArTTTTGAGTGAGGAGAAAGAACyrACGCTGACCyTAGTGGTGCCCCAAGCAGCACCAAGGAAGTACCAAATAG**

**H56 12B**  **-----------------ATCAACTACAATTTTGAGTGAGkAGAAAGAACCGACGCTGACCTTAGTGGTGCCCCAAGCAGCACCAAGGAAGTACCAAATAG**

**H56 12C**  **-----------------ATCAACTACArTTTTGAGTGAGkAGAAAGAACyrACGCTGACCyTAGTGGTGCCCCAAGCAGCACCAAGGAAGTACCAAATAG**

**H56 12D**  **-----------------ATCAACTACArTTTTGAGTGAGGAGAAAGAACyrACGCTGACCyTAGTGGTGCCCCAAGCAGCACCAAGGAAGTACCAAATAG**

**9173 gDNA**  **-----------------ATCAACTACAATTTTGAGTGAGTAGAAAGAACCGACGCTGACCTTAGTGGTGCCCCAAGCAGCACCAAGGAAGTACCAAATAG**

**9183 gDNA**  **-----------------ATCAACTACAATTTTGAGTGAGTAGAAAGAACCGACGCTGACCTTAGTGGTGCCCCAAGCAGCACCAAGGAAGTACCAAATAG**

**9185 gDNA**  **----------------------------------------------------------------------------------------------------**

**10103 gDNA**  **----------------------------------------------------------------------------------------------------**

**1082 gDNA**  **----------------------------------------------------------------------------------------------------**

**1351 gDNA**  **----------------------------------------------------------------------------------------------------**

**1364 gDNA**  **----------------------------------------------------------------------------------------------------**

**14142 gDNA**  **----------------------------------------------------------------------------------------------------**

**1434 gDNA**  **----------------------------------------------------------------------------------------------------**

**15109 gDNA**  **-----------------ATCAACTACAATTTTGAGTGAGTAGAAAGAACCGACGCTGACCTTAGTGGTGCCCCAAGCAGCACCAAGGAAGTACCAAATAG**

**15282 gDNA**  **-----------------ATCAACTACAATTTTGAGTGAGTAGAAAGAACCGACGCTGACCTTAGTGGTGCCCCAAGCAGCACCAAGGAAGTACCAAATAG**

**1560 gDNA**  **-----------------ATCAACTACAATTTTGAGTGAGTAGAAAGAACCGACGCTGACCTTAGTGGTGCCCCAAGCAGCACCAAGGAAGTACCAAATAG**

**1581 gDNA**  **-----------------ATCAACTACAATTTTGAGTGAGTAGAAAGAACCGACGCTGACCTTAGTGGTGCCCCAAGCAGCACCAAGGAAGTACCAAATAG**

**1582 gDNA**  **-----------------ATCAACTACAATTTTGAGTGAGTAGAAAGAACCGACGCTGACCTTAGTGGTGCCCCAAGCAGCACCAAGGAAGTACCAAATAG**

**2816 cDNA**  **ATGGCTCAAGGCTATCAATCAACTACAATTTTGAGTGAGTAGAAAGAACCGACGCTGACCTTAGTGGTGCCCCAAGCAGCACCAAGGAAGTACCAAATAG**

**2844 cDNA**  **ATGGCTCAAGGCTATCAATCAACTACAATTTTGAGTGAGTAGAAAGAACCGACGCTGACCTTAGTGGTGCCCCAAGCAGCACCAAGGAAGTACCAAATAG**

**2848 cDNA**  **ATGGCTCAAGGCTATCAATCAACTACAATTTTGAGTGAGTAGAAAGAACCGACGCTGACCTTAGTGGTGCCCCAAGCAGCACCAAGGAAGTACCAAATAG**

**fam35 female gDNA**  **-----------------ATCAACTACAATTTTGAGTGAGTAGAAAGAACCGACGCTGACCTTAGTGGTGCCCCAAGCAGCACCAAGGAAGTACCAAATAG**

**fam35-06 cDNA**  **ATGGCTCAAGGCTATCAATCAACTaCAATTTTGAGTGAGTAGAAAGAACCGACGCTGACCTTAGTGGTGCCCCAAGCAGCACCAAGGAAGTACCAAATAG**

**fam35-06 gDNA**  **-----------------ATCAACTACAATTTTGAGTGAGTAGAAAGAACCGACGCTGACCTTAGTGGTGCCCCAAGCAGCACCAAGGAAGTACCAAATAG**

**fam35-08 cDNA**  **ATGGCTCAAGGCTATCAATCAACTACAATTTTGAGTGAGTAGAAAGAACCGACGCTGACCTTAGTGGTGCCCCAAGCAGCACCAAGGAAGTACCAAATAG**

**fam35-08 gDNA**  **-----------------ATCAACTACAATTTTGAGTGAGTAGAAAGAACCGACGCTGACCTTAGTGGTGCCCCAAGCAGCACCAAGGAAGTACCAAATAG**

**fam35-223 cDNA**  **----------------------------------------------------------------------------------------------------**

**fam35-223 gDNA**  **-----------------ATCAACTACAATTTTGAGTGAGTAGAAAGAACCGACGCTGACCTTAGTGGTGCCCCAAGCAGCACCAAGGAAGTACCAAATAG**

**fam46 male gDNA**  **-----------------ATCAACTACAATTTTGAGTGAGTAGAAAGAACCGACGCTGACCTTAGTGGTGCCCCAAGCAGCACCAAGGAAGTACCAAATAG**

**fam46-114 gDNA**  **-----------------ATCAACTACAATTTTGAGTGAGTAGAAAGAACCGACGCTGACCTTAGTGGTGCCCCAAGCAGCACCAAGGAAGTACCAAATAG**

**fam46-115 gDNA**  **-----------------ATCAACTACAATTTTGAGTGAGTAGAAAGAACCGACGCTGACCTTAGTGGTGCCCCAAGCAGCACCAAGGAAGTACCAAATAG**

**fam46-116 gDNA**  **----------------------------------------------------------------------------------------------------**

110 120 130 140 150 160 170 180 190 200

....|....|....|....|....|....|....|....|....|....|....|....|....|....|....|....|....|....|....|....|

**L allel gDNA**  **TGTATCCGAACCTTATAACGTTTGGATACTGGCACATAGCTGGTCTTTATGGCCTCTACTTGTGCTTCACTTCTGCTAAATGGGCTACAATTTTATTTA<**

**L allel cDNA**  **TGTATCCGAACCTTATAACGTTTGGATACTGGCACATAGCTGGTCTTTATGGCCTCTACTTGTGCTTCACTTCTGCTAAATGGGCTACAATTTTATTTA<**

**H allel fam35-07 gDNA** **TGTACCCGAACCTTATAACGTTTGGTTACTGGCACATAGCTGGTCTTTATGGCCTTTACTTGTGCTTCATTTCTGCTAAATGGGCTACAATTTTATTTA<**

**H56 01A**  **TGTATCCGAACCTTATAACGTTTGGATACTGGCACATAGCTGGTCTTTATGGCCTCTACTTGTGCTTCACTTCTGCTAAATGGGCTACAATTTTATTTA<**

**H56 01B**  **TGTATCCGAACCTTATAACGTTTGGATACTGGCACATAGCTGGTCTTTATGGCCTCTACTTGTGCTTCACTTCTGCTAAATGGGCTACAATTTTATTTA<**

**H56 01G**  **TGTATCCGAACCTTATAACGTTTGGATACTGGCACATAGCTGGTCTTTATGGCCTCTACTTGTGCTTCACTTCTGCTAAATGGGCTACAATTTTATTTA<**

**H56 04A**  **TGTAyCCGAACCTTATAACGTTTGGwTACTGGCACATAGC**

**H56 04C**  **TGTAyCCGAACCTTATAACGTTTGGwTACTGGCACATAGC**

**H56 04D**  **TGTATCCGAACCTTATAACGTTTGGATACTGGCACATAGC**

**H56 07A**  **TGTAyCCGAACCTTATAACGTTTGGwTACTGGCACATAGC**

**H56 07B**  **TGTAyCCGAACCTTATAACGTTTGGwTACTGGCACATAGC**

**H56 07C**  **TGTAyCCGAACCTTATAACGTTTGGwTACTGGCACATAGC**

**H56 07F**  **TGTACCCGAACCTTATAACGTTTGGTTACTGGCACATAGC**

**H56 07G**  **TGTACCCGAACCTTATAACGTTTGGTTACTGGCACATAGC**

**H56 10A**  **TGTAyCCGAACCTTATAACGTTTGGwTACTGGCACATAGC**

**H56 10F**  **TGTAyCCGAACCTTATAACGTTTGGwTACTGGCACATAGC**

**H56 10H**  **TGTACCCGAACCTTATAACGTTTGGTTACTGGCACATAGC**

**H56 12A**  **TGTAyCCGAACCTTATAACGTTTGGwTACTGGCACATAGC**

**H56 12B**  **TGTATCCGAACCTTATAACGTTTGGATACTGGCACATAGC**

**H56 12C**  **TGTAyCCGAACCTTATAACGTTTGGwTACTGGCACATAGC**

**H56 12D**  **TGTAyCCGAACCTTATAACGTTTGGwTACTGGCACATAGC**

**9173 gDNA**  **TGTATCCGAACCTTATAACGTTTGGATACTGGCACATAGCTGGTCTTTATGGCCTCTACTTGTGCTTCACTTCTGCTAAATGGGCTACAATTTTATTTA<**

**9183 gDNA**  **TGTATCCGAACCTTATAACGTTTGGATACTGGCACATAGCTGGTCTTTATGGCCTCTACTTGTGCTTCACTTCTGCTAAATGGGCTACAATTTTATTTA<**

**9185 gDNA**  **----------------------------------------------------------------------------------------------------**

**10103 gDNA**  **----------------------------------------------------------------------------------------------------**

**1082 gDNA**  **----------------------------------------------------------------------------------------------------**

**1351 gDNA**  **----------------------------------------------------------------------------------------------------**

**1364 gDNA**  **----------------------------------------------------------------------------------------------------**

**14142 gDNA**  **----------------------------------------------------------------------------------------------------**

**1434 gDNA**  **----------------------------------------------------------------------------------------------------**

**15109 gDNA**  **TGTATCCGAACCTTATAACGTTTGGATACTGGCACATAGCTGGTCTTTATGGCCTCTACTTGTGCTTCACTTCTGCTAAATGGGCTACAATTTTATTTA<**

**15282 gDNA**  **TGTATCCGAACCTTATAACGTTTGGATACTGGCACATAGCTGGTCTTTATGGCCTCTACTTGTGCTTCACTTCTGCTAAATGGGCTACAATTTTATTTA<**

**1560 gDNA**  **TGTATCCGAACCTTATAACGTTTGGATACTGGCACATAGCTGGTCTTTATGGCCTCTACTTGTGCTTCACTTCTGCTAAATGGGCTACAATTTTATTTA<**

**1581 gDNA**  **TGTATCCGAACCTTATAACGTTTGGATACTGGCACATAGCTGGTCTTTATGGCCTCTACTTGTGCTTCACTTCTGCTAAATGGGCTACAATTTTATTTA<**

**1582 gDNA**  **TGTATCCGAACCTTATAACGTTTGGATACTGGCACATAGCTGGTCTTTATGGCCTCTACTTGTGCTTCACTTCTGCTAAATGGGCTACAATTTTATTTA<**

**2816 cDNA**  **TGTATCCGAACCTTATAACGTTTGGATACTGGCACATAGCTGGTCTTTATGGCCTCTACTTGTGCTTCACTTCTGCTAAATGGGCTACAATTTTATTTA<**

**2844 cDNA**  **TGTATCCGAACCTTATAACGTTTGGATACTGGCACATAGCTGGTCTTTATGGCCTCTACTTGTGCTTCACTTCTGCTAAATGGGCTACAATTTTATTTA<**

**2848 cDNA**  **TGTATCCGAACCTTATAACGTTTGGATACTGGCACATAGCTGGTCTTTATGGCCTCTACTTGTGCTTCACTTCTGCTAAATGGGCTACAATTTTATTTA<**

**fam35 female gDNA**  **TGTATCCGAACCTTATAACGTTTGGATACTGGCACATAGCTGGTCTTTATGGCCTCTACTTGTGCTTCACTTCTGCTAAATGGGCTACAATTTTATTTA<**

**fam35-06 cDNA**  **TGTATCCGAACCTTATAACGTTTGGATACTGGCACATAGCTGGTCtTTATGGCCTCTACTTGTGCTTCACTTCTGCTAAATGGGCTACAATTTTATTTA<**

**fam35-06 gDNA**  **TGTATCCGAACCTTATAACGTTTGGATACTGGCACATAGCTGGTCTTTATGGCCTCTACTTGTGCTTCACTTCTGCTAAATGGGCTACAATTTTATTTA<**

**fam35-08 cDNA**  **TGTATCCGAACCTTATAACGTTTGGATACTGGCACATAGCTGGTCTTTATGGCCTCTACTTGTGCTTCACTTCTGCTAAATGGGCTACAATTTTATTTA<**

**fam35-08 gDNA**  **TGTATCCGAACCTTATAACGTTTGGATACTGGCACATAGCTGGTCTTTATGGCCTCTACTTGTGCTTCACTTCTGCTAAATGGGCTACAATTTTATTTA<**

**fam35-223 cDNA**  **-----------------ACGTTtGGATACTGGCACATAGCTGGTCTTTATGGCCTCTACTTGTGCTTCACTTCTGCTAAATGGGCTACAATTTTATTTA<**

**fam35-223 gDNA**  **TGTATCCGAACCTTATAACGTTTGGATACTGGCACATAGCTGGTCTTTATGGCCTCTACTTGTGCTTCACTTCTGCTAAATGGGCTACAATTTTATTTA<**

**fam46 male gDNA**  **TGTATCCGAACCTTATAACGTTTGGATACTGGCACATAGCTGGTCTTTATGGCCTCTACTTGTGCTTCACTTCTGCTAAATGGGCTACAATTTTATTTA<**

**fam46-114 gDNA**  **TGTATCCGAACCTTATAACGTTTGGATACTGGCACATAGCTGGTCTTTATGGCCTCTACTTGTGCTTCACTTCTGCTAAATGGGCTACAATTTTATTTA<**

**fam46-115 gDNA**  **TGTATCCGAACCTTATAACGTTTGGATACTGGCACATAGCTGGTCTTTATGGCCTCTACTTGTGCTTCACTTCTGCTAAATGGGCTACAATTTTATTTA<**

**fam46-116 gDNA**  **----------------------------------------------------------------------------------------------------**

210 220 230 240 250 260 270 280 290 300

....|....|....|....|....|....|....|....|....|....|....|....|....|....|....|....|....|....|....|....|

**L allel gDNA**  **GTAAGTTATGCAGTTTTTTTTTACATCAGAGTTACGTAAAGTAG----TCCTGAAAAGATTATAATCACTTTATTA--CCTCCACCGCCTCGATGGTCTA**

**L allel cDNA**  **----------------------------------------------------------------------------------------------------**

**H allel fam35-07 gDNA** **GTAAGTTAAGAAGTTTTTTT--ACATCAAAGTTACGTAAAGTACTTAGTCCTGAAAAGATTGTAATCACTTTATTATGCCTCGACTGCCACGATGGTCTA**

**H56 01A**  **GTAAGTTATGCAGTTTTTTTTTACATCAGAGTTACGTAAAGTAG----TCCTGAAAAGATTATAATCACTTTATTA--CCTCCACCGCCTCGATGGTCTA**

**H56 01B**  **GTAAGTTATGCAGTTTTTTTTTACATCAGAGTTACGTAAAGTAG----TCCTGAAAAGATTATAATCACTTTATTA--CCTCCACCGCCTCGATGGTCTA**

**H56 01G**  **GTAAGTTATGCAGTTTTTTTTTACATCAGAGTTACGTAAAGTAG----TCCTGAAAAGATTATAATCACTTTATTA--CCTCCACCGCCTCGATGGTCTA**

**H56 04A**

**H56 04C**

**H56 04D**

**H56 07A**

**H56 07B**

**H56 07C**

**H56 07F**

**H56 07G**

**H56 10A**

**H56 10F**

**H56 10H**

**H56 12A**

**H56 12B**

**H56 12C**

**H56 12D**

**9173 gDNA**  **GTAAGTTATGCAGTTTTTTTTTACATCAGAGTTACGTAAAGTAG----TCCTGAAAAGATTATAATCACTTTATTA--CCTCCACCGCCTCGATGGTCTA**

**9183 gDNA**  **GTAAGTTATGCAGTTTTTTTTTACATCAGAGTTACGTAAAGTAG----TCCTGAAAAGATTATAATCACTTTATTA--CCTCCACCGCCTCGATGGTCTA**

**9185 gDNA**  **----------------------------------------------------------------------------------------------------**

**10103 gDNA**  **----------------------------------------------------------------------------------------------------**

**1082 gDNA**  **----------------------------------------------------------------------------------------------------**

**1351 gDNA**  **----------------------------------------------------------------------------------------------------**

**1364 gDNA**  **----------------------------------------------------------------------------------------------------**

**14142 gDNA**  **----------------------------------------------------------------------------------------------------**

**1434 gDNA**  **----------------------------------------------------------------------------------------------------**

**15109 gDNA**  **GTAAGTTATGCAGTTTTTTTTTACATCAGAGTTACGTAAAGTAG----TCCTGAAAAGATTATAATCACTTTATTA--CCTCCACCGCCTCGATGGTCTA**

**15282 gDNA**  **GTAAGTTATGCAGTTTTTTTTTACATCAGAGTTACGTAAAGTAG----TCCTGAAAAGATTATAATCACTTTATTA--CCTCCACCGCCTCGATGGTCTA**

**1560 gDNA**  **GTAAGTTATGCAGTTTTTTTTTACATCAGAGTTACGTAAAGTAG----TCCTGAAAAGATTATAATCACTTTATTA--CCTCCACCGCCTCGATGGTCTA**

**1581 gDNA**  **GTAAGTTATGCAGTTTTTTTTTACATCAGAGTTACGTAAAGTAG----TCCTGAAAAGATTATAATCACTTTATTA--CCTCCACCGCCTCGATGGTCTA**

**1582 gDNA**  **GTAAGTTATGCAGTTTTTTTTTACATCAGAGTTACGTAAAGTAG----TCCTGAAAAGATTATAATCACTTTATTA--CCTCCACCGCCTCGATGGTCTA**

**2816 cDNA**  **----------------------------------------------------------------------------------------------------**

**2844 cDNA**  **----------------------------------------------------------------------------------------------------**

**2848 cDNA**  **----------------------------------------------------------------------------------------------------**

**fam35 female gDNA**  **GTAAGTTATGCAGTTTTTTTTTACATCAGAGTTACGTAAAGTAG----TCCTGAAAAGATTATAATCACTTTATTA--CCTCCACCGCCTCGATGGTCTA**

**fam35-06 cDNA**  **----------------------------------------------------------------------------------------------------**

**fam35-06 gDNA**  **GTAAGTTATGCAGTTTTTTTTTACATCAGAGTTACGTAAAGTAG----TCCTGAAAAGATTATAATCACTTTATTA--CCTCCACCGCCTCGATGGTCTA**

**fam35-08 cDNA**  **----------------------------------------------------------------------------------------------------**

**fam35-08 gDNA**  **GTAAGTTATGCAGTTTTTTTTTACATCAGAGTTACGTAAAGTAG----TCCTGAAAAGATTATAATCACTTTATTA--CCTCCACCGCCTCGATGGTCTA**

**fam35-223 cDNA**  **----------------------------------------------------------------------------------------------------**

**fam35-223 gDNA**  **GTAAGTTATGCAGTTTTTTTTTACATCAGAGTTACGTAAAGTAG----TCCTGAAAAGATTATAATCACTTTATTA--CCTCCACCGCCTCGATGGTCTA**

**fam46 male gDNA**  **GTAAGTTATGCAGTTTTTTTTTACATCAGAGTTACGTAAAGTAG----TCCTGAAAAGATTATAATCACTTTATTA--CCTCCACCGCCTCGATGGTCTA**

**fam46-114 gDNA**  **GTAAGTTATGCAGTTTTTTTTTACATCAGAGTTACGTAAAGTAG----TCCTGAAAAGATTATAATCACTTTATTA--CCTCCACCGCCTCGATGGTCTA**

**fam46-115 gDNA**  **GTAAGTTATGCAGTTTTTTTTTACATCAGAGTTACGTAAAGTAG----TCCTGAAAAGATTATAATCACTTTATTA--CCTCCACCGCCTCGATGGTCTA**

**fam46-116 gDNA**  **----------------------------------------------------------------------------------------------------**

310 320 330 340 350 360 370 380 390 400

....|....|....|....|....|....|....|....|....|....|....|....|....|....|....|....|....|....|....|....|

**L allel gDNA**  **GTGGTCGCATAGCGCGACTGCCTTGCTACGGGTCTCGGGTTCGATTCCCGGGTCAGGCCGAAATCGCTTTGTGGTTTTAAGAAACTTTCACAAAGCAGCC**

**L allel cDNA**  **----------------------------------------------------------------------------------------------------**

**H allel fam35-07 gDNA** **GTGGTCGCATAGCGCGACTGCCGTGCCACGGGTCTCGGGTTCGATTCCCGGGTCAGGCCGAAATCGCTTTGTGGTTTTAAGAAACTTTCACAAAGCAGCC**

**H56 01A**  **GTGGTCGCATAGCGCGACTGCCTTGCTACGGGTCTCGGGTTCGATTCCCGGGTCAGGCCGAAATCGCTTTGTGGTTTTAAGAAACTTTCACAAAGCAGCC**

**H56 01B**  **GTGGTCGCATAGCGCGACTGCCTTGCTACGGGTCTCGGGTTCGATTCCCGGGTCAGGCCGAAATCGCTTTGTGGTTTTAAGAAACTTTCACAAAGCAGCC**

**H56 01G**  **GTGGTCGCATAGCGCGACTGCCTTGCTACGGGTCTCGGGTTCGATTCCCGGGTCAGGCCGAAATCGCTTTGTGGTTTTAAGAAACTTTCACAAAGCAGCC**

**H56 04A**

**H56 04C**

**H56 04D**

**H56 07A**

**H56 07B**

**H56 07C**

**H56 07F**

**H56 07G**

**H56 10A**

**H56 10F**

**H56 10H**

**H56 12A**

**H56 12B**

**H56 12C**

**H56 12D**

**9173 gDNA**  **GTGGTCGCATAGCGCGACTGCCTTGCTACGGGTCTCGGGTTCGATTCCCGGGTCAGGCCGAAATCGCTTTGTGGTTTTAAGAAACTTTCACAAAGCAGCC**

**9183 gDNA**  **GTGGTCGCATAGCGCGACTGCCTTGCTACGGGTCTCGGGTTCGATTCCCGGGTCAGGCCGAAATCGCTTTGTGGTTTTAAGAAACTTTCACAAAGCAGCC**

**9185 gDNA**  **----------------------------------------------------------------------------------------------------**

**10103 gDNA**  **----------------------------------------------------------------------------------------------------**

**1082 gDNA**  **----------------------------------------------------------------------------------------------------**

**1351 gDNA**  **----------------------------------------------------------------------------------------------------**

**1364 gDNA**  **----------------------------------------------------------------------------------------------------**

**14142 gDNA**  **----------------------------------------------------------------------------------------------------**

**1434 gDNA**  **----------------------------------------------------------------------------------------------------**

**15109 gDNA**  **GTGGTCGCATAGCGCGACTGCCTTGCTACGGGTCTCGGGTTCGATTCCCGGGTCAGGCCGAAATCGCTTTGTGGTTTTAAGAAACTTTCACAAAGCAGCC**

**15282 gDNA**  **GTGGTCGCATAGCGCGACTGCCTTGCTACGGGTCTCGGGTTCGATTCCCGGGTCAGGCCGAAATCGCTTTGTGGTTTTAAGAAACTTTCACAAAGCAGCC**

**1560 gDNA**  **GTGGTCGCATAGCGCGACTGCCTTGCTACGGGTCTCGGGTTCGATTCCCGGGTCAGGCCGAAATCGCTTTGTGGTTTTAAGAAACTTTCACAAAGCAGCC**

**1581 gDNA**  **GTGGTCGCATAGCGCGACTGCCTTGCTACGGGTCTCGGGTTCGATTCCCGGGTCAGGCCGAAATCGCTTTGTGGTTTTAAGAAACTTTCACAAAGCAGCC**

**1582 gDNA**  **GTGGTCGCATAGCGCGACTGCCTTGCTACGGGTCTCGGGTTCGATTCCCGGGTCAGGCCGAAATCGCTTTGTGGTTTTAAGAAACTTTCACAAAGCAGCC**

**2816 cDNA**  **----------------------------------------------------------------------------------------------------**

**2844 cDNA**  **----------------------------------------------------------------------------------------------------**

**2848 cDNA**  **----------------------------------------------------------------------------------------------------**

**fam35 female gDNA**  **GTGGTCGCATAGCGCGACTGCCTTGCTACGGGTCTCGGGTTCGATTCCCGGGTCAGGCCGAAATCGCTTTGTGGTTTTAAGAAACTTTCACAAAGCAGCC**

**fam35-06 cDNA**  **----------------------------------------------------------------------------------------------------**

**fam35-06 gDNA**  **GTGGTCGCATAGCGCGACTGCCTTGCTACGGGTCTCGGGTTCGATTCCCGGGTCAGGCCGAAATCGCTTTGTGGTTTTAAGAAACTTTCACAAAGCAGCC**

**fam35-08 cDNA**  **----------------------------------------------------------------------------------------------------**

**fam35-08 gDNA**  **GTGGTCGCATAGCGCGACTGCCTTGCTACGGGTCTCGGGTTCGATTCCCGGGTCAGGCCGAAATCGCTTTGTGGTTTTAAGAAACTTTCACAAAGCAGCC**

**fam35-223 cDNA**  **----------------------------------------------------------------------------------------------------**

**fam35-223 gDNA**  **GTGGTCGCATAGCGCGACTGCCTTGCTACGGGTCTCGGGTTCGATTCCCGGGTCAGGCCGAAATCGCTTTGTGGTTTTAAGAAACTTTCACAAAGCAGCC**

**fam46 male gDNA**  **GTGGTCGCATAGCGCGACTGCCTTGCTACGGGTCTCGGGTTCGATTCCCGGGTCAGGCCGAAATCGCTTTGTGGTTTTAAGAAACTTTCACAAAGCAGCC**

**fam46-114 gDNA**  **GTGGTCGCATAGCGCGACTGCCTTGCTACGGGTCTCGGGTTCGATTCCCGGGTCAGGCCGAAATCGCTTTGTGGTTTTAAGAAACTTTCACAAAGCAGCC**

**fam46-115 gDNA**  **GTGGTCGCATAGCGCGACTGCCTTGCTACGGGTCTCGGGTTCGATTCCCGGGTCAGGCCGAAATCGCTTTGTGGTTTTAAGAAACTTTCACAAAGCAGCC**

**fam46-116 gDNA**  **----------------------------------------------------------------------------------------------------**

410 420 430 440 450 460 470 480 490 500

....|....|....|....|....|....|....|....|....|....|....|....|....|....|....|....|....|....|....|....|

**L allel gDNA**  **TGGAGTTTGGGAGTTGGCGGTGTTACACCCCCTGCCTGGGAGGGCACGTAAAGCCGTCTTCAAATGCTACTCAGTAGCAGTCGTAGTTATAATTCTACAT**

**L allel cDNA**  **----------------------------------------------------------------------------------------------------**

**H allel fam35-07 gDNA** **TGGAGTTTGGGAGTTGGCGGTGTTACACCACCTGCCTGGGAGGGCACGTAAAGCCGTCTTCAAATGCTACTCAGTAGCAGTCGTAGTTATAATTCTACAT**

**H56 01A**  **TGGAGTTTGGGAGTTGGCGGTGTTACACCCCCTGCCTGGGAGGGCACGTAAAGCCGTCTTCAAATGCTACTCAGTAGCAGTCGTAGTTATAATTCTACAT**

**H56 01B**  **TGGAGTTTGGGAGTTGGCGGTGTTACACCCCCTGCCTGGGAGGGCACGTAAAGCCGTCTTCAAATGCTACTCAGTAGCAGTCGTAGTTATAATTCTACAT**

**H56 01G**  **TGGAGTTTGGGAGTTGGCGGTGTTACACCCCCTGCCTGGGAGGGCACGTAAAGCCGTCTTCAAATGCTACTCAGTAGCAGTCGTAGTTATAATTCTACAT**

**H56 04A**

**H56 04C**

**H56 04D**

**H56 07A**

**H56 07B**

**H56 07C**

**H56 07F**

**H56 07G**

**H56 10A**

**H56 10F**

**H56 10H**

**H56 12A**

**H56 12B**

**H56 12C**

**H56 12D**

**9173 gDNA**  **TGGAGTTTGGGAGTTGGCGGTGTTACACCCCCTGCCTGGGAGGGCACGTAAAGCCGTCTTCAAATGCTACTCAGTAGCAGTCGTAGTTATAATTCTACAT**

**9183 gDNA**  **TGGAGTTTGGGAGTTGGCGGTGTTACACCCCCTGCCTGGGAGGGCACGTAAAGCCGTCTTCAAATGCTACTCAGTAGCAGTCGTAGTTATAATTCTACAT**

**9185 gDNA**  **----------------------------------------------------------------------------------------------------**

**10103 gDNA**  **----------------------------------------------------------------------------------------------------**

**1082 gDNA**  **----------------------------------------------------------------------------------------------------**

**1351 gDNA**  **----------------------------------------------------------------------------------------------------**

**1364 gDNA**  **----------------------------------------------------------------------------------------------------**

**14142 gDNA**  **----------------------------------------------------------------------------------------------------**

**1434 gDNA**  **----------------------------------------------------------------------------------------------------**

**15109 gDNA**  **TGGAGTTTGGGAGTTGGCGGTGTTACACCCCCTGCCTGGGAGGGCACGTAAAGCCGTCTTCAAATGCTACTCAGTAGCAGTCGTAGTTATAATTCTACAT**

**15282 gDNA**  **TGGAGTTTGGGAGTTGGCGGTGTTACACCCCCTGCCTGGGAGGGCACGTAAAGCCGTCTTCAAATGCTACTCAGTAGCAGTCGTAGTTATAATTCTACAT**

**1560 gDNA**  **TGGAGTTTGGGAGTTGGCGGTGTTACACCCCCTGCCTGGGAGGGCACGTAAAGCCGTCTTCAAATGCTACTCAGTAGCAGTCGTAGTTATAATTCTACAT**

**1581 gDNA**  **TGGAGTTTGGGAGTTGGCGGTGTTACACCCCCTGCCTGGGAGGGCACGTAAAGCCGTCTTCAAATGCTACTCAGTAGCAGTCGTAGTTATAATTCTACAT**

**1582 gDNA**  **TGGAGTTTGGGAGTTGGCGGTGTTACACCCCCTGCCTGGGAGGGCACGTAAAGCCGTCTTCAAATGCTACTCAGTAGCAGTCGTAGTTATAATTCTACAT**

**2816 cDNA**  **----------------------------------------------------------------------------------------------------**

**2844 cDNA**  **----------------------------------------------------------------------------------------------------**

**2848 cDNA**  **----------------------------------------------------------------------------------------------------**

**fam35 female gDNA**  **TGGAGTTTGGGAGTTGGCGGTGTTACACCCCCTGCCTGGGAGGGCACGTAAAGCCGTCTTCAAATGCTACTCAGTAGCAGTCGTAGTTATAATTCTACAT**

**fam35-06 cDNA**  **----------------------------------------------------------------------------------------------------**

**fam35-06 gDNA**  **TGGAGTTTGGGAGTTGGCGGTGTTACACCCCCTGCCTGGGAGGGCACGTAAAGCCGTCTTCAAATGCTACTCAGTAGCAGTCGTAGTTATAATTCTACAT**

**fam35-08 cDNA**  **----------------------------------------------------------------------------------------------------**

**fam35-08 gDNA**  **TGGAGTTTGGGAGTTGGCGGTGTTACACCCCCTGCCTGGGAGGGCACGTAAAGCCGTCTTCAAATGCTACTCAGTAGCAGTCGTAGTTATAATTCTACAT**

**fam35-223 cDNA**  **----------------------------------------------------------------------------------------------------**

**fam35-223 gDNA**  **TGGAGTTTGGGAGTTGGCGGTGTTACACCCCCTGCCTGGGAGGGCACGTAAAGCCGTCTTCAAATGCTACTCAGTAGCAGTCGTAGTTATAATTCTACAT**

**fam46 male gDNA**  **TGGAGTTTGGGAGTTGGCGGTGTTACACCCCCTGCCTGGGAGGGCACGTAAAGCCGTCTTCAAATGCTACTCAGTAGCAGTCGTAGTTATAATTCTACAT**

**fam46-114 gDNA**  **TGGAGTTTGGGAGTTGGCGGTGTTACACCCCCTGCCTGGGAGGGCACGTAAAGCCGTCTTCAAATGCTACTCAGTAGCAGTCGTAGTTATAATTCTACAT**

**fam46-115 gDNA**  **TGGAGTTTGGGAGTTGGCGGTGTTACACCCCCTGCCTGGGAGGGCACGTAAAGCCGTCTTCAAATGCTACTCAGTAGCAGTCGTAGTTATAATTCTACAT**

**fam46-116 gDNA**  **----------------------------------------------------------------------------------------------------**

510 520 530 540 550 560 570 580 590 600

....|....|....|....|....|....|....|....|....|....|....|....|....|....|....|....|....|....|....|....|

**L allel gDNA**  **CTATAATTAAGGATTTGAGAAAACTCTGACACTAGGGCTTGTTAGGTGATTAAAAACCTGCAGCTCACTCGATAAGAAGAAGAACTTTATTATGTTATGT**

**L allel cDNA**  **----------------------------------------------------------------------------------------------------**

**H allel fam35-07 gDNA** **CTATAATTAAGGATTTGAGAAAAATCTGACACTAGGGCTTGTTAGGTGATTAAAAACCTGCAGCTCACTCGATAAGAAGAAGAACTTTATTATGTTATGT**

**H56 01A**  **CTATAATTAAGGATTTGAGAAAACTCTGACACTAGGGCTTGTTAGGTGATTAAAAACCTGCAGCTCACTCGATAAGAAGAAGAACTTTATTATGTTATGT**

**H56 01B**  **CTATAATTAAGGATTTGAGAAAACTCTGACACTAGGGCTTGTTAGGTGATTAAAAACCTGCAGCTCACTCGATAAGAAGAAGAACTTTATTATGTTATGT**

**H56 01G**  **CTATAATTAAGGATTTGAGAAAACTCTGACACTAGGGCTTGTTAGGTGATTAAAAACCTGCAGCTCACTCGATAAGAAGAAGAACTTTATTATGTTATGT**

**H56 04A**

**H56 04C**

**H56 04D**

**H56 07A**

**H56 07B**

**H56 07C**

**H56 07F**

**H56 07G**

**H56 10A**

**H56 10F**

**H56 10H**

**H56 12A**

**H56 12B**

**H56 12C**

**H56 12D**

**9173 gDNA**  **CTATAATTAAGGATTTGAGAAAACTCTGACACTAGGGCTTGTTAGGTGATTAAAAACCTGCAGCTCACTCGATAAGAAGAAGAACTTTATTATGTTATGT**

**9183 gDNA**  **CTATAATTAAGGATTTGAGAAAACTCTGACACTAGGGCTTGTTAGGTGATTAAAAACCTGCAGCTCACTCGATAAGAAGAAGAACTTTATTATGTTATGT**

**9185 gDNA**  **----------------------------------------------------------------------------------------------------**

**10103 gDNA**  **----------------------------------------------------------------------------------------------------**

**1082 gDNA**  **----------------------------------------------------------------------------------------------------**

**1351 gDNA**  **----------------------------------------------------------------------------------------------------**

**1364 gDNA**  **----------------------------------------------------------------------------------------------------**

**14142 gDNA**  **----------------------------------------------------------------------------------------------------**

**1434 gDNA**  **----------------------------------------------------------------------------------------------------**

**15109 gDNA**  **CTATAATTAAGGATTTGAGAAAACTCTGACACTAGGGCTTGTTAGGTGATTAAAAACCTGCAGCTCACTCGATAAGAAGAAGAACTTTATTATGTTATGT**

**15282 gDNA**  **CTATAATTAAGGATTTGAGAAAACTCTGACACTAGGGCTTGTTAGGTGATTAAAAACCTGCAGCTCACTCGATAAGAAGAAGAACTTTATTATGTTATGT**

**1560 gDNA**  **CTATAATTAAGGATTTGAGAAAACTCTGACACTAGGGCTTGTTAGGTGATTAAAAACCTGCAGCTCACTCGATAAGAAGAAGAACTTTATTATGTTATGT**

**1581 gDNA**  **CTATAATTAAGGATTTGAGAAAACTCTGACACTAGGGCTTGTTAGGTGATTAAAAACCTGCAGCTCACTCGATAAGAAGAAGAACTTTATTATGTTATGT**

**1582 gDNA**  **CTATAATTAAGGATTTGAGAAAACTCTGACACTAGGGCTTGTTAGGTGATTAAAAACCTGCAGCTCACTCGATAAGAAGAAGAACTTTATTATGTTATGT**

**2816 cDNA**  **----------------------------------------------------------------------------------------------------**

**2844 cDNA**  **----------------------------------------------------------------------------------------------------**

**2848 cDNA**  **----------------------------------------------------------------------------------------------------**

**fam35 female gDNA**  **CTATAATTAAGGATTTGAGAAAACTCTGACACTAGGGCTTGTTAGGTGATTAAAAACCTGCAGCTCACTCGATAAGAAGAAGAACTTTATTATGTTATGT**

**fam35-06 cDNA**  **----------------------------------------------------------------------------------------------------**

**fam35-06 gDNA**  **CTATAATTAAGGATTTGAGAAAACTCTGACACTAGGGCTTGTTAGGTGATTAAAAACCTGCAGCTCACTCGATAAGAAGAAGAACTTTATTATGTTATGT**

**fam35-08 cDNA**  **----------------------------------------------------------------------------------------------------**

**fam35-08 gDNA**  **CTATAATTAAGGATTTGAGAAAACTCTGACACTAGGGCTTGTTAGGTGATTAAAAACCTGCAGCTCACTCGATAAGAAGAAGAACTTTATTATGTTATGT**

**fam35-223 cDNA**  **----------------------------------------------------------------------------------------------------**

**fam35-223 gDNA**  **CTATAATTAAGGATTTGAGAAAACTCTGACACTAGGGCTTGTTAGGTGATTAAAAACCTGCAGCTCACTCGATAAGAAGAAGAACTTTATTATGTTATGT**

**fam46 male gDNA**  **CTATAATTAAGGATTTGAGAAAACTCTGACACTAGGGCTTGTTAGGTGATTAAAAACCTGCAGCTCACTCGATAAGAAGAAGAACTTTATTATGTTATGT**

**fam46-114 gDNA**  **CTATAATTAAGGATTTGAGAAAACTCTGACACTAGGGCTTGTTAGGTGATTAAAAACCTGCAGCTCACTCGATAAGAAGAAGAACTTTATTATGTTATGT**

**fam46-115 gDNA**  **CTATAATTAAGGATTTGAGAAAACTCTGACACTAGGGCTTGTTAGGTGATTAAAAACCTGCAGCTCACTCGATAAGAAGAAGAACTTTATTATGTTATGT**

**fam46-116 gDNA**  **----------------------------------------------------------------------------------------------------**

610 620 630 640 650 660 670 680 690 700

....|....|....|....|....|....|....|....|....|....|....|....|....|....|....|....|....|....|....|....|

**L allel gDNA**  **GTTGCCAGTAT--GTAACATGAA-T----ATAAACACATGTCTAAAAATATCAAAATAATTTTCAG>GTTACTTCCTCTTCGTGGTCGCAGAAATAGGGA**

**L allel cDNA**  **------------------------------------------------------------------>GTTACTTCCTCTTCGTGGTCGCAGAAATAGGGA**

**H allel fam35-07 gDNA** **TTTGCTAGTATAAGTA-CGTGAAATGAAGATAACCACATCTCTAAAAATATCAAAATAATTTTCAG>GTTACTTCCTCTTCGTGGTTGCAGAAATAGGGA**

**H56 01A**  **GTTGCCAGTAT--GTAACATGAA-T----ATAAACACATGTCTAAAAATATCAAAATAATTTTCAG>GTTACTTCCTCTTCGTGGTCGCAGAAATAGGGA**

**H56 01B**  **GTTGCCAGTAT--GTAACATGAA-T----ATAAACACATGTCTAAAAATATCAAAATAATTTTCAG>GTTACTTCCTCTTCGTGGTCGCAGAAATAGGGA**

**H56 01G**  **GTTGCCAGTAT--GTAACATGAA-T----ATAAACACATGTCTAAAAATATCAAAATAATTTTCAG>GTTACTTCCTCTTCGTGGTCGCAGAAATAGGGA**

**H56 04A**

**H56 04C**

**H56 04D**

**H56 07A**

**H56 07B**

**H56 07C**

**H56 07F**

**H56 07G**

**H56 10A**

**H56 10F**

**H56 10H**

**H56 12A**

**H56 12B**

**H56 12C**

**H56 12D**

**9173 gDNA**  **GTTGCCAGTAT--GTAACATGAA-T----ATAAACACATGTCTAAAAATATCAAAATAAT**

**9183 gDNA**  **GTTGCCAGTAT--GTAACATGAA-T----ATAAACACATGTCTAAAAATATCAAAATAAT**

**9185 gDNA**  **----------------------------------------------------------------------------------------------------**

**10103 gDNA**  **----------------------------------------------------------------------------------------------------**

**1082 gDNA**  **----------------------------------------------------------------------------------------------------**

**1351 gDNA**  **----------------------------------------------------------------------------------------------------**

**1364 gDNA**  **----------------------------------------------------------------------------------------------------**

**14142 gDNA**  **----------------------------------------------------------------------------------------------------**

**1434 gDNA**  **----------------------------------------------------------------------------------------------------**

**15109 gDNA**  **GTTGCCAGTAT--GTAACATGAA-T----ATAAACACATGTCTAAAAATATCAAAATAATxxxxxxxxxxxxxxxxxxxxxxxxxxxxxxxxxxxxxxxx**

**15282 gDNA**  **GTTGCCAGTAT--GTAACATGAA-T----ATAAACACATGTCTAAAAATATCAAAATAAT**

**1560 gDNA**  **GTTGCCAGTAT--GTAACATGAA-T----ATAAACACATGTCTAAAAATATCAAAATAAT**

**1581 gDNA**  **GTTGCCAGTAT--GTAACATGAA-T----ATAAACACATGTCTAAAAATATCAAAATAAT**

**1582 gDNA**  **GTTGCCAGTAT--GTAACATGAA-T----ATAAACACATGTCTAAAAATATCAAAATAATxxxxxxxxxxxxxxxxxxxxxxxxxxxxxxxxxxxxxxxx**

**2816 cDNA**  **------------------------------------------------------------------>GTTACTTCCTCTTCGTGGTCGCAGAAATAGGGA**

**2844 cDNA**  **------------------------------------------------------------------>GTTACTTCCTCTTCGTGGTCGCAGAAATAGGGA**

**2848 cDNA**  **------------------------------------------------------------------>GTTACTTCCTCTTCGTGGTCGCAGAAATAGGGA**

**fam35 female gDNA**  **GTTGCCAGTAT--GTAACATGAA-T----ATAAACACATGTCTAAAAATATCAAAATAATTTTCAG>GTTACTTCCTCTTCGTG**

**fam35-06 cDNA**  **------------------------------------------------------------------>GTTACTTCCTCTTCGTGGTCGCAGAAATAGGGA**

**fam35-06 gDNA**  **GTTGCCAGTAT--GTAACATGAA-T----ATAAACACATGTCTAAAAATATCAAAATAATTTTCAG>GTTACTTCCTCTTCGTG**

**fam35-08 cDNA**  **------------------------------------------------------------------>GTTACTTCCTCTTCGTGGTCGCAGAAATAGGGA**

**fam35-08 gDNA**  **GTTGCCAGTAT--GTAACATGAA-T----ATAAACACATGTCTAAAAATATCAAAATAATTTTCAG>GTTACTTCCTCTTCGTG**

**fam35-223 cDNA**  **------------------------------------------------------------------>GTTACTTCCTCTTCGTGGTCGCAGAAATAGGGA**

**fam35-223 gDNA**  **GTTGCCAGTAT--GTAACATGAA-T----ATAAACACATGTCTAAAAATATCAAAAxxxxxxxxxxxxxxxxxxxxxxxxxxxxxxxxxxxxxxxxxxxx**

**fam46 male gDNA**  **GTTGCCAGTAT--GTAACATGAA-T----ATAAACACATGTCTAAAAATATCAAAATAATTTTCAG>GTTACTTCCTCTTCGTG**

**fam46-114 gDNA**  **GTTGCCAGTAT--GTAACATGAA-T----ATAAACACATGTCTAAAAATATCAAAATAAT**

**fam46-115 gDNA**  **GTTGCCAGTAT--GTAACATGAA-T----ATAAACACATGTCTAAAAATATCAAAATAATxxxxxxxxxxxxxxxxxxxxxxxxxxxxxxxxxxxxxxxx**

**fam46-116 gDNA**  **----------------------------------------------------------------------------------------------------**

710 720 730 740 750 760 770 780 790 800

....|....|....|....|....|....|....|....|....|....|....|....|....|....|....|....|....|....|....|....|

**L allel gDNA**  **TCACGGCTGGCGCTCACAGGCTATGGGCGCACAAAACTTACAAAGCGAAACTACCATTAGAAATTCTCTTAATGGTACTGAACTCCATCGCTTTTCAAAA**

**L allel cDNA**  **TCACGGCTGGCGCTCACAGGCTATGGGCGCACAAAACTTACAAAGCGAAACTACCATTAGAAATTCTCTTAATGGTACTGAACTCCATCGCTTTTCAAAA**

**H allel fam35-07 gDNA** **TCACGGCTGGCGCTCACAGGCTATGGGCGCACAAAACTTATAAAGCGAAACTACCACTAGAAATTCTCTTAATGGTACTGAACTCTATCGCTTTTCAAAA**

**H56 01A**  **TCACGGCTGGCGCTCACAGGCTATGGGCGCACAAAACTTACAAAGCGAAACTACCATTAGAAATTCTCTTAATGGTACTGAACTCCATCGCTTTTCAAAA**

**H56 01B**  **TCACGGCTGGCGCTCACAGGCTATGGGCGCACAAAACTTACAAAGCGAAACTACCATTAGAAATTCTCTTAATGGTACTGAACTCCATCGCTTTTCAAAA**

**H56 01G**  **TCACGGCTGGCGCTCACAGGCTATGGGCGCACAAAACTTACAAAGCGAAACTACCATTAGAAATTCTCTTAATGGTACTGAACTCCATCGCTTTTCAAAA**

**H56 04A**

**H56 04C**

**H56 04D**

**H56 07A**

**H56 07B**

**H56 07C**

**H56 07F**

**H56 07G**

**H56 10A**

**H56 10F**

**H56 10H**

**H56 12A**

**H56 12B**

**H56 12C**

**H56 12D**

**9173 gDNA**

**9183 gDNA**

**9185 gDNA**  **----------------------------------------------------------------------------------------------------**

**10103 gDNA**  **----------------------------------------------------------------------------------------------------**

**1082 gDNA**  **----------------------------------------------------------------------------------------------------**

**1351 gDNA**  **----------------------------------------------------------------------------------------------------**

**1364 gDNA**  **----------------------------------------------------------------------------------------------------**

**14142 gDNA**  **----------------------------------------------------------------------------------------------------**

**1434 gDNA**  **----------------------------------------------------------------------------------------------------**

**15109 gDNA**  **xxxxxxxxxxxxxxxxxxxxxxxxxxxxxxxxxxxxxxxxxxxxxxxxxxxxxxxxxxxxxxxxxxxxxxxxxxxxxxxxxxxxxxxxxxxxxxxxxxxx**

**15282 gDNA**

**1560 gDNA**

**1581 gDNA**

**1582 gDNA**  **xxxxxxxxxxxxxxxxxxxxxxxxxxxxxxxxxxxxxxxxxxxxxxxxxxxxxxxxxxxxxxxxxxxxxxxxxxxxxxxxxxxxxxxxxxxxxxxxxxxx**

**2816 cDNA**  **TCACGGCTGGCGCTCACAGGCTATGGGCGCACAAAACTTACAAAGCGAAACTACCATTAGAAATTCTCTTAATGGTACTGAACTCCATCGCTTTTCAAAA**

**2844 cDNA**  **TCACGGCTGGCGCTCACAGGCTATGGGCGCACAAAACTTACAAAGCGAAACTACCATTAGAAATTCTCTTAATGGTACTGAACTCCATCGCTTTTCAAAA**

**2848 cDNA**  **TCACGGCTGGCGCTCACAGGCTATGGGCGCACAAAACTTACAAAGCGAAACTACCATTAGAAATTCTCTTAATGGTACTGAACTCCATCGCTTTTCAAAA**

**fam35 female gDNA**

**fam35-06 cDNA**  **TCACGGCTGGCGCTCACAGGCTATGGGCGCACAAAACTTACAAAGCGAAACTACCATTAGAAATTCTCTTAATGGTACTGAACTCCATCGCTTTTCAAAA**

**fam35-06 gDNA**

**fam35-08 cDNA**  **TCACGGCTGGCGCTCACAGGCTATGGGCGCACAAAACTTACAAAGCGAAACTACCATTAGAAATTCTCTTAATGGTACTGAACTCCATCGCTTTTCAAAA**

**fam35-08 gDNA**

**fam35-223 cDNA**  **TCACGGCTGGCGCTCACAGGCTATGGGCGCACAAAACTTACAAAGCGAAACTACCATTAGAAATTCTCTTAATGGTACTGAACTCCATCGCTTTTCAAAA**

**fam35-223 gDNA**  **xxxxxxxxxxxxxxxxxxxxxxxxxxxxxxxxxxxxxxxxxxxxxxxxxxxxxxxxxxxxxxxxxxxxxxxxxxxxxxxxxxxxxxxxxxxxxxxxxxxx**

**fam46 male gDNA**

**fam46-114 gDNA**

**fam46-115 gDNA**  **xxxxxxxxxxxxxxxxxxxxxxxxxxxxxxxxxxxxxxxxxxxxxxxxxxxxxxxxxxxxxxxxxxxxxxxxxxxxxxxxxxxxxxxxxxxxxxxxxxxx**

**fam46-116 gDNA**  **----------------------------------------------------------------------------------------------------**

810 820 830 840 850 860 870 880 890 900

....|....|....|....|....|....|....|....|....|....|....|....|....|....|....|....|....|....|....|....|

**L allel gDNA**  **CTCAGCCATTGACTGGGTGAGGGACCATCGTCTCCATCATAAGTACAGCGATACTGATGCTGATCCCCATAATGCTAGCCGAGGGTTCTTTTATTCCCAT**

**L allel cDNA**  **CTCAGCCATTGACTGGGTGAGGGACCATCGTCTCCATCATAAGTACAGCGATACTGATGCTGATCCCCATAATGCTAGCCGAGGGTTCTTTTATTCCCAT**

**H allel fam35-07 gDNA** **CTCCGCCATTGACTGGGTGAGGGACCATCGTCTCCATCATAAGTACAGCGATACTGATGCTGATCCCCATAATGCTAGCCGAGGGTTCTTTTATTCCCAT**

**H56 01A**  **CTCAGCCATTGACTGGGTGAGGGACCATCGTCTCCATCATAAGTACAGCGATACTGATGCTGATCCCCATAATGCTAGCCGAGGGTTCTTTTATTCCCAT**

**H56 01B**  **CTCAGCCATTGACTGGGTGAGGGACCATCGTCTCCATCATAAGTACAGCGATACTGATGCTGATCCCCATAATGCTAGCCGAGGGTTCTTTTATTCCCAT**

**H56 01G**  **CTCAGCCATTGACTGGGTGAGGGACCATCGTCTCCATCATAAGTACAGCGATACTGATGCTGATCCCCATAATGCTAGCCGAGGGTTCTTTTATTCCCAT**

**H56 04A**

**H56 04C**

**H56 04D**

**H56 07A**

**H56 07B**

**H56 07C**

**H56 07F**

**H56 07G**

**H56 10A**

**H56 10F**

**H56 10H**

**H56 12A**

**H56 12B**

**H56 12C**

**H56 12D**

**9173 gDNA**

**9183 gDNA**

**9185 gDNA**  **----------------------------------------------------------------------------------------------------**

**10103 gDNA**  **----------------------------------------------------------------------------------------------------**

**1082 gDNA**  **----------------------------------------------------------------------------------------------------**

**1351 gDNA**  **----------------------------------------------------------------------------------------------------**

**1364 gDNA**  **----------------------------------------------------------------------------------------------------**

**14142 gDNA**  **----------------------------------------------------------------------------------------------------**

**1434 gDNA**  **----------------------------------------------------------------------------------------------------**

**15109 gDNA**  **xxxxxxxxxxxxxxxxxxxxxxxxxxxxxxxxxxxxxxxxxxxxxxxxxxxxxxxxxxxxxxxxxxxxxxxxxxxxxxxxxxxxxxxxxxxxxxxxxxxx**

**15282 gDNA**

**1560 gDNA**

**1581 gDNA**

**1582 gDNA**  **xxxxxxxxxxxxxxxxxxxxxxxxxxxxxxxxxxxxxxxxxxxxxxxxxxxxxxxxxxxxxxxxxxxxxxxxxxxxxxxxxxxxxxxxxxxxxxxxxxxx**

**2816 cDNA**  **CTCAGCCATTGACTGGGTGAGGGACCATCGTCTCCATCATAAGTACAGCGATACTGATGCTGATCCCCATAATGCTAGCCGAGGGTTCTTTTATTCCCAT**

**2844 cDNA**  **CTCAGCCATTGACTGGGTGAGGGACCATCGTCTCCATCATAAGTACAGCGATACTGATGCTGATCCCCATAATGCTAGCCGAGGGTTCTTTTATTCCCAT**

**2848 cDNA**  **CTCAGCCATTGACTGGGTGAGGGACCATCGTCTCCATCATAAGTACAGCGATACTGATGCTGATCCCCATAATGCTAGCCGAGGGTTCTTTTATTCCCAT**

**fam35 female gDNA**

**fam35-06 cDNA**  **CTCAGCCATTGACTGGGTGAGGGACCATCGTCTCCATCATAAGTACAGCGATACTGATGCTGATCCCCATAATGCTAGCCGAGGGTTCTTTTATTCCCAT**

**fam35-06 gDNA**

**fam35-08 cDNA**  **CTCAGCCATTGACTGGGTGAGGGACCATCGTCTCCATCATAAGTACAGCGATACTGATGCTGATCCCCATAATGCTAGCCGAGGGTTCTTTTATTCCCAT**

**fam35-08 gDNA**

**fam35-223 cDNA**  **CTCAGCCATTGACTGGGTGAGGGACCATCGTCTCCATCATAAGTACAGCGATACTGATGCTGATCCCCATAATGCTAGCCGAGGGTTCTTTTATTCCCAT**

**fam35-223 gDNA**  **xxxxxxxxxxxxxxxxxxxxxxxxxxxxxxxxxxxxxxxxxxxxxxxxxxxxxxxxxxxxxxxxxxxxxxxxxxxxxxxxxxxxxxxxxxxxxxxxxxxx**

**fam46 male gDNA**

**fam46-114 gDNA**

**fam46-115 gDNA**  **xxxxxxxxxxxxxxxxxxxxxxxxxxxxxxxxxxxxxxxxxxxxxxxxxxxxxxxxxxxxxxxxxxxxxxxxxxxxxxxxxxxxxxxxxxxxxxxxxxxx**

**fam46-116 gDNA**  **----------------------------------------------------------------------------------------------------**

910 920 930 940 950 960 970 980 990 1000

....|....|....|....|....|....|....|....|....|....|....|....|....|....|....|....|....|....|....|....|

**L allel gDNA**  **GTAGGATGGCTACTCGTTAGAAAACATCCTGAAGTCAAAAAACGTGGCAGAGAACTCAATATGTCTGATATCTATAACAATCCAGTGCTGCGGTTTCAGA**

**L allel cDNA**  **GTAGGATGGCTACTCGTTAGAAAACATCCTGAAGTCAAAAAACGTGGCAGAGAACTCAATATGTCTGATATCTATAACAATCCAGTGCTGCGGTTTCAGA**

**H allel fam35-07 gDNA** **GTAGGATGGCTACTCGTTAGAAAACACCCTGAAGTCAAAAAACGTGGGAGAGAACTCAATATGTCTGATATTTATAACAATCCAGTGCTGCGGTTTCAGA**

**H56 01A**  **GTAGGATGGCTACTCGTTAGAAAACATCCTGAAGTCAAAAAACGTGGCAGAGAACTCAATATGTCTGATATCTATAACAATCCAGTGCTGCGGTTTCAGA**

**H56 01B**  **GTAGGATGGCTACTCGTTAGAAAACATCCTGAAGTCAAAAAACGTGGCAGAGAACTCAATATGTCTGATATCTATAACAATCCAGTGCTGCGGTTTCAGA**

**H56 01G**  **GTAGGATGGCTACTCGTTAGAAAACATCCTGAAGTCAAAAAACGTGGCAGAGAACTCAATATGTCTGATATCTATAACAATCCAGTGCTGCGGTTTCAGA**

**H56 04A**

**H56 04C**

**H56 04D**

**H56 07A**

**H56 07B**

**H56 07C**

**H56 07F**

**H56 07G**

**H56 10A**

**H56 10F**

**H56 10H**

**H56 12A**

**H56 12B**

**H56 12C**

**H56 12D**

**9173 gDNA**

**9183 gDNA**

**9185 gDNA**  **----------------------------------------------------------------CTGATATCTATAACAATCCAGTGCTGCGGTTTCAGA**

**10103 gDNA**  **----------------------------------------------------------------CTGATATCTATAACAATCCAGTGCTGCGGTTTCAGA**

**1082 gDNA**  **----------------------------------------------------------------CTGATATCTATAACAATCCAGTGCTGCGGTTTCAGA**

**1351 gDNA**  **----------------------------------------------------------------CTGATATCTATAACAATCCAGTGCTGCGGTTTCAGA**

**1364 gDNA**  **----------------------------------------------------------------CTGATATCTATAACAATCCAGTGCTGCGGTTTCAGA**

**14142 gDNA**  **----------------------------------------------------------------CTGATATCTATAACAATCCAGTGCTGCGGTTTCAGA**

**1434 gDNA**  **----------------------------------------------------------------CTGATATCTATAACAATCCAGTGCTGCGGTTTCAGA**

**15109 gDNA**  **xxxxxxxxxxxxxxxxxxxxxxxxxxxxxxxxxxxxxxxxxxxxxxxxxxxxxxxxxxxxxxxxCTGATATCTATAACAATCCAGTGCTGCGGTTTCAGA**

**15282 gDNA**

**1560 gDNA**

**1581 gDNA**

**1582 gDNA**  **xxxxxxxxxxxxxxxxxxxxxxxxxxxxxxxxxxxxxxxxxxxxxxxxxxxxxxxxxxxxxxxxCTGATATCTATAACAATCCAGTGCTGCGGTTTCAGA**

**2816 cDNA**  **GTAGGATGGCTACTCGTTAGAAAACATCCTGAAGTCAAAAAACGTGGCAGAGAACTCAATATGTCTGATATCTATAACAATCCAGTGCTGCGGTTTCAGA**

**2844 cDNA**  **GTAGGATGGCTACTCGTTAGAAAACATCCTGAAGTCAAAAAACGTGGCAGAGAACTCAATATGTCTGATATCTATAACAATCCAGTGCTGCGGTTTCAGA**

**2848 cDNA**  **GTAGGATGGCTACTCGTTAGAAAACATCCTGAAGTCAAAAAACGTGGCAGAGAACTCAATATGTCTGATATCTATAACAATCCAGTGCTGCGGTTTCAGA**

**fam35 female gDNA**

**fam35-06 cDNA**  **GTAGGATGGCTACTCGTTAGAAAACATCCTGAAGTCAAAAAACGTGGCAGAGAACTCAATATGTCTGATATCTATAACAATCCAGTGCTGCGGTTTCAGA**

**fam35-06 gDNA**

**fam35-08 cDNA**  **GTAGGATGGCTACTCGTTAGAAAACATCCTGAAGTCAAAAAACGTGGCAGAGAACTCAATATGTCTGATATCTATAACAATCCAGTGCTGCGGTTTCAGA**

**fam35-08 gDNA**

**fam35-223 cDNA**  **GTAGGATGGCTACTCGTTAGAAAACATCCTGAAGTCAAAAAACGTGGCAGAGAACTCAATATGTCTGATATCTATAACAATCCAGTGCTGCGGTTTCAGA**

**fam35-223 gDNA**  **xxxxxxxxxxxxxxxxxxxxxxxxxxxxxxxxxxxxxxxxxxxxxxxxxxxxxxxxxxxxxxxxxxxxxxxxxxxxxxxxxxxxxxxxTGCGGTTTCAGA**

**fam46 male gDNA**

**fam46-114 gDNA**

**fam46-115 gDNA**  **xxxxxxxxxxxxxxxxxxxxxxxxxxxxxxxxxxxxxxxxxxxxxxxxxxxxxxxxxxxxxxxxCTGATATCTATAACAATCCAGTGCTGCGGTTTCAGA**

**fam46-116 gDNA**  **----------------------------------------------------------------CTGATATCTATAACAATCCAGTGCTGCGGTTTCAGA**

1010 1020 1030 1040 1050 1060 1070 1080 1090 1100

....|....|....|....|....|....|....|....|....|....|....|....|....|....|....|....|....|....|....|....|

**L allel gDNA**  **AAAA<GTAAGTATGCTAAACTGATTTTTTTTTAAGTAAATAATAAATATTAGATCAACATCAGAGGAATTATATACCCAGATTATGATAAAGAACATAAA**

**L allel cDNA**  **AAAA<-----------------------------------------------------------------------------------------------**

**H allel fam35-07 gDNA** **AAAA<GTAAGTGTG-AAAA-T-ATTTTTTATTA-------------------------------------------------------------------**

**H56 01A**  **AAAA<GTAAGTATGCTAAACTGATTTTTTTTTAAGTAAATAATAAATATTAGATCAACATCAGAGGAATTATATACCCAGATTATGATAAAGAACATAAA**

**H56 01B**  **AAAA<GTAAGTATGCTAAACTGATTTTTTTTTAAGTAAATAATAAATATTAGATCAACATCAGAGGAATTATATACCCAGATTATGATAAAGAACATAAA**

**H56 01G**  **AAAA<GTAAGTATGCTAAACTGATTTTTTTTTAAGTAAATAATAAATATTAGATCAACATCAGAGGAATTATATACCCAGATTATGATAAAGAACATAAA**

**H56 04A**

**H56 04C**

**H56 04D**

**H56 07A**

**H56 07B**

**H56 07C**

**H56 07F**

**H56 07G**

**H56 10A**

**H56 10F**

**H56 10H**

**H56 12A**

**H56 12B**

**H56 12C**

**H56 12D**

**9173 gDNA**

**9183 gDNA**

**9185 gDNA**  **AAAA<GTAAGTATGCTAAACTGATTTTTTTTTAAGTAAATAATAAATATTAGATCAACATCAGAGGAATTATATACCCAGATTATGATAAAGAACATAAA**

**10103 gDNA**  **AAAA<GTAAGTATGCTAAACTGATTTTTTTTTAAGTAAATAATAAATATTAGATCAACATCAGAGGAATTATATACCCAGATTATGATAAAGAACATAAA**

**1082 gDNA**  **AAAA<GTAAGTATGCTAAACTGATTTTTTTTTAAGTAAATAATAAATATTAGATCAACATCAGAGGAATTATATACCCAGATTATGATAAAGAACATAAA**

**1351 gDNA**  **AAAA<GTAAGTATGCTAAACTGATTTTTTTTTAAGTAAATAATAAATATTAGATCAACATCAGAGGAATTATATACCCAGATTATGATAAAGAACATAAA**

**1364 gDNA**  **AAAA<GTAAGTATGCTAAACTGATTTTTTTTTAAGTAAATAATAAATATTAGATCAACATCAGAGGAATTATATACCCAGATTATGATAAAGAACATAAA**

**14142 gDNA**  **AAAA<GTAAGTATGCTAAACTGATTTTTTTTTAAGTAAATAATAAATATTAGATCAACATCAGAGGAATTATATACCCAGATTATGATAAAGAACATAAA**

**1434 gDNA**  **AAAA<GTAAGTATGCTAAACTGATTTTTTTTTAAGTAAATAATAAATATTAGATCAACATCAGAGGAATTATATACCCAGATTATGATAAAGAACATAAA**

**15109 gDNA**  **AAAA<GTAAGTATGCTAAACTGATTTTTTTTTAAGTAAATAATAAATATTAGATCAACATCAGAGGAATTATATACCCAGATTATGATAAAGAACATAAA**

**15282 gDNA**

**1560 gDNA**

**1581 gDNA**

**1582 gDNA**  **AAAA<GTAAGTATGCTAAACTGATTTTTTTTTAAGTAAATAATAAATATTAGATCAACATCAGAGGAATTATATACCCAGATTATGATAAAGAACATAAA**

**2816 cDNA**  **AAAA<-----------------------------------------------------------------------------------------------**

**2844 cDNA**  **AAAA<-----------------------------------------------------------------------------------------------**

**2848 cDNA**  **AAAA<-----------------------------------------------------------------------------------------------**

**fam35 female gDNA**

**fam35-06 cDNA**  **AAAA<-----------------------------------------------------------------------------------------------**

**fam35-06 gDNA**

**fam35-08 cDNA**  **AAAA<-----------------------------------------------------------------------------------------------**

**fam35-08 gDNA**

**fam35-223 cDNA**  **AAAA<-----------------------------------------------------------------------------------------------**

**fam35-223 gDNA**  **AAAA<GTAAGTATGCTAAACTGATTTTTTTTTAAGTAAATAATAAATATTAGATCAACATCAGAGGAATTATATACCCAGATTATGATAAAGAACATAAA**

**fam46 male gDNA**

**fam46-114 gDNA**

**fam46-115 gDNA**  **AAAA<GTAAGTATGCTAAACTGATTTTTTTTTAAGTAAATAATAAATATTAGATCAACATCAGAGGAATTATATACCCAGATTATGATAAAGAACATAAA**

**fam46-116 gDNA**  **AAAA<GTAAGTATGCTAAACTGATTTTTTTTTAAGTAAATAATAAATATTAGATCAACATCAGAGGAATTATATACCCAGATTATGATAAAGAACATAAA**

1110 1120 1130 1140 1150 1160 1170 1180 1190 1200

....|....|....|....|....|....|....|....|....|....|....|....|....|....|....|....|....|....|....|....|

**L allel gDNA**  **AAGGATCGGTTTTCCCAATTTTTGATTAGGAAAGTACTAAATCAAAGAAAACTAAACGCCAATTTCACAGGTTCTAGATAAGTTCTAAATAGATATCTGA**

**L allel cDNA**  **----------------------------------------------------------------------------------------------------**

**H allel fam35-07 gDNA** **----------------------------------------------------------------------------------------------------**

**H56 01A**  **AAGGATCGGTTTTCCCAATTTTTGATTAGGAAAGTACTAAATCAAAGAAAACTAAACGCCAATTTCACAGGTTCTAGATAAGTTCTAAATAGATATCTGA**

**H56 01B**  **AAGGATCGGTTTTCCCAATTTTTGATTAGGAAAGTACTAAATCAAAGAAAACTAAACGCCAATTTCACAGGTTCTAGATAAGTTCTAAATAGATATCTGA**

**H56 01G**  **AAGGATCGGTTTTCCCAATTTTTGATTAGGAAAGTACTAAATCAAAGAAAACTAAACGCCAATTTCACAGGTTCTAGATAAGTTCTAAATAGATATCTGA**

**H56 04A**

**H56 04C**

**H56 04D**

**H56 07A**

**H56 07B**

**H56 07C**

**H56 07F**

**H56 07G**

**H56 10A**

**H56 10F**

**H56 10H**

**H56 12A**

**H56 12B**

**H56 12C**

**H56 12D**

**9173 gDNA**

**9183 gDNA**

**9185 gDNA**  **AAGGATCGGTTTTCCCAATTTTTGATTAGGAAAGTACTAAATCAAAGAAAACTAAACGCCAATTTCACAGGTTCTAGATAAGTTCTAAATAGATATCTGA**

**10103 gDNA**  **AAGGATCGGTTTTCCCAATTTTTGATTAGGAAAGTACTAAATCAAAGAAAACTAAACGCCAATTTCACAGGTTCTAGATAAGTTCTAAATAGATATCTGA**

**1082 gDNA**  **AAGGATCGGTTTTCCCAATTTTTGATTAGGAAAGTACTAAATCAAAGAAAACTAAACGCCAATTTCACAGGTTCTAGATAAGTTCTAAATAGATATCTGA**

**1351 gDNA**  **AAGGATCGGTTTTCCCAATTTTTGATTAGGAAAGTACTAAATCAAAGAAAACTAAACGCCAATTTCACAGGTTCTAGATAAGTTCTAAATAGATATCTGA**

**1364 gDNA**  **AAGGATCGGTTTTCCCAATTTTTGATTAGGAAAGTACTAAATCAAAGAAAACTAAACGCCAATTTCACAGGTTCTAGATAAGTTCTAAATAGATATCTGA**

**14142 gDNA**  **AAGGATCGGTTTTCCCAATTTTTGATTAGGAAAGTACTAAATCAAAGAAAACTAAACGCCAATTTCACAGGTTCTAGATAAGTTCTAAATAGATATCTGA**

**1434 gDNA**  **AAGGATCGGTTTTCCCAATTTTTGATTAGGAAAGTACTAAATCAAAGAAAACTAAACGCCAATTTCACAGGTTCTAGATAAGTTCTAAATAGATATCTGA**

**15109 gDNA**  **AAGGATCGGTTTTCCCAATTTTTGATTAGGAAAGTACTAAATCAAAGAAAACTAAACGCCAATTTCACAGGTTCTAGATAAGTTCTAAATAGATATCTGA**

**15282 gDNA**

**1560 gDNA**

**1581 gDNA**

**1582 gDNA**  **AAGGATCGGTTTTCCCAATTTTTGATTAGGAAAGTACTAAATCAAAGAAAACTAAACGCCAATTTCACAGGTTCTAGATAAGTTCTAAATAGATATCTGA**

**2816 cDNA**  **----------------------------------------------------------------------------------------------------**

**2844 cDNA**  **----------------------------------------------------------------------------------------------------**

**2848 cDNA**  **----------------------------------------------------------------------------------------------------**

**fam35 female gDNA**

**fam35-06 cDNA**  **----------------------------------------------------------------------------------------------------**

**fam35-06 gDNA**

**fam35-08 cDNA**  **----------------------------------------------------------------------------------------------------**

**fam35-08 gDNA**

**fam35-223 cDNA**  **----------------------------------------------------------------------------------------------------**

**fam35-223 gDNA**  **AAGGATCGGTTTTCCCAATTTTTGATTAGGAAAGTACTAAATCAAAGAAAACTAAACGCCAATTTCACAGGTTCTAGATAAGTTCTAAATAGATATCTGA**

**fam46 male gDNA**

**fam46-114 gDNA**

**fam46-115 gDNA**  **AAGGATCGGTTTTCCCAATTTTTGATTAGGAAAGTACTAAATCAAAGAAAACTAAACGCCAATTTCACAGGTTCTAGATAAGTTCTAAATAGATATCTGA**

**fam46-116 gDNA**  **AAGGATCGGTTTTCCCAATTTTTGATTAGGAAAGTACTAAATCAAAGAAAACTAAACGCCAATTTCACAGGTTCTAGATAAGTTCTAAATAGATATCTGA**

1210 1220 1230 1240 1250 1260 1270 1280 1290 1300

....|....|....|....|....|....|....|....|....|....|....|....|....|....|....|....|....|....|....|....|

**L allel gDNA**  **CAGATCATGTCATAGAAAAGTTATAAAAATTAAGAATATTTCTATGGCATCTCCCATAACATGATCTGTCAGATAGCTATCTGGGACTCTACCTGGAACT**

**L allel cDNA**  **----------------------------------------------------------------------------------------------------**

**H allel fam35-07 gDNA** **----------------------------------------------------------------------------------------------------**

**H56 01A**  **CAGATCATGTCATAGAAAAGTTATAAAAATTAAGAATATTTCTATGGCATCTCCCATAACATGATCTGTCAGATAGCTATCTGGGACTCTACCTGGAACT**

**H56 01B**  **CAGATCATGTCATAGAAAAGTTATAAAAATTAAGAATATTTCTATGGCATCTCCCATAACATGATCTGTCAGATAGCTATCTGGGACTCTACCTGGAACT**

**H56 01G**  **CAGATCATGTCATAGAAAAGTTATAAAAATTAAGAATATTTCTATGGCATCTCCCATAACATGATCTGTCAGATAGCTATCTGGGACTCTACCTGGAACT**

**H56 04A**

**H56 04C**

**H56 04D**

**H56 07A**

**H56 07B**

**H56 07C**

**H56 07F**

**H56 07G**

**H56 10A**

**H56 10F**

**H56 10H**

**H56 12A**

**H56 12B**

**H56 12C**

**H56 12D**

**9173 gDNA**

**9183 gDNA**

**9185 gDNA**  **CAGATCATGTCATAGAAAAGTTATAAAAATTAAGAATATTTCTATGGCATCTCCCATAACATGATCTGTCAGATAGCTATCTGGGACTCTACCTGGAACT**

**10103 gDNA**  **CAGATCATGTCATAGAAAAGTTATAAAAATTAAGAATATTTCTATGGCATCTCCCATAACATGATCTGTCAGATAGCTATCTGGGACTCTACCTGGAACT**

**1082 gDNA**  **CAGATCATGTCATAGAAAAGTTATAAAAATTAAGAATATTTCTATGGCATCTCCCATAACATGATCTGTCAGATAGCTATCTGGGACTCTACCTGGAACT**

**1351 gDNA**  **CAGATCATGTCATAGAAAAGTTATAAAAATTAAGAATATTTCTATGGCATCTCCCATAACATGATCTGTCAGATAGCTATCTGGGACTCTACCTGGAACT**

**1364 gDNA**  **CAGATCATGTCATAGAAAAGTTATAAAAATTAAGAATATTTCTATGGCATCTCCCATAACATGATCTGTCAGATAGCTATCTGGGACTCTACCTGGAACT**

**14142 gDNA**  **CAGATCATGTCATAGAAAAGTTATAAAAATTAAGAATATTTCTATGGCATCTCCCATAACATGATCTGTCAGATAGCTATCTGGGACTCTACCTGGAACT**

**1434 gDNA**  **CAGATCATGTCATAGAAAAGTTATAAAAATTAAGAATATTTCTATGGCATCTCCCATAACATGATCTGTCAGATAGCTATCTGGGACTCTACCTGGAACT**

**15109 gDNA**  **CAGATCATGTCATAGAAAAGTTATAAAAATTAAGAATATTTCTATGGCATCTCCCATAACATGATCTGTCAGATAGCTATCTGGGACTCTACCTGGAACT**

**15282 gDNA**

**1560 gDNA**

**1581 gDNA**

**1582 gDNA**  **CAGATCATGTCATAGAAAAGTTATAAAAATTAAGAATATTTCTATGGCATCTCCCATAACATGATCTGTCAGATAGCTATCTGGGACTCTACCTGGAACT**

**2816 cDNA**  **----------------------------------------------------------------------------------------------------**

**2844 cDNA**  **----------------------------------------------------------------------------------------------------**

**2848 cDNA**  **----------------------------------------------------------------------------------------------------**

**fam35 female gDNA**

**fam35-06 cDNA**  **----------------------------------------------------------------------------------------------------**

**fam35-06 gDNA**

**fam35-08 cDNA**  **----------------------------------------------------------------------------------------------------**

**fam35-08 gDNA**

**fam35-223 cDNA**  **----------------------------------------------------------------------------------------------------**

**fam35-223 gDNA**  **CAGATCATGTCATAGAAAAGTTATAAAAATTAAGAATATTTCTATGGCATCTCCCATAACATGATCTGTCAGATAGCTATCTGGGACTCTACCTGGAACT**

**fam46 male gDNA**

**fam46-114 gDNA**

**fam46-115 gDNA**  **CAGATCATGTCATAGAAAAGTTATAAAAATTAAGAATATTTCTATGGCATCTCCCATAACATGATCTGTCAGATAGCTATCTGGGACTCTACCTGGAACT**

**fam46-116 gDNA**  **CAGATCATGTCATAGAAAAGTTATAAAAATTAAGAATATTTCTATGGCATCTCCCATAACATGATCTGTCAGATAGCTATCTGGGACTCTACCTGGAACT**

1310 1320 1330 1340 1350 1360 1370 1380 1390 1400

....|....|....|....|....|....|....|....|....|....|....|....|....|....|....|....|....|....|....|....|

**L allel gDNA**  **TGTGAAACTGGCCGTAAATCTTGTTCTAAACTCAATACGCAGAACATAGATATACATAAATTGTGACAAAACTTTCGTTTTTCT-GCAG>ATACGCCGTA**

**L allel cDNA**  **----------------------------------------------------------------------------------------->ATACGCCGTA**

**H allel fam35-07 gDNA** **----------------GATATTTTTTGGACATC----GCAAATACATATATATTCAGAAATTGTGTAAAAACCTCCGTTTTCCTTGCAG>GTACGCCATA**

**H56 01A**  **TGTGAAACTGGCCGTAAATCTTGTTCTAAACTCAATACGCAGAACATAGATATACATAAATTGTGACAAAACTTTCGTTTTTCT-GCAG>ATACGCCGTA**

**H56 01B**  **TGTGAAACTGGCCGTAAATCTTGTTCTAAACTCAATACGCAGAACATAGATATACATAAATTGTGACAAAACTTTCGTTTTTCT-GCAG>ATACGCCGTA**

**H56 01G**  **TGTGAAACTGGCCGTAAATCTTGTTCTAAACTCAATACGCAGAACATAGATATACATAAATTGTGACAAAACTTTCGTTTTTCT-GCAG>ATACGCCGTA**

**H56 04A**

**H56 04C**

**H56 04D**

**H56 07A**

**H56 07B**

**H56 07C**

**H56 07F**

**H56 07G**

**H56 10A**

**H56 10F**

**H56 10H**

**H56 12A**

**H56 12B**

**H56 12C**

**H56 12D**

**9173 gDNA**

**9183 gDNA**

**9185 gDNA**  **TGTGAAACTGGCCGTAAATCTTGTTCTAAACTCAATACGCAGAACATAGATATACATAAATTGTGACAAAACTTTCGTTTTTCT-GCAG>ATACGCCGTA**

**10103 gDNA**  **TGTGAAACTGGCCGTAAATCTTGTTCTAAACTCAATACGCAGAACATAGATATACATAAATTGTGACAAAACTTTCGTTTTTCT-GCAG>ATACGCCGTA**

**1082 gDNA**  **TGTGAAACTGGCCGTAAATCTTGTTCTAAACTCAATACGCAGAACATAGATATACATAAATTGTGACAAAACTTTCGTTTTTCT-GCAG>ATACGCCGTA**

**1351 gDNA**  **TGTGAAACTGGCCGTAAATCTTGTTCTAAACTCAATACGCAGAACATAGATATACATAAATTGTGACAAAACTTTCGTTTTTCT-GCAG>ATACGCCGTA**

**1364 gDNA**  **TGTGAAACTGGCCGTAAATCTTGTTCTAAACTCAATACGCAGAACATAGATATACATAAATTGTGACAAAACTTTCGTTTTTCT-GCAG>ATACGCCGTA**

**14142 gDNA**  **TGTGAAACTGGCCGTAAATCTTGTTCTAAACTCAATACGCAGAACATAGATATACATAAATTGTGACAAAACTTTCGTTTTTCT-GCAG>ATACGCCGTA**

**1434 gDNA**  **TGTGAAACTGGCCGTAAATCTTGTTCTAAACTCAATACGCAGAACATAGATATACATAAATTGTGACAAAACTTTCGTTTTTCT-GCAG>ATACGCCGTA**

**15109 gDNA**  **TGTGAAACTGGCCGTAAATCTTGTTCTAAACTCAATACGCAGAACATAGATATACATAAATTGTGACAAAACTTTCGTTTTTCT-GCAG>ATACGCCGTA**

**15282 gDNA**

**1560 gDNA**

**1581 gDNA**

**1582 gDNA**  **TGTGAAACTGGCCGTAAATCTTGTTCTAAACTCAATACGCAGAACATAGATATACATAAATTGTGACAAAACTTTCGTTTTTCT-GCAG>ATACGCCGTA**

**2816 cDNA**  **----------------------------------------------------------------------------------------->ATACGCCGTA**

**2844 cDNA**  **----------------------------------------------------------------------------------------->ATACGCCGTA**

**2848 cDNA**  **----------------------------------------------------------------------------------------->ATACGCCGTA**

**fam35 female gDNA**

**fam35-06 cDNA**  **----------------------------------------------------------------------------------------->ATACGCCGTA**

**fam35-06 gDNA**

**fam35-08 cDNA**  **----------------------------------------------------------------------------------------->ATACGCCGTA**

**fam35-08 gDNA**

**fam35-223 cDNA**  **----------------------------------------------------------------------------------------->ATACGCCGTA**

**fam35-223 gDNA**  **TGTGAAACTGGCCGTAAATCTTGTTCTAAACTCAATACGCAGAACATAGATATACATAAATTGTGACAAAACTTTCGTTTTTCT-GCAG>ATACGCCGTA**

**fam46 male gDNA**

**fam46-114 gDNA**

**fam46-115 gDNA**  **TGTGAAACTGGCCGTAAATCTTGTTCTAAACTCAATACGCAGAACATAGATATACATAAATTGTGACAAAACTTTCGTTTTTCT-GCAG>ATACGCCGTA**

**fam46-116 gDNA**  **TGTGAAACTGGCCGTAAATCTTGTTCTAAACTCAATACGCAGAACATAGATATACATAAATTGTGACAAAACTTTCGTTTTTCT-GCAG>ATACGCCGTA**

1410 1420 1430 1440 1450 1460 1470 1480 1490 1500

....|....|....|....|....|....|....|....|....|....|....|....|....|....|....|....|....|....|....|....|

**L allel gDNA**  **CCCTTCATTGGAGCTGTTTGTTTCGTCTTACCTACATTAATACCCGTCTACTGCTGGGGAGAAACCTGGTCCAATGCCTGGCACATCACCATGCTTCGTT**

**L allel cDNA**  **CCCTTCATTGGAGCTGTTTGTTTCGTCTTACCTACATTAATACCCGTCTACTGCTGGGGAGAAACCTGGTCCAATGCCTGGCACATCACCATGCTTCGTT**

**H allel fam35-07 gDNA** **CCCTTCATCGGAGCTGTTTGTTTCGTCTTACCTACATTGATACCCGTCTACTGCTGGGGAGAAACCTGGTCCAATGCCTGGCACATCACCATGCTTCGTT**

**H56 01A**  **CCCTTCATTGGAGCTGTTTGTTTCGTCTTACCTACATTAATACCCGTCTACTGCTGGGGAGAAACCTGGTCCAATGCCTGGCACATCACCATGCTTCGTT**

**H56 01B**  **CCCTTCATTGGAGCTGTTTGTTTCGTCTTACCTACATTAATACCCGTCTACTGCTGGGGAGAAACCTGGTCCAATGCCTGGCACATCACCATGCTTCGTT**

**H56 01G**  **CCCTTCATTGGAGCTGTTTGTTTCGTCTTACCTACATTAATACCCGTCTACTGCTGGGGAGAAACCTGGTCCAATGCCTGGCACATCACCATGCTTCGTT**

**H56 04A**

**H56 04C**

**H56 04D**

**H56 07A**

**H56 07B**

**H56 07C**

**H56 07F**

**H56 07G**

**H56 10A**

**H56 10F**

**H56 10H**

**H56 12A**

**H56 12B**

**H56 12C**

**H56 12D**

**9173 gDNA**

**9183 gDNA**

**9185 gDNA**  **CCCTTCATTGGAGCTGTTTGTTTCGTCTTACCTACATTAATACCCGTCTACTGCTGGGGAGAAACCTGGTCCAATGCCTGGCACATCACCATGCTTCGTT**

**10103 gDNA**  **CCCTTCATTGGAGCTGTTTGTTTCGTCTTACCTACATTAATACCCGTCTACTGCTGGGGAGAAACCTGGTCCAATGCCTGGCACATCACCATGCTTCGTT**

**1082 gDNA**  **CCCTTCATTGGAGCTGTTTGTTTCGTCTTACCTACATTAATACCCGTCTACTGCTGGGGAGAAACCTGGTCCAATGCCTGGCACATCACCATGCTTCGTT**

**1351 gDNA**  **CCCTTCATTGGAGCTGTTTGTTTCGTCTTACCTACATTAATACCCGTCTACTGCTGGGGAGAAACCTGGTCCAATGCCTGGCACATCACCATGCTTCGTT**

**1364 gDNA**  **CCCTTCATTGGAGCTGTTTGTTTCGTCTTACCTACATTAATACCCGTCTACTGCTGGGGAGAAACCTGGTCCAATGCCTGGCACATCACCATGCTTCGTT**

**14142 gDNA**  **CCCTTCATTGGAGCTGTTTGTTTCGTCTTACCTACATTAATACCCGTCTACTGCTGGGGAGAAACCTGGTCCAATGCCTGGCACATCACCATGCTTCGTT**

**1434 gDNA**  **CCCTTCATTGGAGCTGTTTGTTTCGTCTTACCTACATTAATACCCGTCTACTGCTGGGGAGAAACCTGGTCCAATGCCTGGCACATCACCATGCTTCGTT**

**15109 gDNA**  **CCCTTCATTGGAGCTGTTTGTTTCGTCTTACCTACATTAATACCCGTCTACTGCTGGGGAGAAACCTGGTCCAATGCCTGGCACATCACCATGCTTCGTT**

**15282 gDNA**

**1560 gDNA**

**1581 gDNA**

**1582 gDNA**  **CCCTTCATTGGAGCTGTTTGTTTCGTCTTACCTACATTAATACCCGTCTACTGCTGGGGAGAAACCTGGTCCAATGCCTGGCACATCACCATGCTTCGTT**

**2816 cDNA**  **CCCTTCATTGGAGCTGTTTGTTTCGTCTTACCTACATTAATACCCGTCTACTGCTGGGGAGAAACCTGGTCCAATGCCTGGCACATCACCATGCTTCGTT**

**2844 cDNA**  **CCCTTCATTGGAGCTGTTTGTTTCGTCTTACCTACATTAATACCCGTCTACTGCTGGGGAGAAACCTGGTCCAATGCCTGGCACATCACCATGCTTCGTT**

**2848 cDNA**  **CCCTTCATTGGAGCTGTTTGTTTCGTCTTACCTACATTAATACCCGTCTACTGCTGGGGAGAAACCTGGTCCAATGCCTGGCACATCACCATGCTTCGTT**

**fam35 female gDNA**

**fam35-06 cDNA**  **CCCTTCATTGGAGCTGTTTGTTTCGTCTTACCTACATTAATACCCGTCTACTGCTGGGGAGAAACCTGGTCCAATGCCTGGCACATCACCATGCTTCGTT**

**fam35-06 gDNA**

**fam35-08 cDNA**  **CCCTTCATTGGAGCTGTTTGTTTCGTCTTACCTACATTAATACCCGTCTACTGCTGGGGAGAAACCTGGTCCAATGCCTGGCACATCACCATGCTTCGTT**

**fam35-08 gDNA**

**fam35-223 cDNA**  **CCCTTCATTGGAGCTGTTTGTTTCGTCTTACCTACATTAATACCCGTCTACTGCTGGGGAGAAACCTGGTCCAATGCCTGGCACATCACCATGCTTCGTT**

**fam35-223 gDNA**  **CCCTTCATTGGAGCTGTTTGTTTCGTCTTACCTACATTAATACCCGTCTACTGCTGGGGAGAAACCTGGTCCAATGCCTGGCACATCACCATGCTTCGTT**

**fam46 male gDNA**

**fam46-114 gDNA**

**fam46-115 gDNA**  **CCCTTCATTGGAGCTGTTTGTTTCGTCTTACCTACATTAATACCCGTCTACTGCTGGGGAGAAACCTGGTCCAATGCCTGGCACATCACCATGCTTCGTT**

**fam46-116 gDNA**  **CCCTTCATTGGAGCTGTTTGTTTCGTCTTACCTACATTAATACCCGTCTACTGCTGGGGAGAAACCTGGTCCAATGCCTGGCACATCACCATGCTTCGTT**

1510 1520 1530 1540 1550 1560 1570 1580 1590 1600

....|....|....|....|....|....|....|....|....|....|....|....|....|....|....|....|....|....|....|....|

**L allel gDNA**  **ACATCATGAACCTCAACGTCACGTTTTTGGTGAACAGCGCCGCTCACATATGGGGTTACAAGCCTTATGACGCGAAAATATTACCTGTCCAAAATGTAGC**

**L allel cDNA**  **ACATCATGAACCTCAACGTCACGTTTTTGGTGAACAGCGCCGCTCACATATGGGGTTACAAGCCTTATGACGCGAAAATATTACCTGTCCAAAATGTAGC**

**H allel fam35-07 gDNA** **ACATCATGAACCTCAACGTCACGTTTTTGGTGAACAGCGCCGCTCACATATGGGGCTACAAGCCTTATGACGCGAAAATATTACCTGTCCAAAACGTAGC**

**H56 01A**  **ACATCATGAACCTCAACGTCACGTTTTTGGTGAACAGCGCCGCTCACATATGGGGTTACAAGCCTTATGACGCGAAAATATTACCTGTCCAAAATGTAGC**

**H56 01B**  **ACATCATGAACCTCAACGTCACGTTTTTGGTGAACAGCGCCGCTCACATATGGGGTTACAAGCCTTATGACGCGAAAATATTACCTGTCCAAAATGTAGC**

**H56 01G**  **ACATCATGAACCTCAACGTCACGTTTTTGGTGAACAGCGCCGCTCACATATGGGGTTACAAGCCTTATGACGCGAAAATATTACCTGTCCAAAATGTAGC**

**H56 04A**

**H56 04C**

**H56 04D**

**H56 07A**

**H56 07B**

**H56 07C**

**H56 07F**

**H56 07G**

**H56 10A**

**H56 10F**

**H56 10H**

**H56 12A**

**H56 12B**

**H56 12C**

**H56 12D**

**9173 gDNA**

**9183 gDNA**

**9185 gDNA**  **ACATCATGAACCTCAACGTCACGTTTTTGGTGAACAGCGCCGCTCACATATGGGGTTACAAGCCTTATGACGCGAAAATATTACCTGTCCAAAATGTAGC**

**10103 gDNA**  **ACATCATGAACCTCAACGTCACGTTTTTGGTGAACAGCGCCGCTCACATATGGGGTTACAAGCCTTATGACGCGAAAATATTACCTGTCCAAAATGTAGC**

**1082 gDNA**  **ACATCATGAACCTCAACGTCACGTTTTTGGTGAACAGCGCCGCTCACATATGGGGTTACAAGCCTTATGACGCGAAAATATTACCTGTCCAAAATGTAGC**

**1351 gDNA**  **ACATCATGAACCTCAACGTCACGTTTTTGGTGAACAGCGCCGCTCACATATGGGGTTACAAGCCTTATGACGCGAAAATATTACCTGTCCAAAATGTAGC**

**1364 gDNA**  **ACATCATGAACCTCAACGTCACGTTTTTGGTGAACAGCGCCGCTCACATATGGGGTTACAAGCCTTATGACGCGAAAATATTACCTGTCCAAAATGTAGC**

**14142 gDNA**  **ACATCATGAACCTCAACGTCACGTTTTTGGTGAACAGCGCCGCTCACATATGGGGTTACAAGCCTTATGACGCGAAAATATTACCTGTCCAAAATGTAGC**

**1434 gDNA**  **ACATCATGAACCTCAACGTCACGTTTTTGGTGAACAGCGCCGCTCACATATGGGGTTACAAGCCTTATGACGCGAAAATATTACCTGTCCAAAATGTAGC**

**15109 gDNA**  **ACATCATGAACCTCAACGTCACGTTTTTGGTGAACAGCGCCGCTCACATATGGGGTTACAAGCCTTATGACGCGAAAATATTACCTGTCCAAAATGTAGC**

**15282 gDNA**

**1560 gDNA**

**1581 gDNA**

**1582 gDNA**  **ACATCATGAACCTCAACGTCACGTTTTTGGTGAACAGCGCCGCTCACATATGGGGTTACAAGCCTTATGACGCGAAAATATTACCTGTCCAAAATGTAGC**

**2816 cDNA**  **ACATCATGAACCTCAACGTCACGTTTTTGGTGAACAGCGCCGCTCACATATGGGGTTACAAGCCTTATGACGCGAAAATATTACCTGTCCAAAATGTAGC**

**2844 cDNA**  **ACATCATGAACCTCAACGTCACGTTTTTGGTGAACAGCGCCGCTCACATATGGGGTTACAAGCCTTATGACGCGAAAATATTACCTGTCCAAAATGTAGC**

**2848 cDNA**  **ACATCATGAACCTCAACGTCACGTTTTTGGTGAACAGCGCCGCTCACATATGGGGTTACAAGCCTTATGACGCGAAAATATTACCTGTCCAAAATGTAGC**

**fam35 female gDNA**

**fam35-06 cDNA**  **ACATCATGAACCTCAACGTCACGTTTTTGGTGAACAGCGCCGCTCACATATGGGGTTACAAGCCTTATGACGCGAAAATATTACCTGTCCAAAATGTAGC**

**fam35-06 gDNA**

**fam35-08 cDNA**  **ACATCATGAACCTCAACGTCACGTTTTTGGTGAACAGCGCCGCTCACATATGGGGTTACAAGCCTTATGACGCGAAAATATTACCTGTCCAAAATGTAGC**

**fam35-08 gDNA**

**fam35-223 cDNA**  **ACATCATGAACCTCAACGTCACGTTTTTGGTGAACAGCGCCGCTCACATATGGGGTTACAAGCCTTATGACGCGAAAATATTACCTGTCCAAAATGTAGC**

**fam35-223 gDNA**  **ACATCATGAACCTCAACGTCACGTTTTTGGTGAACAGCGCCGCTCACATATGGGGTTACAAGCCTTATGACGCGAAAATATTACCTGTCCAAAATGTAGC**

**fam46 male gDNA**

**fam46-114 gDNA**

**fam46-115 gDNA**  **ACATCATGAACCTCAACGTCACGTTTTTGGTGAACAGCGCCGCTCACATATGGGGTTACAAGCCTTATGACGCGAAAATATTACCTGTCCAAAATGTAGC**

**fam46-116 gDNA**  **ACATCATGAACCTCAACGTCACGTTTTTGGTGAACAGCGCCGCTCACATATGGGGTTACAAGCCTTATGACGCGAAAATATTACCTGTCCAAAATGTAGC**

1610 1620 1630 1640 1650 1660 1670 1680 1690 1700

....|....|....|....|....|....|....|....|....|....|....|....|....|....|....|....|....|....|....|....|

**L allel gDNA**  **TGTGTCCGTCGCGACTGGTGGAGAAGGTTTTCATAATTATCACCACGTGTTCCCTTGGGATTATCGAGCAGCGGAACTCGGGAACAATAGCCTCAATCTG**

**L allel cDNA**  **TGTGTCCGTCGCGACTGGTGGAGAAGGTTTTCATAATTATCACCACGTGTTCCCTTGGGATTATCGAGCAGCGGAACTCGGGAACAATAGCCTCAATCTG**

**H allel fam35-07 gDNA** **TGTGTCCGTCGCGACTGGTGGAGAAGGTTTTCATAATTATCACCACGTGTTCCCTTGGGATTATCGAGCAGCGGAACTTGGGAACAATAGCCTCAATCTG**

**H56 01A**  **TGTGTCCGTCGCGACTGGTGGAGAAGGTTTTCATAATTATCACCACGTGTTCCCTTGGGATTATCGAGCAGCGGAACTCGGGAACAATAGCCTCAATCTG**

**H56 01B**  **TGTGTCCGTCGCGACTGGTGGAGAAGGTTTTCATAATTATCACCACGTGTTCCCTTGGGATTATCGAGCAGCGGAACTCGGGAACAATAGCCTCAATCTG**

**H56 01G**  **TGTGTCCGTCGCGACTGGTGGAGAAGGTTTTCATAATTATCACCACGTGTTCCCTTGGGATTATCGAGCAGCGGAACTCGGGAACAATAGCCTCAATCTG**

**H56 04A**

**H56 04C**

**H56 04D**

**H56 07A**

**H56 07B**

**H56 07C**

**H56 07F**

**H56 07G**

**H56 10A**

**H56 10F**

**H56 10H**

**H56 12A**

**H56 12B**

**H56 12C**

**H56 12D**

**9173 gDNA**

**9183 gDNA**

**9185 gDNA**  **TGTGTCCGTCGCGACTGGTGGAGAAGGTTTTCATAATTATCACCACGTGTTCCCTTGGGATTATCGAGCAGCGGAACTCGGGAACAATAGCCTCAATCTG**

**10103 gDNA**  **TGTGTCCGTCGCGACTGGTGGAGAAGGTTTTCATAATTATCACCACGTGTTCCCTTGGGATTATCGAGCAGCGGAACTCGGGAACAATAGCCTCAATCTG**

**1082 gDNA**  **TGTGTCCGTCGCGACTGGTGGAGAAGGTTTTCATAATTATCACCACGTGTTCCCTTGGGATTATCGAGCAGCGGAACTCGGGAACAATAGCCTCAATCTG**

**1351 gDNA**  **TGTGTCCGTCGCGACTGGTGGAGAAGGTTTTCATAATTATCACCACGTGTTCCCTTGGGATTATCGAGCAGCGGAACTCGGGAACAATAGCCTCAATCTG**

**1364 gDNA**  **TGTGTCCGTCGCGACTGGTGGAGAAGGTTTTCATAATTATCACCACGTGTTCCCTTGGGATTATCGAGCAGCGGAACTCGGGAACAATAGCCTCAATCTG**

**14142 gDNA**  **TGTGTCCGTCGCGACTGGTGGAGAAGGTTTTCATAATTATCACCACGTGTTCCCTTGGGATTATCGAGCAGCGGAACTCGGGAACAATAGCCTCAATCTG**

**1434 gDNA**  **TGTGTCCGTCGCGACTGGTGGAGAAGGTTTTCATAATTATCACCACGTGTTCCCTTGGGATTATCGAGCAGCGGAACTCGGGAACAATAGCCTCAATCTG**

**15109 gDNA**  **TGTGTCCGTCGCGACTGGTGGAGAAGGTTTTCATAATTATCACCACGTGTTCCCTTGGGATTATCGAGCAGCGGAACTCGGGAACAATAGCCTCAATCTG**

**15282 gDNA**

**1560 gDNA**

**1581 gDNA**

**1582 gDNA**  **TGTGTCCGTCGCGACTGGTGGAGAAGGTTTTCATAATTATCACCACGTGTTCCCTTGGGATTATCGAGCAGCGGAACTCGGGAACAATAGCCTCAATCTG**

**2816 cDNA**  **TGTGTCCGTCGCGACTGGTGGAGAAGGTTTTCATAATTATCACCACGTGTTCCCTTGGGATTATCGAGCAGCGGAACTCGGGAACAATAGCCTCAATCTG**

**2844 cDNA**  **TGTGTCCGTCGCGACTGGTGGAGAAGGTTTTCATAATTATCACCACGTGTTCCCTTGGGATTATCGAGCAGCGGAACTCGGGAACAATAGCCTCAATCTG**

**2848 cDNA**  **TGTGTCCGTCGCGACTGGTGGAGAAGGTTTTCATAATTATCACCACGTGTTCCCTTGGGATTATCGAGCAGCGGAACTCGGGAACAATAGCCTCAATCTG**

**fam35 female gDNA**

**fam35-06 cDNA**  **TGTGTCCGTCGCGACTGGTGGAGAAGGTTTTCATAATTATCACCACGTGTTCCCTTGGGATTATCGAGCAGCGGAACTCGGGAACAATAGCCTCAATCTG**

**fam35-06 gDNA**

**fam35-08 cDNA**  **TGTGTCCGTCGCGACTGGTGGAGAAGGTTTTCATAATTATCACCACGTGTTCCCTTGGGATTATCGAGCAGCGGAACTCGGGAACAATAGCCTCAATCTG**

**fam35-08 gDNA**

**fam35-223 cDNA**  **TGTGTCCGTCGCGACTGGTGGAGAAGGTTTTCATAATTATCACCACGTGTTCCCTTGGGATTATCGAGCAGCGGAACTCGGGAACAATAGCCTCAATCTG**

**fam35-223 gDNA**  **TGTGTCCGTCGCGACTGGTGGAGAAGGTTTTCATAATTATCACCACGTGTTCCCTTGGGATTATCGAGCAGCGGAACTCGGGAACAATAGCCTCAATCTG**

**fam46 male gDNA**

**fam46-114 gDNA**

**fam46-115 gDNA**  **TGTGTCCGTCGCGACTGGTGGAGAAGGTTTTCATAATTATCACCACGTGTTCCCTTGGGATTATCGAGCAGCGGAACTCGGGAACAATAGCCTCAATCTG**

**fam46-116 gDNA**  **TGTGTCCGTCGCGACTGGTGGAGAAGGTTTTCATAATTATCACCACGTGTTCCCTTGGGATTATCGAGCAGCGGAACTCGGGAACAATAGCCTCAATCTG**

1710 1720 1730 1740 1750 1760 1770 1780 1790 1800

....|....|....|....|....|....|....|....|....|....|....|....|....|....|....|....|....|....|....|....|

**L allel gDNA**  **ACGACTAAATTCATAGATTTCTTCGCATGGATCGGATGGGCATATGACCTGAAGACGGTTTCGGAAGATATGATAAAACTAAGGACTAAACGCACTGGAG**

**L allel cDNA**  **ACGACTAAATTCATAGATTTCTTCGCATGGATCGGATGGGCATATGACCTGAAGACGGTTTCGGAAGATATGATAAAACTAAGGACTAAACGCACTGGAG**

**H allel fam35-07 gDNA** **ACGACTAAATTCATAGATTTCTTCGCATGGATCGGATGGGCATATGACCTGAAGACGGTTTCGGAAGATATGATAAAATTAAGGACTAAACGCACTGGAG**

**H56 01A**  **ACGACTAAATTCATAGATTTCTTCGCATGGATCGGATGGGCATATGACCTGAAGACGGTTTCGGAAGATATGATAAAACTAAGGACTAAACGCACTGGAG**

**H56 01B**  **ACGACTAAATTCATAGATTTCTTCGCATGGATCGGATGGGCATATGACCTGAAGACGGTTTCGGAAGATATGATAAAACTAAGGACTAAACGCACTGGAG**

**H56 01G**  **ACGACTAAATTCATAGATTTCTTCGCATGGATCGGATGGGCATATGACCTGAAGACGGTTTCGGAAGATATGATAAAACTAAGGACTAAACGCACTGGAG**

**H56 04A**

**H56 04C**

**H56 04D**

**H56 07A**

**H56 07B**

**H56 07C**

**H56 07F**

**H56 07G**

**H56 10A**

**H56 10F**

**H56 10H**

**H56 12A**

**H56 12B**

**H56 12C**

**H56 12D**

**9173 gDNA**

**9183 gDNA**

**9185 gDNA**  **ACGACTAAATTCATAGATTTCTTCGCATGGATCGGATGGGCATATGACCTGA**

**10103 gDNA**  **ACGACTAAATTCATAGATTTCTTCGCATGGATCGGATGGGCATATGACCTGA**

**1082 gDNA**  **ACGACTAAATTCATAGATTTCTTCGCATGGATCGGATGGGCATATGACCTGA**

**1351 gDNA**  **ACGACTAAATTCATAGATTTCTTCGCATGGATCGGATGGGCATATGACCTGA**

**1364 gDNA**  **ACGACTAAATTCATAGATTTCTTCGCATGGATCGGATGGGCATATGACCTGA**

**14142 gDNA**  **ACGACTAAATTCATAGATTTCTTCGCATGGATCGGATGGGCATATGACCTGA**

**1434 gDNA**  **ACGACTAAATTCATAGATTTCTTCGCATGGATCGGATGGGCATATGACCTGA**

**15109 gDNA**  **ACGACTAAATTCATAGATTTCTTCGCATGGATCGGATGGGCATATGACCTGA**

**15282 gDNA**

**1560 gDNA**

**1581 gDNA**

**1582 gDNA**  **ACGACTAAATTCATAGATTTCTTCGCATGGATCGGATGGGCATATGACCTGA**

**2816 cDNA**  **ACGACTAAATTCATAGATTTCTTCGCATGGATCGGATGGGCATATGACCTGAAGACGGTTTCGGAAGATATGATAAAACTAAGGACTAAACGCACTGGAG**

**2844 cDNA**  **ACGACTAAATTCATAGATTTCTTCGCATGGATCGGATGGGCATATGACCTGAAGACGGTTTCGGAAGATATGATAAAACTAAGGACTAAACGCACTGGAG**

**2848 cDNA**  **ACGACTAAATTCATAGATTTCTTCGCATGGATCGGATGGGCATATGACCTGAAGACGGTTTCGGAAGATATGATAAAACTAAGGACTAAACGCACTGGAG**

**fam35 female gDNA**

**fam35-06 cDNA**  **ACGACTAAATTCATAGATTTCTTCGCATGGATCGGATGGGCATATGACCTGAAGACGGTTTCGGAAGATATGATAAAACTAAGGACTAAACGCACTGGAG**

**fam35-06 gDNA**

**fam35-08 cDNA**  **ACGACTAAATTCATAGATTTCTTCGCATGGATCGGATGGGCATATGACCTGAAGACGGTTTCGGAAGATATGATAAAACTAAGGACTAAACGCACTGGAG**

**fam35-08 gDNA**

**fam35-223 cDNA**  **ACGACTAAATTCATAGATTTCTTCGCATGGATCGGATGGGCATATGACCTGAAGACGGTTTCGGAAGATATGATAAAACTAAGGACTAAACGCACTGGAG**

**fam35-223 gDNA**  **ACGACTAAATTCATAGATTTCTTCGCATGGATCGGATGGGCATATGACCTGA**

**fam46 male gDNA**

**fam46-114 gDNA**

**fam46-115 gDNA**  **ACGACTAAATTCATAGATTTCTTCGCATGGATCGGATGGGCATATGACCTGA**

**fam46-116 gDNA**  **ACGACTAAATTCATAGATTTCTTCGCATGGATCGGATGGGCATATGACCTGA**

1810 1820 1830 1840 1850 1860 1870 1880 1890 1900

....|....|....|....|....|....|....|....|....|....|....|....|....|....|....|....|....|....|....|....|

**L allel gDNA**  **ATGGCACGGATCTTTGGGGACACGAACAAAAATATGATGAAGTATTGGATGTAAAAGATAAATAAAGTTGAATGATGGTAGAGGTTGCAACAGTGATTTA**

**L allel cDNA**  **ATGGCACGGATCTTTGGGGACACGAACAAAAATATGATGAAGTATTGGATGTAAAAGATAAATAAAGTTGAATGATGGTAGAGGTTGCAACAGTGATTTA**

**H allel fam35-07 gDNA** **ATGGCACGGATCTTTGGGGACACGAACAAAAATATGATGAAGTATTGGATGTAAAAGATAAATAAAGTAGAATGATGGTAGAGGTTCTAACAGTGATTTA**

**H56 01A**  **ATGGCACGGATCTTTGGGGACACGAACAAAAATATGATGAAGTATTGGATGTAAAAGATAAATAAAGTTGAATGATGGTAGAGGTTGCAACAGTGATTTA**

**H56 01B**  **ATGGCACGGATCTTTGGGGACACGAACAAAAATATGATGAAGTATTGGATGTAAAAGATAAATAAAGTTGAATGATGGTAGAGGTTGCAACAGTGATTTA**

**H56 01G**  **ATGGCACGGATCTTTGGGGACACGAACAAAAATATGATGAAGTATTGGATGTAAAAGATAAATAAAGTTGAATGATGGTAGAGGTTGCAACAGTGATTTA**

**H56 04A**

**H56 04C**

**H56 04D**

**H56 07A**

**H56 07B**

**H56 07C**

**H56 07F**

**H56 07G**

**H56 10A**

**H56 10F**

**H56 10H**

**H56 12A**

**H56 12B**

**H56 12C**

**H56 12D**

**9173 gDNA**

**9183 gDNA**

**9185 gDNA**

**10103 gDNA**

**1082 gDNA**

**1351 gDNA**

**1364 gDNA**

**14142 gDNA**

**1434 gDNA**

**15109 gDNA**

**15282 gDNA**

**1560 gDNA**

**1581 gDNA**

**1582 gDNA**

**2816 cDNA**  **ATGGCACGGATCTTTGGGGACACGAACAAAAATATGATGAAGTATTGGATGTAAAAGATAAATAAAGTTGAATGATGGTAGAGGTTGCAACAGTGATTTA**

**2844 cDNA**  **ATGGCACGGATCTTTGGGGACACGAACAAAAATATGATGAAGTATTGGATGTAAAAGATAAATAAAGTTGAATGATGGTAGAGGTTGCAACAGTGATTTA**

**2848 cDNA**  **ATGGCACGGATCTTTGGGGACACGAACAAAAATATGATGAAGTATTGGATGTAAAAGATAAATAAAGTTGAATGATGGTAGAGGTTGCAACAGTGATTTA**

**fam35 female gDNA**

**fam35-06 cDNA**  **ATGGCACGGATCTTTGGGGACACGAACAAAAATATGATGAAGTATTGGATGTAAAAGATAAATAAAGTTGAATGATGGTAGAGGTTGCAACAGTGATTTA**

**fam35-06 gDNA**

**fam35-08 cDNA**  **ATGGCACGGATCTTTGGGGACACGAACAAAAATATGATGAAGTATTGGATGTAAAAGATAAATAAAGTTGAATGATGGTAGAGGTTGCAACAGTGATTTA**

**fam35-08 gDNA**

**fam35-223 cDNA**  **ATGGCACGGATCTTTGGGGACACGAACAAAAATATGATGAAGTATTGGATGTAAAAGATAAATAAAGTTGAATGATGGTAGAGGTTGCAACAGTGATTTA**

**fam35-223 gDNA**

**fam46 male gDNA**

**fam46-114 gDNA**

**fam46-115 gDNA**

**fam46-116 gDNA**

1910 1920 1930 1940 1950

....|....|....|....|....|....|....|....|....|....|....|

**L allel gDNA**  **TTAATTGTGATACATATTGTATTTGTATTTATTGTAGTTGCCAGTATGGTTCTGT**

**L allel cDNA**  **TTAATTGTGATACATATTGTATTTGTATTTATTGTAGTTGCCAGTATGGTTCTGT**

**H allel fam35-07 gDNA** **TTAATTGTGATACATATTATATTTGTATTTCTTGTAGTTGCCAGTATGGTTCTGT**

**H56 01A**  **TTAATTGTGATACATATTGTATTTGTATTTATTGTAGTTGCCAGTATGGTTCTGT**

**H56 01B**  **TTAATTGTGATACATATTGTATTTGTATTTATTGTAGTTGCCAGTATGGTTCTGT**

**H56 01G**  **TTAATTGTGATACATATTGTATTTGTATTTATTGTAGTTGCCAGTATGGTTCTGT**

**H56 04A**

**H56 04C**

**H56 04D**

**H56 07A**

**H56 07B**

**H56 07C**

**H56 07F**

**H56 07G**

**H56 10A**

**H56 10F**

**H56 10H**

**H56 12A**

**H56 12B**

**H56 12C**

**H56 12D**

**9173 gDNA**

**9183 gDNA**

**9185 gDNA**

**10103 gDNA**

**1082 gDNA**

**1351 gDNA**

**1364 gDNA**

**14142 gDNA**

**1434 gDNA**

**15109 gDNA**

**15282 gDNA**

**1560 gDNA**

**1581 gDNA**

**1582 gDNA**

**2816 cDNA**  **TTAATTGTGATACATATTGTATTTGTATTTATTGTAGTTGCCAGTATGGTTCTGT**

**2844 cDNA**  **TTAATTGTGATACATATTGTATTTGTATTTATTGTAGTTGCCAGTATGGTTCTGT**

**2848 cDNA**  **TTAATTGTGATACATATTGTATTTGTATTTATTGTAGTTGCCAGTATGGTTCTGT**

**fam35 female gDNA**

**fam35-06 cDNA**  **TTAATTGTGATACATATTGTATTTGTATTTATTGTAGTTGCCAGTATGGTTCTGT**

**fam35-06 gDNA**

**fam35-08 cDNA**  **TTAATTGTGATACATATTGTATTTGTATTTATTGTAGTTGCCAGTATGGTTCTGT**

**fam35-08 gDNA**

**fam35-223 cDNA**  **TTAATTGTGATACATATTGTATTTGTATTTATTGTAGTTGCCAGTATGGTTCTGT**

**fam35-223 gDNA**

**fam46 male gDNA**

**fam46-114 gDNA**

**fam46-115 gDNA**

**fam46-116 gDNA**
