# Supplementary material for: Within-population variability in a moth sex pheromone blend, part 2: selection towards fixation
Source: R Soc Open Sci. 2019 Mar 13;6(3):182050. doi: 10.1098/rsos.182050 (PMC6458377; doi:10.1098/rsos.182050)
Supplement: Suppl. File 4 [file rsos182050supp5.docx]

Supplementary File 4 to Groot AT, van Wijk M, Villacis-Perez E, Kuperus P, Schöfl G, van Veldhuizen D, Heckel D. Within-population variability in a moth sex pheromone blend, part 2: Selection towards fixation. Royal Society Open Science.

Aligned nucleotide sequences of the exons of LPAQ delta-11-desaturase of field-collected females

10 20 30 40 50 60 70 80 90 100

....|....|....|....|....|....|....|....|....|....|....|....|....|....|....|....|....|....|....|....|

L allel Amino Acid **M..A..Q..G..Y..Q..S..T..T..I..L..S..E..*..K..E..P..T..L..T..L..V..V..P..Q..A..A..P..R..K..Y..Q..I..V**

**L allel gDNA**  **ATGGCTCAAGGCTATCAATCAACTACAATTTTGAGTGAGTAGAAAGAACCGACGCTGACCTTAGTGGTGCCCCAAGCAGCACCAAGGAAGTACCAAATAG**

**L allel cDNA**  **ATGGCTCAAGGCTATCAATCAACTACAATTTTGAGTGAGTAGAAAGAACCGACGCTGACCTTAGTGGTGCCCCAAGCAGCACCAAGGAAGTACCAAATAG**

**H allel fam35-07 gDNA** **ATGGCGCAAAGCTATCAATCAACTACAGTTTTGAGTGAGGAGAAAGAACCAACGCTGACCCTAGTGGTGCCCCAAGCAGCACCAAGGAAGTACCAAATAG**

**hv ms 07 11**  **---GCGCAAAGCTATCAATCAACTACAGTTTTGAGTGAGGAGAAAGAACCAACGCTGACCCTAGTGGTGCCCCAAGCAGCACCGAGGAAGTACCAAATAG**

**hv ms 07 13**  **---GCGCAAAGCTATCAATCAACTACAGTTTTGAGTGAGGAGAAAGAACCAACGCTGACCCTAGTGGTGCCmCAAGCAGCACCGAGGAAGTACCAAATAG**

**hv ms 07 18**  **---GCGCAAAGCTATCAATCAACTACAGTTTTGAGTGAGGAGAAAGAACCAACGCTGACCCTAGTGGTGCCmCAAGCAGCACCGAGGAAGTACCAAATAG**

**hv ms 07 3**  **---GCGCAAAGCTATCAATCAACTACAGTTTTGAGwGAGGAGAAAGAACCAACGCTGACyCTAGTGGTGCCCCAAGCAGCACCGAGGAAGTACCAAATAG**

**hv ms 08 G4**  **---GCGCAAAGCTATCAATCAACTACAGTTTTGAGTGAGGAGAAAGAACCAACGCTGACCCTAGTGGTGCCCCAAGCAGCACCmAGGAAGTACCAAATAG**

**hv ms 08 G 18**  **---GCGCAAAGCTATCAATCAACTACAGTTTTGAGTGAGGAGAAAGAACCAACGCTGACCCTAGTGGTGCCCCAAGCAGCACCGAGGAAGTACCAAATAG**

**hv ms 08 v1**  **---GCGCAAAGCTATCAATCAACTACAGTTTTGAGTGAGGAGAAAGAACCAACGCTGACCCTAGTGGTGCCCCAAGCAGCACCkAGGAAGTACCAAATAG**

**hv ms 08 vl4**  **---GCGCAAAGCTATCAATCAACTACAGTTTTrAGTGAGrAGAAAGAACCAACGCTGACCCTAGTGGTGCCCCAAGCAkCACCrAGGAAGTACCAAATAG**

**hv ms 08 vl 18**  **---GCGCAAAGCTATCAATCAACTACAGTTTTGAGTGAGGAGAAAGAACCAACGCTGACCCTAGTGGTGCCCCAAGCAGCACCmAGGAAGTACCAAATAG**

**hv ms 14**  **---GCGCAAAGCTATCAATCAACTACAGTTTTGAGTGAGGAGAAAGAACCAACGCTGACCCTAGTGGTGCCCCArGCAkCACCGAGGAAGTACCAAATAG**

**hv ms 5**  **---GCGCAAAGCTATCAATCAACTACAGTTTTGAGTGAGsAGAAAGAacCAACGCTGACCCTAGTGGTGCCCCArGCAGCACCGAGGAAGTACCAAATAG**

**hv ms vl 12 08**  **---GCGCAAAGCTATCAATCAACTACAGTTTTGAGTGAGGAGAAAGAACCAACGCTGACCCTAGTGGTGCCCCAAGCAkCACCrAGGAAGTACCAAATAG**

**hv mxe 06 10**  **---GCGCAAAGCTATCAATCAACTACAGTTTTGAGTGAGGAGAAAGAACCAACGCTGACCCTAGTGGTGCCGCAAGCATCACCGAGAAAGTACCAAATAG**

**hv mxe 06 11**  **---GCGCAAAGCTATCAATCAACTACAGTTTTGAGwGAGGAGAAAGAACCAACGCTGACCCTAGTGGTGCCCCAAGCAGCACCCAGGAAGTACCAAATAG**

**hv mxe D 121F 06**  **---GCGCAAAGCTATCAATCAACTACAGTTTTGAGTGAGGAGAArGAACCAaCGCTGACCCTAGTGGTGCCCCAAGCAGCACCrAGGAAGTACCAAATAG**

**hv mxe D 138F 06**  **---GCGCAAAGCTATCAATCAACTACAGTTTTGAGTGAGGAGAAAGAACCrACGCTGACCCTAGTrGTGCCCCAAGCAkCACCGAGGAAGTACCAAATAG**

**hv mxe E6**  **---GCGCAAAGCTATCAATCAACTACAGTTTTGAGTGArGAGAAAGAACCrACGCTGACCCTAGTGGTGCCCCAAGCAGCACCrAGGAAGTACCAAATAG**

**hv mxe E7 06**  **---GCGCAAAGCTATCAATCAACTACAGTTTTGAGTGAGGAGAAGGAACCAACGCTGACCCTAGTGGTGCCCCAAGCAGCACCAAGGAAGTACCAAATAG**

**hv nc E140F 06**  **---GCGCAAAGCTATCAATCAACTACAGTTTTGAGTGAGGAGAAAGAACCAACGCTGACyCTAGTGGTGCCCCAAGCAkCACCCAGGAAGTACCAAATAG**

**hv nc E142F 06**  **-----------------------------------------------ACCAACGCTGACCCTAGTGGTGCCCCAAGCAGCACCGAGGAAGTACCAAATAG**

**hv nc E 135F**  **---GCGCAAAGCTATCAATCAACTACAGTTTTGAGTGAGGAGAAAGAACCAACGCTGACyCTAGTGGTGCCCCAAGCAkCACCCAGGAAGTACCAAATAG**

**hv nc F4**  **---GCGCAAAGCTATCAATCAACTACAGTTTTGAGTGAGGAGAAAGAACCAACGCTGACCCTAGTGGTGCCCCArGCAGCACCGAGGAAGTACCAAATAG**

**hv nc m07-28**  **---GCGCAAAGCTATCAATCAACTACAGTTTTGAGTGAGGAGAAAGAACCAACGyTGACCCTAGTGGTGCCCCAAGCATCACCrAGGAAGTACCAAATAG**

**hv tx 06-11a**  **---GCGCAAAGCTATCAATCAACTACAGTTTTGAGTGAGGAGAAAGAACCAACGCTGACCCTAGTGGTGCCCCAAGCAkCACCAAGGAAGTACCAAATAG**

**hv tx 06 1A**  **---GCGCAAAGCTATCAATCAACTACAGTTTTGAGTGAGGAGAAAGAACCAACGCTGACCCTAGTGGTGCCCCAAGCAGCACCsAGGAAGTACCAAATAG**

**hv tx 2 07 16**  **---GCGCAAAGCTATCAATCAACTACAGTTTTGAGTGAGGAGAAAGAACCAACGCTGACyCTAGTGGTGCCCCAAGCAGCACCGAGGAAGTACCAAATAG**

**hv tx 2 07 22**  **---GCGCAAAGCTATCAATCAACTACAGTTTTrAGTGAGGAGAAAGAACCAACGCTGACCCTAGTGGTGCCCCArGCAGCACCGAGGAAGTACCAAATAG**

**hv tx 2 07 25**  **---GCGCAAAGCTATCAATCAACTACAGTTTTrAGTGAGGAGAAAGAACCAACGCTGACCCTAGTGGTGCCCCAAGCAGCACCGAGGAArTACCAAATAG**

**hv tx 2 07 27**  **---GCGCAAAGCTATCAATCAACTACAGTTTTGAGTGAGGAGAArGAACCAACGCTGACCCTAGTGGTGCCCCArGCAGCACCsAGGAAGTACCAAATAG**

**hv tx 2 07 28**  **---GCGCAAAGCTATCAATCAACTACAGTTTTGAGTGAGGAGAAAGAACCAACGCTGACCCTAGTGGTGCCCCAAGCAGCACCGAGGAAGTACCAAATAG**

**hv tx 2 07 6**  **---GCGCAAAGCTATCAATCAACTACAGTTTTrAGTGAGGAGAAAGAACCAACGCTGACCCTAGTGGTGCCCCAAGCAGCACCGAGGAArTACCAAATAG**

**hv tx 2 07 9**  **---GCGCAAAGCTATCAATCAACTACAGTTTTGAGwGAGGAGAAAGAACCAACGCTGACCCTAGTGGTGCCCCAAGCAkCACCmAGGAAGTACCAAATAG**

110 120 130 140 150 160 170 180 190 200

....|....|....|....|....|....|....|....|....|....|....|....|....|....|....|....|....|....|....|....|

L allel Amino Acid **..Y..P..N..L..I..T..F..G..Y..W..H..I..A..G..L..Y..G..L..Y..L..C..F..T..S..A..K..W..A..T..I..L..F..S**

**L allel gDNA**  **TGTATCCGAACCTTATAACGTTTGGATACTGGCACATAGCTGGTCTTTATGGCCTCTACTTGTGCTTCACTTCTGCTAAATGGGCTACAATTTTATTTA<**

**L allel cDNA**  **TGTATCCGAACCTTATAACGTTTGGATACTGGCACATAGCTGGTCTTTATGGCCTCTACTTGTGCTTCACTTCTGCTAAATGGGCTACAATTTTATTTA<**

**H allel fam35-07 gDNA** **TGTACCCGAACCTTATAACGTTTGGTTACTGGCACATAGCTGGTCTTTATGGCCTTTACTTGTGCTTCATTTCTGCTAAATGGGCTACAATTTTATTTA<**

**hv ms 07 11**  **TGTACCCAAACCTTATAACGTTTGGTTACTGGCACATAGCTGGTCTTTATGGCCTTTACTTGTGCTTCACTTCTGCTAAATGGGCTACAAT---------**

**hv ms 07 13**  **TGTAyCCGAACCTTATAACGTTTGGwTACTGGCACATAGCyGGTCTwTATGGCCTyTACTTGTGCTTCACTTCTGCTAAATGGGCTACAAT---------**

**hv ms 07 18**  **TGTACCCrAACCTTrTAACGTTTGGwTACTGGCACATAGCyGGTCTTTATGGCCTTTACTTGTGCTTCACTTCTGCTAAATGGGCTACAAT---------**

**hv ms 07 3**  **TGTACCCrAACCTTATAACGTTTGGwTACTGGCACATAGCTGGTCTTTATGGCCTTTACTTGTGCTTCACTTCTGCTAAATGGGCTACAAT---------**

**hv ms 08 G4**  **TrTACCCGAACCTyATAACGTTTGGwTACTGGCACATAGCTGGTCTTTATGGCCTTTACTTGTGCTTCACTTCTGCTAAATGGGCTACAAT---------**

**hv ms 08 G 18**  **TGTAyCCGAACCTTATAACGTTTGGwTACTGGCACATAGCyGGTCTTTAyGGCCTyTACTTGTGCTTCACTTCTGCTAAATGGGCTACAAT---------**

**hv ms 08 v1**  **TGTACCCGAACCTTATAACGTTTGGwTACTGGCACATAGCyGGTCTTTATGGCCTTTACTTGTGCTTCACTTCTGCTAAATGGGCTACAAT---------**

**hv ms 08 vl4**  **TGTACCCGAACCTTATAACGTTTGGTTACTGGCACATAGCTGGTCTTTATGGCCTyTACTTGTGCTTCACTTCTGCTAAATGGGCTACAAT---------**

**hv ms 08 vl 18**  **TGTACCCGAACCTTATAACGTTTGGwTACTGGCACATAGCTGGTCTTTATGGCCTTTACTTGTGCTTCACTTCTGCTAAATGGGCTACAAT---------**

**hv ms 14**  **TGTAyCCGAACCTTATAACGTTTGGwTACTGGCACATAGCyGGTCTTTATGGCCTTTACTTGTGCTTCACTTCTGCTAAATGGGCTACAAT---------**

**hv ms 5**  **TGTACCCrAACCTTATAACGTTTGGATACTGGCACATAGCTGGTCTTTATGGCCTTTACTTGTGCTTCACTTCTGCTAAATGGGCTACAAT---------**

**hv ms vl 12 08**  **TGTAyCCGAACCTyATAACGTTTGGTTACTGGCACATAGCyGGTCTTTATGGCCTTTACTTGTGCTTCACTTCTGCTAAATGGGCTACAAT---------**

**hv mxe 06 10**  **TGTATCCGAACCTTATAACGTTTGGTTACTGGCACATAGCCGGTCTTTATGGCCTTTACTTGTGCTTCACTTCTGCTAAATGGGCTACAAT---------**

**hv mxe 06 11**  **TGTACCCGAACCTTATAACGTTTGGTTACTGGCACATAGCyGGTCTTTATGGCCTTTACTTGTGCTTCACTTCTGCTAAATGGGCTACAAT---------**

**hv mxe D 121F 06**  **TGTAyCCGAACCTTATAACGTTTGGTTACTGGCACATAGCTGGTCTTTATGGCCTTTACTTGTGCTTCACTTCTGCTAAATGGGCTACAAT---------**

**hv mxe D 138F 06**  **TGTACCCrAACCTTATAACGTTTGGTTACTGGCACATAGCCGGTCTTTATGGCCTTTACTTGTGCTTCACTTCTGCTAAATGGGCTACAAT---------**

**hv mxe E6**  **TGTACCCrAACCTTATAACGTTTGGwTACTGGCACATAGCyGGTCTTTATGGCCTTTACTTGTGCTTCACTTCTGCTAAATGGGCTACAAT---------**

**hv mxe E7 06**  **TGTACCCGAACCTTATAACGTTTGGTTACTGGCACATAGCTGGTCTTTATGGCCTTTACTTGTGCTTCACTTCTGCTAAATGGGCTACAAT---------**

**hv nc E140F 06**  **TGTAyCCGAACCTTATwACGTTTGGTTACTGGCACATAGCyGGTCTTTATGGCCTTTACTTGTGCTTCACTTCTGCTAAATGGGCTACAAT---------**

**hv nc E142F 06**  **TGTACCCGAACCTTATAACGTTTGGATACTGGCACATAGCTGGTCTTTATGGCCTTTACTTGTGCTTCACTTCTGCTAAATGGGCTACAAT---------**

**hv nc E 135F**  **TGTAyCCGAACCTTATwACGTTTGGTTACTGGCACATAGCyGGTCTTTATGGCCTTTACTTGTGCTTCACTTCTGCTAAATGGGCTACAAT---------**

**hv nc F4**  **TGTACCCrAACCTTATAACGTTTGGwTACTGGCACATAGCTGGTCTTTATGGCCTTTACTTGTGCTTyACTTCTGCTAAATGGGCTACAAT---------**

**hv nc m07-28**  **TGTAyCCGAACCTTATAACGTTTGGTTACTGGCACATAGCyGGTCTTTATGGCCTTTACTTGTGCTTyACTTCTGCTAAATGGGCTACAAT---------**

**hv tx 06-11a**  **TGTAyCCGAACCTTATAACGTTTGGTTACTGGCACATAGCTGGTCTTTATGGCCTTTACTTGTGCTTCACTTCTGCTAAATGGGCTACAAT---------**

**hv tx 06 1A**  **TGTAyCCGAACCTTATAACGTTTGGwTACTGGCACATAGCyGGTCTTTAyGGCCTTTACTTGTGCTTyACTTCTGCTAAATGGGCTACAAT---------**

**hv tx 2 07 16**  **TGTACCCGAACCTTATAACGTTTGGTTACTGGCACATAGCyGGTCTTTATGGCCTTTACTTGTGCTTCACTTCTGCTAAATGGGCTACAAT---------**

**hv tx 2 07 22**  **TGTACCCGAACCTTATAACGTTTGGTTACTGGCACATAGCTGGTCTTTATGGCCTTTACTTGTGCTTCACTTCTGCTAAATGGGCTACAAT---------**

**hv tx 2 07 25**  **TGTACCCGAACCTTATAACGTTTGGTTAyTGGCACATAGCyGGTCTTTATGGCCTTTACTTGTGCTTCACTTCTGCTAAATGGGCTACAAT---------**

**hv tx 2 07 27**  **TGTACCCGAACCTTATAACGTTTGGTTACTGGCACATAGCyGGTCTTTATGGCCTTTACTTGTGCTTCACTTCTGCTAAATGGGCTACAAT---------**

**hv tx 2 07 28**  **TGTAyCCGAACCTTATAACGTTTGGATACTGGCACATAGCTGGTCTTTATGGCCTTTACTTGTGCTTCACTTCTGCTAAATGGGCTACAAT---------**

**hv tx 2 07 6**  **TGTACCCGAACCTTATAACGTTTGGTTAyTGGCACATAGCyGGTCTTTATGGCCTTTACTTGTGCTTCACTTCTGCTAAATGGGCTACAAT---------**

**hv tx 2 07 9**  **TGTACCCGAACCTTATAACGTTTGGTTAyTGGCACATAGCyGGTCTTTATGGCCTTTACTTGTsCTTCACTTCTGCTAAATGGGCTACAAT---------**

670 680 690 700

...|....|....|....|....|....|....|

L allel Amino Acid **..Y..F..L..F..V..V..A..E..I..G..I**

**L allel gDNA**  **>GTTACTTCCTCTTCGTGGTCGCAGAAATAGGGA**

**L allel cDNA**  **>GTTACTTCCTCTTCGTGGTCGCAGAAATAGGGA**

**H allel fam35-07 gDNA** **>GTTACTTCCTCTTCGTGGTTGCAGAAATAGGGA**

**hv ms 07 11**  **---------------------GCAGAAATAGGGA**

**hv ms 07 13**  **---------------------GCAGAAATAGGGA**

**hv ms 07 18**  **---------------------GCAGAAATAGGGA**

**hv ms 07 3**  **---------------------GCAGAAATAGGGA**

**hv ms 08 G4**  **---------------------GCAGAAATAGGGA**

**hv ms 08 G 18**  **----------------------------------**

**hv ms 08 v1**  **---------------------GCAGAAATAGGGA**

**hv ms 08 vl4**  **---------------------GCAGAAATAGGGA**

**hv ms 08 vl 18**  **---------------------GCAGAAATAgGGA**

**hv ms 14**  **---------------------GCAGAAATAGGGA**

**hv ms 5**  **---------------------GCAGAAATAGGGA**

**hv ms vl 12 08**  **---------------------GCAGAAATAGGGA**

**hv mxe 06 10**  **---------------------GCAGAAATAGGGA**

**hv mxe 06 11**  **---------------------GCAGAAATAGGGA**

**hv mxe D 121F 06**  **---------------------GCAGAAATAGGGA**

**hv mxe D 138F 06**  **---------------------GCAGAAATAGGGA**

**hv mxe E6**  **---------------------GCAGAAATAGGGA**

**hv mxe E7 06**  **---------------------GCAGAAATAGGGA**

**hv nc E140F 06**  **---------------------GCAGAAATAGGGA**

**hv nc E142F 06**  **---------------------GCAGAAATAGGGA**

**hv nc E 135F**  **---------------------GCAGAAATAGGGA**

**hv nc F4**  **---------------------GCAGAAATAGGGA**

**hv nc m07-28**  **---------------------GCAGAAATAGGGA**

**hv tx 06-11a**  **---------------------GCAGAAATAGGGA**

**hv tx 06 1A**  **---------------------GCAGAAATAGGGA**

**hv tx 2 07 16**  **---------------------GCAGAAATAGGGA**

**hv tx 2 07 22**  **---------------------GCAGAAATAGGGA**

**hv tx 2 07 25**  **---------------------GCAGAAATAGGGA**

**hv tx 2 07 27**  **---------------------GCAGAAATAGGGA**

**hv tx 2 07 28**  **---------------------GCAGAAATAGGGA**

**hv tx 2 07 6**  **---------------------GCAGAAATAGGGA**

**hv tx 2 07 9**  **---------------------GCAGAAATAGGGA**

710 720 730 740 750 760 770 780 790 800

....|....|....|....|....|....|....|....|....|....|....|....|....|....|....|....|....|....|....|....|

L allel Amino Acid **..T..A..G..A..H..R..L..W..A..H..K..T..Y..K..A..K..L..P..L..E..I..L..L..M..V..L..N..S..I..A..F..Q..N.**

**L allel gDNA**  **TCACGGCTGGCGCTCACAGGCTATGGGCGCACAAAACTTACAAAGCGAAACTACCATTAGAAATTCTCTTAATGGTACTGAACTCCATCGCTTTTCAAAA**

**L allel cDNA**  **TCACGGCTGGCGCTCACAGGCTATGGGCGCACAAAACTTACAAAGCGAAACTACCATTAGAAATTCTCTTAATGGTACTGAACTCCATCGCTTTTCAAAA**

**H allel fam35-07 gDNA** **TCACGGCTGGCGCTCACAGGCTATGGGCGCACAAAACTTATAAAGCGAAACTACCACTAGAAATTCTCTTAATGGTACTGAACTCTATCGCTTTTCAAAA**

**hv ms 07 11**  **TCACGGCTGGCGCwCACAGGCTATGGGCGCACAAAACTTATAAAGCGAAACTACCACTAGAAATTCTCTTAATGGTACTGAACTCTATCGCTTTTCAAAA**

**hv ms 07 13**  **TCACGGCTGGCGCwCACAGGyTATGGGCGCACAAAACTTATAAaGCGAAACTACCACTAGAAATTCTCTTAATGGTACTGAACTCTATCGCTTTTCAAAA**

**hv ms 07 18**  **TCACGGCTGGCGCACACAGGCTATGGGCGCACAAAACTTAyAAaGCGAAACTACCAyTAGAAATyCTCTTAATGGTAyTGAACTCTATCGCTTTTCAAAA**

**hv ms 07 3**  **TCACGGCTGGCGCACACAGGCTATGGGCGCACAAAACTTACaAAGCGAAACTACCATTAGAAATTCTCTTAATGrTACTkAACTCTATCGCTTTTCAAAA**

**hv ms 08 G4**  **TCACGGCTGGCGCTCACAGGCTATGGGCGCACAAAACTTATAAAGCGAAACTACCAyTAGAAATTCTCTTAATGGTACTGAACTCTATCGCwTTTCAAAA**

**hv ms 08 G 18**  **------------------------------------CTTATAAAGCGAAACTACCAyTAGAAATTCTCTTAATGGTACTGAACTCTATCGCTTTTCAAAA**

**hv ms 08 v1**  **TCACGGCTGGCGCACACAGGCTATGGGCGCACAAaACTTAyAAAGCGAAACTACCATTAGAAATTCTCTTAATGrTAyTkAACTCTATCGCTTTTCAAAA**

**hv ms 08 vl4**  **TCACGGCTGGCGCACACAGGCTATGGGCGCACAAaACTTAyaAAGCGAAACTACCAyTAGAAATTCTCTTAATGrTACTkAACTCTATCGCTTTTCAAAA**

**hv ms 08 vl 18**  **TCACGGCTGGCGCTCACAGGCTATGGGCGCACAAAACTTACAAAGCGAAACTACCATTAGAAATTCTCTTAATGGTACTGAACTCTATCGCTTTTCAAAA**

**hv ms 14**  **TCACGGCTGGCGCTCACAGGCTATGGGCsCACAAAACTTACAAaGCsAAaCTACCATTAGAAATTCTCTTAATGrTACTkAACTCTATCGCTTTTCAAAA**

**hv ms 5**  **TCACGGCTGGCGCwCACAGGCTATGGGCGCACAAAACTTAyAAAGCGAAACTACCAyTAGAAATTCTCTTAATGGTACTGAACTCTATCGCTTTTCAAAA**

**hv ms vl 12 08**  **TCACGGCTGGCGCTCACAGGyTATGGGCGCACAAAACTTATAAAGCGAAACTACCAyTAGAAATTCTCTTAATGrTAyTkAACTCTATCGCTTTTCAAAA**

**hv mxe 06 10**  **TCACGGCTGGCGCwCACAGGCTATGGGCsCACAAAACTTATAAAGCGAAACTACCAyTAGAAATTCTCTTAATGGTACTGAACTCTATCGCTTTTCAAAA**

**hv mxe 06 11**  **TCACGGCTGGCGCwCACAGGCTATGGGCGCACAAAACTTACAAaGCGAAACTACCATTAGAAATTCTCTTAATGrTACTkAACTCTATCGCTTTTCAAAA**

**hv mxe D 121F 06**  **TCACGGCTGGCGCwCACAGGCTATGGGCGCACAAAACTTAyAAaGCGAAACTACCAyTAGAAATTCTCTTAATGGTACTGAACTCTATCGCTTTTCAAAA**

**hv mxe D 138F 06**  **TCACGGCTGGCGCwCACAGGCTATGGGCGCACAAAACTTAyAAaGCGAAACTACCAyTAGAAATTCTCTTAATGGTACTGAACTCTATCGCwTTTCAAAA**

**hv mxe E6**  **TCACGGCTGGCGCACACAGGCTATGGGCGCACAAAACTTACaAAGCGAAACTACCATTAGAAATTCTCTTAATGrTACTkAACTCTATCGCTTTTCAAAA**

**hv mxe E7 06**  **TCACGGCTGGCGCACACAGGCTATGGGCGCACAAAACTTACAAaGCGAAACTACCATTAGAAATTCTCTTAATGGTACTGAACTCTATCGCTTTTCAAAA**

**hv nc E140F 06**  **TCACGGCTGGyGCwCACAGGCTrTGGGCGCACAAAACTTATAAAGCGAAACTACCAyTAGAAATTCTCTTAATGGTACTGAACTCTATCGCTTTTCAAAA**

**hv nc E142F 06**  **TCACGGCTGGCGCACACAGGCTATGGGCGCACAAAACTTAyAAaGCGAAACTACCAyTAGAAATyCTCTTAATGGTAyTGAACTCTATCGCTTTTCAAAA**

**hv nc E 135F**  **TCACGGCTGGyGCwCACAGGCTrTGGGCGCACAAAACTTATAAAGCGAAACTACCAyTAGAAATTCTCTTAATGGTACTGAACTCTATCGCTTTTCAAAA**

**hv nc F4**  **TCACGGCTGGCGCwCACAGGCTATGGGCGCACAAAACTTATAAAGCGAAACTACCACTAGAAATTCTCTTAATGGTACTGAACTCTATCGCTTTTCAAAA**

**hv nc m07-28**  **TCACGGCTGGCGCTCACAGGCTATGGGCGCACAAAACTTAyAAaGCGAAACTACCAyTAGAAATTCTCTTAATGGTACTGAACTCyATCGCwTTTCAAAA**

**hv tx 06-11a**  **TCACGGCTGGCGCTCACAGGCTATGGGCGCACAAAACTTAyAAaGCGAAACTACCAyTAGAAATTCTCTTAATGGTACTGAACTCTATCGCTTTTCAAAA**

**hv tx 06 1A**  **TCACGGCTGGCGCTCACAGGCTATGGGCGCACAAAACTTATAAAGCGAAACTACCAyTAGAAATTCTCTTAATrrTACTGAACTCTATCGCTTTTCAAAA**

**hv tx 2 07 16**  **TCACGGCTGGCGCwCACAGGCTATGGGCGCACAAAACTTAyAAaGCGAAACTACCAyTAGAAATTCTCTTAATGGTACTGAACTCTATCGCTTTTCAAAA**

**hv tx 2 07 22**  **TCACGGCTGGCGCACACAGGCTATGGGCGCACAAAACTTAyAAaGCGAAACTACCAyTAGAAATyCTCTTAATGGTAyTGAACTCTATCGCTTTTCAAAA**

**hv tx 2 07 25**  **TCACGGCTGGCGCwCACAGGCTATGGGCGCACAAAACTTAyAAaGCGAAACTACCAyTAGAAATTCTCTTAATGGTACTGAACTCTATCGCwTTTCAAAA**

**hv tx 2 07 27**  **TCACGGCTGGyGCwCACAGGCTrTGGGCGCACAAAACTTATAAAGCGAAACTACCAyTAGAAATTCTCTTAATGGTACTGAACTCTATCGCTTTTCAAAA**

**hv tx 2 07 28**  **TCACGGCTGGCGCACACAGGCTATGGGCGCACAAAACTTACAAaGCGAAACTACCATTAGAAATTCTCTTAATGGTACTGAACTCTATCGCTTTTCAAAA**

**hv tx 2 07 6**  **TCACGGCTGGyGCTCACAGGyTATGGGCGCACAAAACTTAyAAaGCGAAACTACCATTAGAAATTCTCTTAATGrTACTkAACTCTATCGCTTTTCAAAA**

**hv tx 2 07 9**  **TCACGGCTGGyGCwCACAGGCTrTGGGCGCACAAAACTTATAAAGCGAAACTACCAyTAGAAATTCTCTTAATGGTACTGAACTCTATCGCTTTTCAAAA**

810 820 830 840 850 860 870 880 890 900

....|....|....|....|....|....|....|....|....|....|....|....|....|....|....|....|....|....|....|....|

L allel Amino Acid **.S..A..I..D..W..V..R..D..H..R..L..H..H..K..Y..S..D..T..D..A..D..P..H..N..A..S..R..G..F..F..Y..S..H..**

**L allel gDNA**  **CTCAGCCATTGACTGGGTGAGGGACCATCGTCTCCATCATAAGTACAGCGATACTGATGCTGATCCCCATAATGCTAGCCGAGGGTTCTTTTATTCCCAT**

**L allel cDNA**  **CTCAGCCATTGACTGGGTGAGGGACCATCGTCTCCATCATAAGTACAGCGATACTGATGCTGATCCCCATAATGCTAGCCGAGGGTTCTTTTATTCCCAT**

**H allel fam35-07 gDNA** **CTCCGCCATTGACTGGGTGAGGGACCATCGTCTCCATCATAAGTACAGCGATACTGATGCTGATCCCCATAATGCTAGCCGAGGGTTCTTTTATTCCCAT**

**hv ms 07 11**  **CTCmGCCATTGACTGGGTGAGGGACCATCGTCTyCATCATAAGTACAGCGATACwGATGCTGATCCTCATAATGCTAGCCGAGGGTTCTTTTATTCCCAy**

**hv ms 07 13**  **CTCsGCCATTGACTGGGTGAGGGACCATCGTCTyCATCATAAGTACAGCGATACAGATGCTGATCCTCATAATGCTAGCCGAGGGTTCTTTTATTCCCAy**

**hv ms 07 18**  **CTCrGCCATTGACTGGGTGAGGGACCATCGTCTwCATCATAAGTACAGCGAyACAGATGCTGATCCTCATAATGCTAGCCGAGGGTTCTTTTATTCCCAy**

**hv ms 07 3**  **CTCmGCCATTGACTGGGTGAGGGACCATCGTCTCCATCATAAGTACAGCGATACAGATGCTGATCCyCATAATGCTAGCCGAGGGTTCTTTTATTCCCAT**

**hv ms 08 G4**  **CTCrGCCATTGACTGGGTGAGGGACCATCGTCTCCATCATAAGTACAGCGATACwGATGCTGATCCTCATAATGCTAGCCGAGGGTTCTTTTATTCCCAy**

**hv ms 08 G 18**  **CTCCGCCATTGACTGGGTGAGGGACCATCGTCTCCATCATAAGTACAGCGAyACAGATGCTGATCCTCATAATGCTAGCCGAGGGTTCTTTTATTCCCAT**

**hv ms 08 v1**  **CTCrGCCATTGACTGGGTGAGGGACCATCGTCTCCATCATAAGTAyAGCGATACAGATGCTGATCCTCATAATGCTAGCCGAGGGTTCTTTTATTCCCAy**

**hv ms 08 vl4**  **CTCrGCCATTGACTGGGTGAGGGACCATCGTCTyCATCATAAGTACAGCGATACAGATGCTGATCCTCATAATGCTAGCCGAGGGTTCTTTTATTCCCAy**

**hv ms 08 vl 18**  **CTCAGCCATTGACTGGGTGAGGGACCATCGTCTCCATCATAAGTACAGCGATACwGATGCTGATCCTCATAATGCTAGCCGAGGGTTyTTTTATTCCCAy**

**hv ms 14**  **CTCrGCCATTGACTGGGTGAGGGACCATCGTCTCCATCATAAGTACAGCGAyACAGATGCTGATCCTCATAATGCTAGCCGAGGGTTCTTTTATTCCCAT**

**hv ms 5**  **CTCrGCCATTGACTGGGTGAGGGACCATCGTCTyCATCATAAGTACAGCGAyACAGATGCTGATCCTCATAATGCTAGCCGAGGGTTCTTTTATTCCCAy**

**hv ms vl 12 08**  **CTCmGCCATTGACTGGGTGAGGGACCATCGTCTCCATCATAAGTACAGCGACACAGATGCTGATCCTCATAATGCTAGCCGAGGGTTCTTTTATTCCCAT**

**hv mxe 06 10**  **CTCsGCCATTGAyTGGGTGAGGGACCATCGTCTCCATCATAAGTACAGCGATACAGATGCTGATCCTCATAATGCTAGCCGAGGGTTCTTTTATTCCCAT**

**hv mxe 06 11**  **CTCAGCCATTGACTGGGTGAGGGACCATCGTCTCCATCATAAGTACAGCGACACAGATGCTGATCCTCATAATGCTAGCCGAGGGTTCTTTTATTCCCAT**

**hv mxe D 121F 06**  **CTCGGCCATTGACTGGGTGAGGGACCATCGTCTCCATCATAAGTACAGCGAyACAGATGCTGATCCTCATAATGCTAGCCGAGGGTTCTTTTATTCCCAT**

**hv mxe D 138F 06**  **CTCsGCCATTGACTGGGTGAGGGACCATCGTCTCCATCATAAGTACAGCGATACAGATGCTGAyCCmCATAATGCTAGCCGAGGGTTCTTTTATTCCCAT**

**hv mxe E6**  **CTCmGCCATTGACTGGGTGAGGGACCATCGTCTCCATCATAAGTACAGCGATACAGATGCTGATCCyCATAATGCTAGCCGAGGGTTCTTTTATTCCCAT**

**hv mxe E7 06**  **CTCGGCCATTGACTGGGTGAGGGACCATCGTCTCCATCATAAGTACAGCGACACAGATGCTGATCCTCATAATGCTAGCCGAGGGTTCTTTTATTCCCAT**

**hv nc E140F 06**  **CTCCGCCATTGACTGGGTGAGGGACCATCGTCTCCATCATAAGTACAGCGAyACAGATGCTGATCCTCATAATGCTAGCmGAGGGTTTTTTTATTCCCAT**

**hv nc E142F 06**  **CTCrGCCATTGACTGGGTGAGGGACCATCGTCTTCATCATAAGTACAGCGAyACAGATGCTGATCCTCATAATGCTAGCCGAGGGTTCTTTTATTCCCAy**

**hv nc E 135F**  **CTCCGCCATTGACTGGGTGAGGGACCATCGTCTCCATCATAAGTACAGCGAyACAGATGCTGATCCTCATAATGCTAGCmGAGGGTTTTTTTATTCCCAT**

**hv nc F4**  **CTCrGCCATTGACTGGGTGAGGGACCATCGTCTyCATCATAAGTACAGCGATACwGATGCTGATCCTCATAATGCTAGCCGAGGGTTCTTTTATTCCCAy**

**hv nc m07-28**  **CTCrGCCATTGACTGGGTGAGGGACCATCGTCTCCATCATAAGTACAGCGATACAGATGCTGAyCCwCATAATGCTAGCCGAGGGTTCTTTTATTCCCAy**

**hv tx 06-11a**  **CTCmGCCATTGACTGGGTGAGGGACCATCGTCTCCATCATAAGTACAGCGATACwGATGCTGATCCTCATAATGCTAGCCGAGGGTTyTTTTATTCCCAT**

**hv tx 06 1A**  **CTCrGCCATTGACTGGGTGAGGGACCATCGTCTCCATCATAAGTACAGCGATACTGATGCTGAyCCwCATAATGCTAGCCGAGGGTTyTTTTATTCCCAy**

**hv tx 2 07 16**  **CTCGGCCATTGACTGGGTGAGGGACCATCGTCTCCATCATAAGTACAGCGAyACAGATGCTGATCCTCATAATGCTAGCCGAGGGTTCTTTTATTCCCAT**

**hv tx 2 07 22**  **CTCrGCCATTGACTGGGTGAGGGACCATCGTCTwCATCATAAGTACAGCGAyACAGATGCTGATCCTCATAATGCTAGCCGAGGGTTCTTTTATTCCCAy**

**hv tx 2 07 25**  **CTCsGCCATTGACTGGGTGAGGGACCATCGTCTCCATCATAAGTACAGCGATACAGATGCTGAyCCmCATAATGCTAGCCGAGGGTTCTTTTATTCCCAT**

**hv tx 2 07 27**  **CTCCGCCATTGACTGGGTGAGGGACCATCGTCTCCATCATAAGTACAGCGAyACAGATGCTGATCCTCATAATGCTAGCmGAGGGTTTTTTTATTCCCAT**

**hv tx 2 07 28**  **CTCGGCCATTGACTGGGTGAGGGACCATCGTCTCCATCATAAGTACAGCGACACAGATGCTGATCCTCATAATGCTAGCCGAGGGTTCTTTTATTCCCAT**

**hv tx 2 07 6**  **CTCAGCCATTGACTGGGTGAGGGACCATCGTCTCCATCATAAGTACAGCGACACAGATGCTGATCCTCATAATGCTAGCCGAGGGTTCTTTTATTCCCAT**

**hv tx 2 07 9**  **CTCCGCCATTGACTGGGTGAGGGACCATCGTCTCCATCATAAGTACAGCGAyACAGATGCTGATCCTCATAATGCTAGCmGAGGGTTTTTTTATTCCCAT**

910 920 930 940 950 960 970 980 990 1000

....|....|....|....|....|....|....|....|....|....|....|....|....|....|....|....|....|....|....|....|....

L allel Amino Acid **V..G..W..L..L..V..R..K..H..P..E..V..K..K..R..G..R..E..L..N..M..S..D..I..Y..N..N..P..V..L..R..F..Q..K..K.**

**L allel gDNA**  **GTAGGATGGCTACTCGTTAGAAAACATCCTGAAGTCAAAAAACGTGGCAGAGAACTCAATATGTCTGATATCTATAACAATCCAGTGCTGCGGTTTCAGAAAAA<**

**L allel cDNA**  **GTAGGATGGCTACTCGTTAGAAAACATCCTGAAGTCAAAAAACGTGGCAGAGAACTCAATATGTCTGATATCTATAACAATCCAGTGCTGCGGTTTCAGAAAAA<**

**H allel fam35-07 gDNA** **GTAGGATGGCTACTCGTTAGAAAACACCCTGAAGTCAAAAAACGTGGGAGAGAACTCAATATGTCTGATATTTATAACAATCCAGTGCTGCGGTTTCAGAAAAA<**

**hv ms 07 11**  **GTAGGATGGCTACTyGTTAGAAAACACCCTGAAGTCAAAAAACGTGGsArAGAACTCAATATGTCTGATATTTAyAACAATCCA----------------**

**hv ms 07 13**  **GTAGGATGGCTACTCGTTAGAAAACACCCTGAAGTCAAAAAACGTGGCAGAGAACTCAATATGTCTGATATTTATAACAATCCA----------------**

**hv ms 07 18**  **GTAGGATGGCTACTyGTTAGAAAACACCCTGAAGTCAAAAAACGTGGGAGAGAACTCAATATGTCTGATATTTAyAACAATCCA----------------**

**hv ms 07 3**  **GTwGGrTGGCTACTCGTTAGAAAACATCCTGAAGTCAAAAAACGTGGsArAGAACTCAATATGTCTGATATTTAyAACAATCCA----------------**

**hv ms 08 G4**  **GTAGGATGGCTACTCGTTAGAAAACACCCTGAAGTCAAAAAACGTGGkAGAGAACTCAATATGTCTGATATTTATAACAATCCA----------------**

**hv ms 08 G 18**  **GTAGGATGGCTACTCGTTAGAAAACAyCCTGAAGTCAAAAAACGTGGCArAGAACTCAATATGTCTGATATTTATAACAATCCA----------------**

**hv ms 08 v1**  **GTAGGATGGCTAyTCGTTAGAAAACAyCCTGAAGTCAAAAAACGTGGGAAAGAACTCAATATGTCTGATATTTATAACAATCCA----------------**

**hv ms 08 vl4**  **GTAGGrTGGCTACTCGTTAGAAAACAyCCTGAAGTCAAAAAACGTGGGArAGAACTCAATATGTCTGATATTTAyAACAATCCA----------------**

**hv ms 08 vl 18**  **GTAGGATGGCTACTyGTTAGAAAACACCCTGAAGTCAAAAAACGTGGCArAGAACTCAATATGTCTGATATTTATAACAATCCA----------------**

**hv ms 14**  **GTrGGATGGCTACTyGTTAGAAAACACCCTGAAGTCAAAAAACGTGGGArAGAACTCAATATGTCTGATATTTATAACAATCCA----------------**

**hv ms 5**  **GTAGGATGGCTACTyGTTAGAAAACACCCTGAAGTCAArAAACGTGGGAGAGAACTCAATATGTCTGATATTTAyArCAATCCA----------------**

**hv ms vl 12 08**  **GTAGGATGGCTACTyGTTAGAAAACAyCCTGAAGTCAAAAAACGTGGsArAGAACTCAATATGTCTGATATTTATAACAATCCA----------------**

**hv mxe 06 10**  **GTwGGATGGCTACTCGTTAGAAAACAyCCTGAAGTCAAAAAACGTGGsAAAGAACTCAATATGTCTGATATTTATAACAATCCA----------------**

**hv mxe 06 11**  **GTAGGATGGCTACTTGTTAGAAAACACCCTGAAGTCAAAAAACGTGGGAGAGAACTCAATATGTCTGATATTTATAACAATCCA----------------**

**hv mxe D 121F 06**  **GTAGGATGGCTACTyGTTAGAAAACACCCTGAAGTCAAAAAACGTGGkArAGAACTCAATATGTCTGATATTTATAACAATCCA----------------**

**hv mxe D 138F 06**  **GTwGGATGGCTACTCGTTAGAAAACAyCCTGAAGTCAAAAAACGTGGCArAGAACTCAATATGTCTGATATTTAyAACAATCCA----------------**

**hv mxe E6**  **GTwGGrTGGCTACTCGTTAGAAAACATCCTGAAGTCAAAAAACGTGGsArAGAACTCAATATGTCTGATATTTAyAACAATCCA----------------**

**hv mxe E7 06**  **GTAGGATGGCTACTTGTTAGAAAACACCCTGAAGTCAAAAAACGTGGGAAAGAACTCAATATGTCTGATATTTATAACAATCCA----------------**

**hv nc E140F 06**  **GTAGGrTGGCTACTCGTTAGAAAACATCCTGAAGTCAAAAAACGTGGGAAAGAACTCAATATGTCTGATATTTATAACAATCCA----------------**

**hv nc E142F 06**  **GTAGGATGGCTACTyGTTAGAAAACACCCTGAAGTCAAAAAACGTGGGAGAGAACTCAATATGTCTGATATTTAyAACAATCCA----------------**

**hv nc E 135F**  **GTAGGrTGGCTACTCGTTAGAAAACATCCTGAAGTCAAAAAACGTGGGAAAGAACTCAATATGTCTGATATTTATAACAATCCA----------------**

**hv nc F4**  **GTAGGATGGCTACTyGTTAGAAAACACCCTGAAGTCAAAAAACGTGGsArAGAACTCAATATGTCTGATATTTAyAACAATCCA----------------**

**hv nc m07-28**  **GTAGGATGGCTACTCGTTAGAAAACACCCTGAAGTCAAAAAACGTGGCArAGAACTCAATATGTCTGATATTTATAACAATCCA----------------**

**hv tx 06-11a**  **GTTGGATGGCTACTCGTwAGAAAACAyCyTGAAGTCAAAAAACGTGGsAAAGAACTCAATATGTCTGATATTTATAACAATCCA----------------**

**hv tx 06 1A**  **GTAGGATGGCTACTCGTTAGAAAACACCCTGAAGTCAAAAAACGTGGsArAGAACTCAATATGTCTGATATTTATAmCAATCCA----------------**

**hv tx 2 07 16**  **GTAGGATGGCTACTyGTTAGAAAACACCCTGAAGTCAAAAAACGTGGkArAGAACTCAATATGTCTGATATTTATAACAATCCA----------------**

**hv tx 2 07 22**  **GTAGGATGGCTACTyGTTAGAAAACACCCTGAAGTCAAAAAACGTGGGAGAGAACTCAATATGTCTGATATTTAyAACAATCCA----------------**

**hv tx 2 07 25**  **GTwGGATGGCTACTCGTTAGAAAACAyCCTGAAGTCAAAAAACGTGGCArAGAACTCAATATGTCTGATATTTAyAACAATCCA----------------**

**hv tx 2 07 27**  **GTAGGrTGGCTACTCGTTAGAAAACATCCTGAAGTCAAAAAACGTGGGAAAGAACTCAATATGTCTGATATTTATAACAATCCA----------------**

**hv tx 2 07 28**  **GTAGGATGGCTACTTGTTAGAAAACACCCTGAAGTCAAAAAACGTGGGAAAGAACTCAATATGTCTGATATTTATAACAATCCA----------------**

**hv tx 2 07 6**  **GTAGGATGGCTACTTGTTAGAAAACACCCTGAAGTCAArAAACGTGGGAGAGAACTCAATATGTCTGATATTTATArCAATCCA----------------**

**hv tx 2 07 9**  **GTAGGrTGGCTACTCGTTAGAAAACATCCTGAAGTCAAAAAACGTGGGAAAGAACTCAATATGTCTGATATTTATAACAATCCA----------------**

1390 1400

|....|....|

L allel Amino Acid **.Y..A..V..**

**L allel gDNA**  **>ATACGCCGTA**

**L allel cDNA**  **>ATACGCCGTA**

**H allel fam35-07 gDNA** **>GTACGCCATA**

**hv ms 07 11**  **-----------**

**hv ms 07 13**  **-----------**

**hv ms 07 18**  **-----------**

**hv ms 07 3**  **-----------**

**hv ms 08 G4**  **-----------**

**hv ms 08 G 18**  **-----------**

**hv ms 08 v1**  **-----------**

**hv ms 08 vl4**  **-----------**

**hv ms 08 vl 18**  **-----------**

**hv ms 14**  **-----------**

**hv ms 5**  **-----------**

**hv ms vl 12 08**  **-----------**

**hv mxe 06 10**  **-----------**

**hv mxe 06 11**  **-----------**

**hv mxe D 121F 06**  **-----------**

**hv mxe D 138F 06**  **-----------**

**hv mxe E6**  **-----------**

**hv mxe E7 06**  **-----------**

**hv nc E140F 06**  **-----------**

**hv nc E142F 06**  **-----------**

**hv nc E 135F**  **-----------**

**hv nc F4**  **-----------**

**hv nc m07-28**  **-----------**

**hv tx 06-11a**  **-----------**

**hv tx 06 1A**  **-----------**

**hv tx 2 07 16**  **-----------**

**hv tx 2 07 22**  **-----------**

**hv tx 2 07 25**  **-----------**

**hv tx 2 07 27**  **-----------**

**hv tx 2 07 28**  **-----------**

**hv tx 2 07 6**  **-----------**

**hv tx 2 07 9**  **-----------**

1410 1420 1430 1440 1450 1460 1470 1480 1490 1500

....|....|....|....|....|....|....|....|....|....|....|....|....|....|....|....|....|....|....|....|

L allel Amino Acid **P..F..I..G..A..V..C..F..V..L..P..T..L..I..P..V..Y..C..W..G..E..T..W..S..N..A..W..H..I..T..M..L..R..Y**

**L allel gDNA**  **CCCTTCATTGGAGCTGTTTGTTTCGTCTTACCTACATTAATACCCGTCTACTGCTGGGGAGAAACCTGGTCCAATGCCTGGCACATCACCATGCTTCGTT**

**L allel cDNA**  **CCCTTCATTGGAGCTGTTTGTTTCGTCTTACCTACATTAATACCCGTCTACTGCTGGGGAGAAACCTGGTCCAATGCCTGGCACATCACCATGCTTCGTT**

**H allel fam35-07 gDNA** **CCCTTCATCGGAGCTGTTTGTTTCGTCTTACCTACATTGATACCCGTCTACTGCTGGGGAGAAACCTGGTCCAATGCCTGGCACATCACCATGCTTCGTT**

**hv ms 07 11**  **--------------------------------------------------CTGCTGGGGAGaAACCTGGTCCAATGCCTGGCAyATCACCATGCTTCGTT**

**hv ms 07 13**  **--------------------------------------------------CTGCTGGGGAGAAaCCTGGTCCAATGCCTGGCATATCACCATGCTTCGTT**

**hv ms 07 18**  **--------------------------------------------------CTGCTGGGGAGaAACCTGGTCCAATGCCTGGCACATCACCATGCTTCGTT**

**hv ms 07 3**  **--------------------------------------------------CTGCTGGGGAGAAaCCTGGTCCAATGCCTGGCACATCACCATGCTTCGTT**

**hv ms 08 G4**  **--------------------------------------------------CTGCTGGGGAGAAaCCTGGTCCAATGCCTGGCACATCACCATGCTTCGTT**

**hv ms 08 G 18**  **--------------------------------------------------CTGCTGGGGAGAAaCCTGGTCCAATGCCTGGCATATCACCATGCTTCGTT**

**hv ms 08 v1**  **--------------------------------------------------CTGCTGGGGAGaAACCTGGTCCAATGCCTGGCATATCACCATGCTTCGTT**

**hv ms 08 vl4**  **--------------------------------------------------CTGCTGGGGAGAAaCCTGGTCCAATGCCTGGCACATCACCATGCTTCGTT**

**hv ms 08 vl 18**  **--------------------------------------------------CTGCTGGGGAGAAaCCTGGTCCAATGCCTGGCACATCACCATGCTTCGTT**

**hv ms 14**  **--------------------------------------------------CTGCTGGGGAGAAaCCTGGTCCAATGCCTGGCACATCACCATGCTTCGTT**

**hv ms 5**  **--------------------------------------------------CTGCTGGGGAGaAACCTGGTCCAATGCCTGGCAyATCACCATGCTTCGTT**

**hv ms vl 12 08**  **--------------------------------------------------CTGCTGGGGAGaAACCTGGTCCAATGCCTGGCAyATCACCATGCTTCGTT**

**hv mxe 06 10**  **--------------------------------------------------CTGCTGGGGAGAAaCCTGGTCCAATGCCTGGCACATCACCATGCTTCGTT**

**hv mxe 06 11**  **--------------------------------------------------CTGCTGGGGAGaAACCTGGTCCAATGCCTGGCAyATCACCATGCTTCGTT**

**hv mxe D 121F 06**  **--------------------------------------------------CTGCTGGGGAGAAaCCTGGTCCAATGCCTGGCACATCACCATGCTTCGTT**

**hv mxe D 138F 06**  **--------------------------------------------------CTGCTGGGGAGAAaCCTGGTCCAATGCCTGGCACATCACCATGCTTCGTT**

**hv mxe E6**  **--------------------------------------------------CTGCTGGGGAGAAaCCTGGTCCAATGCCTGGCACATCACCATGCTTCGTT**

**hv mxe E7 06**  **--------------------------------------------------CTGCTGGGGAGAAaCCTGGTCCAATGCCTGGCACATCACCATGCTTCGTT**

**hv nc E140F 06**  **--------------------------------------------------CTGCTGGGGAGAAaCCTGGTCCAATGCCTGGCAyATCACCATGCTTCGTT**

**hv nc E142F 06**  **--------------------------------------------------CTGCTGGGGAGAAaCCTGGTCCAATGCCTGGCAyATCACCATGCTTCGTT**

**hv nc E 135F**  **--------------------------------------------------CTGCTGGGGAGAAaCCTGGTCCAATGCCTGGCAyATCACCATGCTTCGTT**

**hv nc F4**  **--------------------------------------------------CTGCTGGGGAGaAACCTGGTCCAATGCyTGGCAyATCACCATGCTTCGTT**

**hv nc m07-28**  **--------------------------------------------------CTGCTGGGGAGAAaCCTGGTCCAATGCCTGGCACATCACCATGCTTCGTT**

**hv tx 06-11a**  **--------------------------------------------------CTGCTGGGGAGAAaCCTGGTCCAATGCCTGGCACATCACCATGCTTCGTT**

**hv tx 06 1A**  **--------------------------------------------------CTGCTGGGGAGAAaCCTGGTCCAATGCCTGGCACATCACCATGCTTCGTT**

**hv tx 2 07 16**  **--------------------------------------------------CTGCTGGGGAGAAaCCTGGTCCAATGCCTGGCACATCACCATGCTTCGTT**

**hv tx 2 07 22**  **--------------------------------------------------CTGCTGGGGAGaAACCTGGTCCAATGCCTGGCAyATCACCATGCTTCGTT**

**hv tx 2 07 25**  **--------------------------------------------------CTGCTGGGGAGAAaCCTGGTCCAATGCCTGGCACATCACCATGCTTCGTT**

**hv tx 2 07 27**  **--------------------------------------------------CTGCTGGGGAGAaACCTGGTCCAATGCCTGGCACATCACCATGCTTCGTT**

**hv tx 2 07 28**  **--------------------------------------------------CTGCTGGGGAGAAaCCTGGTCCAATGCCTGGCACATCACCATGCTTCGTT**

**hv tx 2 07 6**  **--------------------------------------------------CTGCTGGGGAGAAaCCTGGTCCAATGCCTGGCACATCACCATGCTTCGTT**

**hv tx 2 07 9**  **--------------------------------------------------CTGCTGGGGAGAAaCCTGGTCCAATGCCTGGCAyATCACCATGCTTCGTT**

1510 1520 1530 1540 1550 1560 1570 1580 1590 1600

....|....|....|....|....|....|....|....|....|....|....|....|....|....|....|....|....|....|....|....|

L allel Amino Acid **..I..M..N..L..N..V..T..F..L..V..N..S..A..A..H..I..W..G..Y..K..P..Y..D..A..K..I..L..P..V..Q..N..V..A.**

**L allel gDNA**  **ACATCATGAACCTCAACGTCACGTTTTTGGTGAACAGCGCCGCTCACATATGGGGTTACAAGCCTTATGACGCGAAAATATTACCTGTCCAAAATGTAGC**

**L allel cDNA**  **ACATCATGAACCTCAACGTCACGTTTTTGGTGAACAGCGCCGCTCACATATGGGGTTACAAGCCTTATGACGCGAAAATATTACCTGTCCAAAATGTAGC**

**H allel fam35-07 gDNA** **ACATCATGAACCTCAACGTCACGTTTTTGGTGAACAGCGCCGCTCACATATGGGGCTACAAGCCTTATGACGCGAAAATATTACCTGTCCAAAACGTAGC**

**hv ms 07 11**  **AyATCATGAACCTCAACGTCACGTTTTTGGTGAACAGCGCCGCTCACATATGGGGCTACAAGCCTTATGACGCGAAAATATTACCTGTCCAAAATGTAGC**

**hv ms 07 13**  **ACATCATGAACCTCAACGTCACGTTTTTrGTGAACAGCGCCGCTCACATATGGGGCTACAAGCCTTATGACGCGAAAATATTACCTGyCCAAAAyGTAGC**

**hv ms 07 18**  **ACATCATGAAyCTCAACGTCACGTTTTTGGTGAACAGCGCCGCTCACATATGGGGCTACAAGCCTTATGACGCGAAAATATTACCTGyCCAAAACGTAGC**

**hv ms 07 3**  **ACATCATGAACCTCAACGTCACGTTTTTGGTGAACAGCGCCGCTCACATATGGGGyTACAAGCCTTATGACGCGAAAATATTACCTGyCCAAAAyGTAGC**

**hv ms 08 G4**  **ACATCATGAACCTCAACGTCACGTTTTTGGTGAACAGCGCCGCTCACATATGGGGCTACAAGCCTTATGACGCGAAAATATTACCTGTCCArAATGTAGC**

**hv ms 08 G 18**  **ACATCATGAACCTCAACGTCACGTTTTTGGTGAACAGCGCCGCTCACATATGGGGCTACAAGCCTTATGACGCGAAAATATTACCTGyCCAAAAyGTAGC**

**hv ms 08 v1**  **ACATCATGAACCTCAACGTCACGTTTTTrGTGAACAGCGCCGCTCACATATGGGGyTACAAGCCTTATGACGCGAAAATATTACCTGyCCAAAATGTAGC**

**hv ms 08 vl4**  **ACATCATGAAyCTCAACGTCACGTTTTTGGTGAACAGCGCCGCTCACATATGGGGCTACAAGCCTTATGACGCGAAAATATTACCTGTCCAAAAyGTAGC**

**hv ms 08 vl 18**  **ACATCATGAACCTCAACGTCACGTTTTTGGTGAACAGCGCCGCTCACATATGGGGCTACAAGCCTTAyGACGCGAAAATATTACCTGyCCAAAAyGTAGC**

**hv ms 14**  **ACATCATGAACCTCAACGTCACGTTTTTGGTGAACAGCGCCGCTCACATATGGGGyTACAAGCCTTAyGACGCGAAAATATTACCTGCCCAAAAyGTAGC**

**hv ms 5**  **ACATCATGAACCTCAACGTCACGTTyTTGGTGAACAGCGCCGCTCACATATGGGGyTACAAGCCTTATGACGCGAAAATATTACCTGyCCAAAACGTAGC**

**hv ms vl 12 08**  **ACATCATGAACCTCAACGTCACGTTTTTAGTGAACAGCGCCGCTCACATATGGGGyTACAAGCCTTATGACGCGAAAATATTACCTGyCCAAAATGTAGC**

**hv mxe 06 10**  **ACATCATGAACCTCAACGTCACGTTTTTGGTGAACAGCGCCGCTCACATATGGGGCTACAAGCCTTATGACGCGAAAATATTACCTGyCCAAAAyGTAGC**

**hv mxe 06 11**  **ACATCATGAACCTCAACGTCACGTTTTTrGTGAACAGCGCCGCTCACATATGGGGCTACAAGCCTTATGACGCGAAAATATTACCTGTCCAAAATGTAGC**

**hv mxe D 121F 06**  **ACATCATGAACCTCAACGTCACGTTTTTGGTGAACAGCGCCGCTCACATATGGGGCTACAAGCCTTATGACGCGAAAATATTACCTGTCCAAAATGTAGC**

**hv mxe D 138F 06**  **ACATCATGAACCTCAACGTCACGTTTTTGGTGAACAGCGCCGCTCACATATGGGGCTACAAGCCTTATGACGCGAAAATATTACCTGCCCAAAACGTAGC**

**hv mxe E6**  **ACATCATGAACCTCAACGTCACGTTTTTGGTGAACAGCGCCGCTCACATATGGGGCTACAAGCCTTATGACGCGAAAATATTACCTGyCCAAAAyGTAGC**

**hv mxe E7 06**  **ACATCATGAACCTCAACGTCACGTTTTTGGTGAACAGCGCCGCTCACATATGGGGCTACAAGCCTTATGACGCGAAAATATTACCTGTCCAAAATGTAGC**

**hv nc E140F 06**  **ACATCATGAACCTCAACGTCACGTTTTTrGTGAACAGCGCCGCTCACATATGGGGyTACAAGCCTTATGACGCGAAAATATTACCTGCCCAAAAyGTAGC**

**hv nc E142F 06**  **ACATCATGAACCTCAACGTCACGTTyTTGGTGAACAGCGCCGCTCACATATGGGGyTACAAGCCTTATGACGCGAAAATATTACCTGCCCAAAACGTAGC**

**hv nc E 135F**  **ACATCATGAACCTCAACGTCACGTTTTTrGTGAACAGCGCCGCTCACATATGGGGyTACAAGCCTTATGACGCGAAAATATTACCTGCCCAAAAyGTAGC**

**hv nc F4**  **ACATCATGAACCTCAACGTCACGTTyTTGGTGAACAGCGCCGCTCACATATGGGGyTACAAGCCTTATGACGCGAAAATATTACCTGCCCAAAACGTAGC**

**hv nc m07-28**  **ACATCATGAACCTCAACGTCACGTTTTTGGTGAACAGCGCCGCTCACATATGGGGCTACAAGCCTTATGACGCGAAAATATTACCTGyCCAAAACGTAGC**

**hv tx 06-11a**  **ACATCATGAACCTCAACGTCACGTTTTTGGTrAACAGCGCCGCTCACATATGGGGCTACAAGCCTTATGACGCGAAAATATTACCTGTCCAAAATGTAGC**

**hv tx 06 1A**  **ACATCATGAACCTCAACGTCACGTTTTTrGTGAACAGCGCCGCTCACATATGGGGyTACAAGCCTTATGACGCGAAAATATTACCTGCCCAAAATGTAGC**

**hv tx 2 07 16**  **ACATCATGAACCTCAACGTCACGTTTTTGGTGAACAGCGCCGCTCACATATGGGGCTACAAGCCTTATGACGCGAAAATATTACCTGTCCAAAACGTAGC**

**hv tx 2 07 22**  **ACATCATGAACCTCAACGTCACGTTyTTGGTGAACAGCGCCGCTCACATATGGGGyTACAAGCCTTATGAyGCGAAAATATTACCTGyCCAAAAyGTAGC**

**hv tx 2 07 25**  **ACATCATGAACCTCAACGTCACGTTTTTrGTGAACAGCGCCGCTCACATATGGGGyTACAAGCCTTATGACGCGAAAATATTACCTGyCCAAAAyGTAGC**

**hv tx 2 07 27**  **ACATCATGAACCTCAACGTCACGTTTTTGGTGAACAGCGCCGCTCACATATGGGGTTACAAGCCTTAyGACGCGAAAATATTACCTGCCCAAAAyGTAGC**

**hv tx 2 07 28**  **ACATCATGAACCTCAACrTCACGTTTTTGGTGAACAGCGCCGCTCACATATGGGGCTACAAGCCTTATGACGCGAAAATATTACCTGyCCAAAAyGTAGC**

**hv tx 2 07 6**  **ACATCATGAACCTCAACGTCACGTTTTTrGTGAACAGCGCCGCTCACATATGGGGyTACAAGCCTTATGACGCGAAAATATTACCTGyCCAAAAyGTAGC**

**hv tx 2 07 9**  **ACATCATGAACCTCAACGTCACGTTTTTrGTGAACAGCGCCGCTCACATATGGGGCTACAAGCCTTATGACGCGAAAATATTACCTGCCCAAAAyGTAGC**

1610 1620 1630 1640 1650 1660 1670 1680 1690 1700

....|....|....|....|....|....|....|....|....|....|....|....|....|....|....|....|....|....|....|....|

L allel Amino Acid **.V..S..V..A..T..G..G..E..G..F..H..N..Y..H..H..V..F..P..W..D..Y..R..A..A..E..L..G..N..N..S..L..N..L..**

**L allel gDNA**  **TGTGTCCGTCGCGACTGGTGGAGAAGGTTTTCATAATTATCACCACGTGTTCCCTTGGGATTATCGAGCAGCGGAACTCGGGAACAATAGCCTCAATCTG**

**L allel cDNA**  **TGTGTCCGTCGCGACTGGTGGAGAAGGTTTTCATAATTATCACCACGTGTTCCCTTGGGATTATCGAGCAGCGGAACTCGGGAACAATAGCCTCAATCTG**

**H allel fam35-07 gDNA** **TGTGTCCGTCGCGACTGGTGGAGAAGGTTTTCATAATTATCACCACGTGTTCCCTTGGGATTATCGAGCAGCGGAACTTGGGAACAATAGCCTCAATCTG**

**hv ms 07 11**  **TGTGTCAGTCGCGACTGGTGGAGAAGGTTTTCATAATTATCACCACGTGTTCCCTTGGGATTATCGAGCAGCGGAACTTGGGAACAATAGCCTCAATCTG**

**hv ms 07 13**  **TGTGTCCGTCGCGACTGGTGGAGAAGGTTTTCATAATTATCACCACGTGTTCCCTTGGGATTATCGAGCAGCGGAACTTGGGAACAATAGCCTCAATyTG**

**hv ms 07 18**  **TGTGTCCGTCGCGACTGGTGGAGAAGGTTTTCATAATTATCACCACGTGTTCCCTTGGGATTATCGAGCAGCGGAACTTGGGAACAATAGCCTCAATCTG**

**hv ms 07 3**  **TGTGTCmGTCGCGACTGGTGGAGAAGGTTTTCATAATTATCACCACGTGTTCCCTTGGGATTATCGAGCAGCGGArCTyGGGAACAATArCCTCAATCTG**

**hv ms 08 G4**  **TGTGTCmGTCGCGACTGGTGGAGAAGGTTTTCATAATTATCACCACGTGTTCCCTTGGGATTATCGAGCAGCGGAACTCGGkAACAATAGCyTCAATCTG**

**hv ms 08 G 18**  **TGTGTCCGTCGCGACTGGTGGAGAAGGTTTTCATAATTATCACCACGTGTTCCCTTGGGATTATCGAGCAGCGGAACTTGGGAACAATAGCCTCAATyTG**

**hv ms 08 v1**  **TGTGTCAGTCGCGACTGGTGGAGAAGGTTTTCATAATTATCACCACGTGTTCCCTTGGGATTATCGAGCAGCGGAACTTGGGAACAATAGCCTCAATyTG**

**hv ms 08 vl4**  **TGTGTCmGTCGCGACTGGTGGAGAAGGTTTTCATAATTATCACCACGTGTTCCCTTGGGATTATCGAGCAGCGGArCTTGGGAACAATAGCCTCAATCTG**

**hv ms 08 vl 18**  **TGTGTCmGTCGCGACTGGTGGAGAAGGTTTTCATAATTATCACCACGTGTTCCCTTGGGATTATCGAGCAGCGGAACTyGGGAACAATAGCCTCAATCTG**

**hv ms 14**  **TGTGTCmGTyGCGACTGGTGGAGAAGGTTTTCATAATTATCACCACGTGTTCCCTTGGGATTATCGAGCAGCGGAACTyGGGAACAATArCCTCAATCTG**

**hv ms 5**  **TGTGTCCGTCGCGACTGGTGGAGAAGGTTTTCATAATTATCAyCACGTGTTCCCTTGGGATTATCGAGCAGCGGAACTyGGGAACAATAGCCTCAATCTG**

**hv ms vl 12 08**  **TGTGTCAGTCGCGACTGGTGGAGAAGGTTTTCATAATTATCACCACGTGTTCCCTTGGGATTATCGAGCAGCGGAACTTGGGAACAATAGCCTCAATCTG**

**hv mxe 06 10**  **TGTGTCmGTCGCGACTGGTGGAGAAGGTTTTCATAATTATCACCACGTGTTCCCTTGGGATTATCGAGCAGCGGArCTTGGGAACAATAGCCTCAATyTG**

**hv mxe 06 11**  **TGTGTCCGTCGCGACTGGTGGAGAAGGTTTTCATAATTATCAyCACGTGTTCCCTTGGGATTATCGAGCAGCGGAACTyGGGAACAATAGCCTCAATCTG**

**hv mxe D 121F 06**  **TGTGTCmGTCGCGACTGGTGGAGAAGGTTTTCATAATTATCACCACGTGTTCCCTTGGGATTATCGAGCAGCGGAACTTGGGAACAATAGCCTCAATyTG**

**hv mxe D 138F 06**  **TGTGTCCGTCGCGACTGGTGGAGAAGGTTTTCATAATTATCACCACGTGTTCCCTTGGGATTATCGAGCAGCGGArCTTGGGAACAATAGCCTCAATCTG**

**hv mxe E6**  **TGTGTCmGTCGCGACTGGTGGAGAAGGTTTTCATAATTATCACCACGTGTTCCCTTGGGATTATCGAGCAGCGGArCTTGGGAACAATAGCCTCAATyTG**

**hv mxe E7 06**  **TGTGTCAGTCGCGACTGGTGGAGAAGGTTTTCATAATTATCACCACGTGTTCCCTTGGGATTATCGAGCAGCGGAACTTGGGAACAATAGCCTCAATCTG**

**hv nc E140F 06**  **yGTGTCmGTCGCGwCTGGTGGAGAAGGTTTTCATAATTATCACCACGTGTTCCCTTGGGATTATCGAGCAGCGGArCTTGGGAACAATAGCCTCAATCTG**

**hv nc E142F 06**  **TGTGTCCGTCGCGACTGGTGGAGAAGGTTTTCATAATTATCACCACGTGTTCCCTTGGGATTATCGAGCAGCGGAAyTyGGGAACAATAGCCTCAATCTG**

**hv nc E 135F**  **yGTGTCmGTCGCGwCTGGTGGAGAAGGTTTTCATAATTATCACCACGTGTTCCCTTGGGATTATCGAGCAGCGGArCTTGGGAACAATAGCCTCAATCTG**

**hv nc F4**  **TGTGTCCGTCGCGACTGGTGGAGAAGGTTTTCATAATTATCACCACGTGTTCCCTTGGGATTATCGAGCAGCGGAACTyGGGAACAATAGCCTCAATyTG**

**hv nc m07-28**  **TGTGTCCGTCGCGACTGGTGGAGAAGGTTTTCATAATTATCACCACGTGTTCCCTTGGGATTATCGAGCAGCGGAACTyGGGAACAATAGCCTCAATCTG**

**hv tx 06-11a**  **TGTGTCAGTCGCGACTGGTGGAGAAGGTTTTCATAATTATCACCACGTGTTCCCTTGGGATTATCGAGCAGCGGAACTTGGGAACAATAGCCTCAATCTG**

**hv tx 06 1A**  **TGTGTCCGTCGCGACTGGTGGAGAAGGTTTTCATAATTATCACCACGTGTTCCCyTGGGATTATCGAGCAGCGGAACTyGGGAACAATAGCCTCAATCTG**

**hv tx 2 07 16**  **TGTGTCCGTCGCGACTGGTGGAGAAGGTTTTCATAATTATCACCACGTGTTCCCTTGGGATTATCGAGCAGCGGAACTTGGGAACAATAGCCTCAATCTG**

**hv tx 2 07 22**  **TGTGTCCGTCGCGACTGGTGGAGAAGGTTTTCATAATTATCACCACGTGTTCCCTTGGGATTATCGAGCAGCGGArCTyGGGAACAATAGCCTCAATyTG**

**hv tx 2 07 25**  **TGTGTCmGTCGCGACTGGTGGAGAAGGTTTTCATAATTATCACCACGTGTTyCCTTGGGATTATCGAGCAGCGGAACTyGGGAACAATAGCCTCAATCTG**

**hv tx 2 07 27**  **TGTGTCmGTyGCGACTGGTGGAGAAGGTTTTCATAATTATCACCACGTGTTCCCTTGGGATTATCGAGCAGCGGAACTTGGGAACAATAGCCTCAATCTG**

**hv tx 2 07 28**  **TGTGTCmGTCGCGACTGGTGGAGAAGGTTTTCATAATTATCACCACGTGTTCCCTTGGGATTATCGAGCAGCGGAACTyGGGAACAATAGCCTCAATCTG**

**hv tx 2 07 6**  **TGTGTCmGTCGCGACTGGTGGAGAAGGTTTTCATAATTATCACCACGTGTTyCCTTGGGATTATCGAGCAGCGGAACTyGGGAACAATAGCCTCAATCTG**

**hv tx 2 07 9**  **TGTGTCCGTCGCGACTGGTGGAGAAGGTTTTCATAATTATCACCACGTGTTCCCTTGGGATTATCGAGCAGCGGAACTyGGGAACAATArCCTCAATCTG**

1710 1720 1730 1740 1750 1760 1770 1780 1790 1800

....|....|....|....|....|....|....|....|....|....|....|....|....|....|....|....|....|....|....|....|

L allel Amino Acid **T..T..K..F..I..D..F..F..A..W..I..G..W..A..Y..D..L..K..T..V..S..E..D..M..I..K..L..R..T..K..R..T..G..D**

**L allel gDNA**  **ACGACTAAATTCATAGATTTCTTCGCATGGATCGGATGGGCATATGACCTGAAGACGGTTTCGGAAGATATGATAAAACTAAGGACTAAACGCACTGGAG**

**L allel cDNA**  **ACGACTAAATTCATAGATTTCTTCGCATGGATCGGATGGGCATATGACCTGAAGACGGTTTCGGAAGATATGATAAAACTAAGGACTAAACGCACTGGAG**

**H allel fam35-07 gDNA** **ACGACTAAATTCATAGATTTCTTCGCATGGATCGGATGGGCATATGACCTGAAGACGGTTTCGGAAGATATGATAAAATTAAGGACTAAACGCACTGGAG**

**hv ms 07 11**  **ACGACTAAATTCATAGATTTCTTCGCAwGGATCGGATGGGCATATGACCTGAAGACGGTTTCGGAAGATATGATAAAACTAAGGACTAAACGCACTGGAG**

**hv ms 07 13**  **ACGACTAAATTCATAGATTTCTTCGCATGGATCGGATGGGCwTATGACCTGAAGACGGTTTCGGAAGAkATGATAAAACTAAGGAyTAAACGCACTGGAG**

**hv ms 07 18**  **ACGACTAArTTCATAGATTTCTTCGCATGGATCGGATGGGCATATGACCTGAAGACGGTTTCGGAAGATATGATAAAACTyAGGAyTAAACGCACTGGAG**

**hv ms 07 3**  **ACGACTAAATTCATAGATTTCTTCGCATGGATCGGATGGGCATATGACCTGAAGACGGTTTCGGAAGATATGATAAAACTAAGGAyTAAACGCACTGGAG**

**hv ms 08 G4**  **ACGACTAAATTCATAGATTTCTTCGCATGGATCGGATGGGCATATGACCTGAAGACGGTTTCGGAAGATATGATAAAACTAAGGAyTAAACGyACTGGAG**

**hv ms 08 G 18**  **ACGACTAAATTCATAGATTTCTTCGCATGGATCGGATGGGCwTATGACCTGAAGACGGTTTCGGAAGATATGATAAAACTAAGGACTAAACGCACTGGAG**

**hv ms 08 v1**  **ACGACTAAATTCATAGATTTCTTCrCATGGATCGGATGGGCATATGACCTGAAGACGGTTTCGGAAGATATGATAAAACTAAGGACTAAACGCACTGGAG**

**hv ms 08 vl4**  **ACGACTAAATTCATAGATTTCTTCGCATGGATCGGATGGGCATATGACCTGAAGACGGTTTCGGAAGATATGATAAAACTAAGGACTAAACGCACTGGAG**

**hv ms 08 vl 18**  **ACGACTAAATTCATAGATTTCTTCGCATGGATCGGATGGGCATATGACCTGAAGACGGTTTCGGAAGATATGATAAAACTAAGGAyTAAACGCACTGGAG**

**hv ms 14**  **ACGACTAAATTCATAGATTTCTTCGCATGGATCGGATGGGCATATGACCTGAAGACGGTTTCkGAAGATATGATAAAACTAAGGAyTAAACGCACTGGAG**

**hv ms 5**  **ACGACTAAATTCATAGATTTCTTCGCATGGATCGGATGGGCATATGACCTGAAGACGGTTTCGGAAGATATgATAAAACTAAGGAyTAAACGCACTGGAG**

**hv ms vl 12 08**  **ACGACTAAATTCATAGATTTCTTCGCATGGATCGGATGGGCATATGACCTGAAGACGGTTTCGGAAGATATGATAAAACTAAGGACTAAACGCACTGGAG**

**hv mxe 06 10**  **ACGACTAAATTCATAGATTTCTTCGCATGGATCGGATGGGCATATGACCTGAAGACGGTTTCGGAAGAkATGATwAAACTAAGGAyTAAACGCACTGGAG**

**hv mxe 06 11**  **ACGACTAAATTCATAGATTTCTTCGCATGGATCGGATGGGCATATGACCTGAAGACGGTTTCGGAAGATATGATAAAACTAAGGAyTAAACGCACTGGAG**

**hv mxe D 121F 06**  **ACGACTAAATTCATAGATTTCTTCGCATGGATCGGATGGGCATATGACCTGAAGACGGTTTCGGAAGATATGATAAAACTAAGGAyTAAACGCACTGGAG**

**hv mxe D 138F 06**  **ACGACTAArTTCATAGATTTCTTCGCATGGATCGGATGGGCATATGACCTGAAGACGGTTTCGGAAGATATGATAAAACTAAGGACTAAACGCACTGGAG**

**hv mxe E6**  **ACGACTAAATTCATAGATTTCTTCGCATGGATCGGATGGGCATATGACCTGAAGACGGTTTCGGAAGATATGATAAAACTAAGGACTAAACGCACTGGAG**

**hv mxe E7 06**  **ACGACTAAATTCATAGATTTCTTCGCATGGATCGGATGGGCATATGACCTGAAGACGGTTTCGGAAGATATGATAAAACTAAGGATTAAACGCACTGGAG**

**hv nc E140F 06**  **ACGACkAAATTCATAGATTTCTTCGCATGGATCGGATGGGCATATGACCTGAAGACGGTTTCGGAAGATATGATAAAACTAAGGACTAAACGCACTGGAG**

**hv nc E142F 06**  **ACGACTAAATTCATAGATTTCTTCGCATGGATCGGATGGGCATATGACCTGAAGACGGTtTCGGAAGATATGATAAAACTAAGGAyTAAACGCACTGGAG**

**hv nc E 135F**  **ACGACkAAATTCATAGATTTCTTCGCATGGATCGGATGGGCATATGACCTGAAGACGGTTTCGGAAGATATGATAAAACTAAGGACTAAACGCACTGGAG**

**hv nc F4**  **ACGACTAAATTCATAGATTTCTTCGCATGGATCGGATGGGCATATGACCTGAAGACGGTTTCGGAAGATATGATwAAACTAAGGATTAAACGCACTGGAG**

**hv nc m07-28**  **ACGACTAArTTCATAGATTTCTTCGCATGGATCGGATGGGCATATGACCTGAAGACGGTTTCGGAAGATATGATAAAACTAAGGACTAAACGCACTGGAG**

**hv tx 06-11a**  **ACGACTAAATTCATAGATTTCTTCGCATGGATCGGATGGGCATATGACCTGAAGACGGTTTCGGAAGATATGATAAAACTAAGGAyTAAACGCACTGGAG**

**hv tx 06 1A**  **ACGACTAAATTCATAGATTTCTTCGCATGGATCGGATGGGCATATGACCTGAArACGGTTTCGGAAGATATGATAAAACTAAGGACTAAACGCACTGGAG**

**hv tx 2 07 16**  **ACGACTAAATTCATAGATTTCTTCGCATGGATCGGATGGGCATATGACCTGAAGACGGTTTCGGAAGATATGATAAAACTAAGGACTAAACGCACTGGAG**

**hv tx 2 07 22**  **ACGACTAAATTCATAGATTTCTTCGCATGGATCGGATGGGCATATGACCTGAAGACGGTTTCGGAAGATATgATAAAACTAAGGAyTAAACGCACTGGAG**

**hv tx 2 07 25**  **ACGACTAAATTCATAGATTTCTTCGCATGGATCGGATGGGCATATGACCTGAAGACGGTTTCGGAAGAkATGATAAAACTAAGGATTAAACGCACTGGAG**

**hv tx 2 07 27**  **ACGACTAAATTCATAGATTTCTTCGCATGGATCGGATGGGCATATGACCTGAAGACGGTTTCGGAAGATATGATAAAACTAAGGAyTAAACGCACTGGAG**

**hv tx 2 07 28**  **ACGACTAAATTCATAGATTTCTTCGCATGGATCGGATGGGCATATGACCTGAAGACGGTTTCGGAAGATATGATAAAACTAAGGACTAAACGCACTGGAG**

**hv tx 2 07 6**  **ACGACTAAATTCATAGATTTCTTCGCATGGATCGGATGGGCATATGACCTGAAGACGGTTTCGGAAGAkATGATAAAACTAAGGATTAAACGCACTGGAG**

**hv tx 2 07 9**  **ACGACkAAATTCATAGATTTmTTCGCATGGATCGGATGGGCATATGACCTGAAGACGGTTTCGGAAGATAyGATAAAACTAAGGAyTAAACGCACTGGAG**

1810 1820 1830 1840 1850 1860 1870 1880 1890 1900

....|....|....|....|....|....|....|....|....|....|....|....|....|....|....|....|....|....|....|....|

L allel amino acid **..G..T..D..L..W..G..H..E..Q..K..Y..D..E..V..L..D..V..K..D..K..*..**

**L allel gDNA**  **ATGGCACGGATCTTTGGGGACACGAACAAAAATATGATGAAGTATTGGATGTAAAAGATAAATAAAGTTGAATGATGG-TAGAGGTTGCAACAGTGATTT**

**L allel cDNA**  **ATGGCACGGATCTTTGGGGACACGAACAAAAATATGATGAAGTATTGGATGTAAAAGATAAATAAAGTTGAATGATGG-TAGAGGTTGCAACAGTGATTT**

**H allel fam35-07 gDNA** **ATGGCACGGATCTTTGGGGACACGAACAAAAATATGATGAAGTATTGGATGTAAAAGATAAATAAAGTAGAATGATGG-TAGAGGTTCTAACAGTGATTT**

**hv ms 07 11**  **ATGGCACrGATCTTTGGGGACACGAACAAAAATATGATGAAGTATTGGATGyAAAAGATAAATAAAGTwGAATGATGG-TAGAGGTTCTAACAsTGATTT**

**hv ms 07 13**  **ATGGyACsGATCTTTGGGGACACGAACAAAAATATGATGAArTATTGGATGTAAAAGATAArTAAAGTTGAATGATGG-TAGAGGwTCTAACAGTGATTT**

**hv ms 07 18**  **ATGGCACGGATCTTTGGGGACACGAACAAAAATATGATGAAGTATTGGATGTAAAAGATAAATAAAGTTGrATGATGrawAGAGGTTsTAACAGTGATTT**

**hv ms 07 3**  **ATGGCACGGATCTTTGGGGACACGAACAAAAATATGATGAAGTATTGGATGTAAAAGATAAATAAAGTwGAATGATGG-TAGAGGTTCTAACAGTGATTT**

**hv ms 08 G4**  **AyGGCACGGATCTTTGGGGACACGAACAAAAATATGATGAAGTATTGGATGTAAAAGATAAATAAAGTAGrATGATGG-TAGAGGTTCTAACAGTGATTw**

**hv ms 08 G 18**  **ATGGCACGGATCTTTGGGGACACGAACAAAAATATGATGAAGTATTGGATGTAAAAGATAAATAAAGTTGAATGATGG-TAGAGGTTCTAACAGTGATTT**

**hv ms 08 v1**  **ATGGCACGGATCTTTGGGGACACGAACAAAAATATGATGAAGTATTGGATGTAAAAGATAAATAAAGTwkAATGATGrawAGAGGTTsTAACAGTGATTT**

**hv ms 08 vl4**  **ATGGCACrGATCTTTGGGGACACGAACAAAAATATGATGAAGTATTGrATGTAAAAGATAAATAAAGTAGrATGATGG-TAGAGGTTCTAACAGTGATTT**

**hv ms 08 vl 18**  **ATGGCACGGATCTTTGGGGACACGAACAAAAATATGATGAAGTATTGGATGTAAAAGATAAATAAAGTwGAATGATGG-TAGAGGTTCTAACAGTGATTT**

**hv ms 14**  **ATGGCACGGATCTTTGGGGACACGAACAAAAATATGATGAAGTAyTGGATGTAAAAGATAAATAAAGTAGAATGATGG-TAGAGGTTCTAACAGTGATTT**

**hv ms 5**  **ATGGyACsGATCTTTGGGGACACGAACAAAAATATGATGAAGTATTGGATGTAAAAGATAAATAAAGTTkAATGATGrawAGAGGTTsTAACAGTGATTT**

**hv ms vl 12 08**  **ATGGCACGGATCTTTGGGGAyACGAACAAAAATATGATGAAGTATTGGATGTAAAAGATAAATAAAGTwGAATkATGG-TAGArGTTsTAAyAGTGATTT**

**hv mxe 06 10**  **ATGGCACGGATCTTTGGGGACACGAACAAAAATATGATGAAGTATTGGATGTAAAAGATAAATAAAGTwGAATGATGG-TAGAGGTTCTAACAGTGATTT**

**hv mxe 06 11**  **ATGGyACCGATCTTTGGGGACACGAACAAAAATATGATGAAGTATTGGATGTAAAAGATAAATAAAGTwGAATGATGrawAGAGGTTsTAACAGTGATTT**

**hv mxe D 121F 06**  **ATGGyACsGATCTTTGGGGACACGAACAAAAATATGATGAAGTATTGGATGTAAAAGATAAATAAAGTTGAATGATGG-TAGAGGTTCTAACAGTGATTT**

**hv mxe D 138F 06**  **ATGGCACGGATCTTTGGGGACACGAACAAAAATATGATGAAGTATTGGATGTAAAAGATAAATAAAGTTGAATGATGG-TAGAGGTTCTAACAGTGATTT**

**hv mxe E6**  **ATGGCACGGATCTTTGGGGACACGAACAAAAATATGATGAAGTAyTGGATGTAAAAGATAAATAAAGTwGAATGATGG-TAGAGGTTsyAACAGTGATyT**

**hv mxe E7 06**  **ATGGCACGGATCTTTGGGGACACGAACAAAAATATGATGAAGTATTGGATGTAAAAGATAAATAAAGTTGAATGATGG-TAGAGGTTCTAACAGTGATTT**

**hv nc E140F 06**  **ATGGCACGGATCTTTGGGGACACGAACAAAAATATGATGAAGTATTGGATGTAAAAGATAAATAAAGTwGAATGATGG-TAGAGGTTCTAACAGTGATTT**

**hv nc E142F 06**  **ATGGCACGGATCTTTGGGGACACGAACAAAAATATGATGAAGTATTGGATGTAAAAGATAAATAAAGywGAATkATGG-TAGAGGTTsTAAyAGTGATTT**

**hv nc E 135F**  **ATGGCACGGATCTTTGGGGACACGAACAAAAATATGATGAAGTATTGGATGTAAAAGATAAATAAAGTwGAATGATGG-TAGAGGTTCTAACAGTGATTT**

**hv nc F4**  **ATGGTACCGATCTTTGGGGACACGAACAAAAAkATGATGAAGTATTGGATGTAAAAGATAAATAAAGTwGAATGATGG-TAGAGGTTCTAACAGTGATTT**

**hv nc m07-28**  **ATGGCACGGATCTTTGGGGACACGAACAAAAATATGATGAAGTATTGGATGTAAAAGATAAATAAAGTTGAATGATGG-TAGAGGTTCTAACAGTGATTT**

**hv tx 06-11a**  **ATGGCACrGATCTTTGGGGACACGAACAAAAATATGATGAAGTATTGGATGTAAAAGATAAATAAAGTAGAATGATGG-TAGAGGTTsyAACAGTGATTT**

**hv tx 06 1A**  **ATGGCACGGATCTTTGGGGACACGAACAAAAATATGATGAAGTATTGGATGTAAAAGATAAATAAAGTTkAATGATGrawAGAGGTTsTAACAGTGATTT**

**hv tx 2 07 16**  **ATGGCACGGATCTTTGGGGACACGAACAAAAATATGATGAAGTATTGGATGTAAAAGATAAATAAAGTTGAATGATGG-TAGAGGTTCTAACAGTGATTT**

**hv tx 2 07 22**  **ATGGCACGGATCTTTGGGGACACGAACAAAAATATGATGAAGTATTGGATGTAAAAGATAAATAAAGyTGAATkATGG-TAGArGTTsTAAyAGTGATTT**

**hv tx 2 07 25**  **ATGGyACsGATCTTTGGGGACACGAACAAAAATATGATGAAGTAyTGGATGTAAAAGATAAATAAAGTwGAATGATGG-TAGAGGTTsyAACAGTGATTT**

**hv tx 2 07 27**  **ATGGCACGGATCTTTGGGGACACGAACAAAAATATGATGAAGTATTGGATGTAAAAGATAAATAAAGTTGAATGATGrawAGAGGTTGyAACAGTGATTT**

**hv tx 2 07 28**  **ATGGCACGGATCTTTGGGGACACGAACAAAAATATGATGAAGTATTGGATGTAAAAGATAAATAAAGTwGAATGATGG-TAGAGGTTsyAACAGTGATTT**

**hv tx 2 07 6**  **ATGGyACsGATCTTTGGGGACACrAACAAAAATATGATGAAGTAyTGGATGTAAAAGATAAATAAAGTwGAATGATGG-TAGAGGTTsyAACAGTGATTT**

**hv tx 2 07 9**  **ATGGCACGGATCTTTGGGGACACGAACAAAAATATGATGAAGTATTGGATGTAAAAGATAAATAAAGyTkAATkATGrawnnnnnnnnnnnnnnnnnnnn**

1910 1920 1930 1940 1950

....|....|....|....|....|....|....|....|....|....|....|.

L allel Amino Acid

**L allel gDNA**  **ATTAATTGTGATACATATTGTATTTGTATTTATTGTAGTTGCCAGTATGGTTCTGT**

**L allel cDNA**  **ATTAATTGTGATACATATTGTATTTGTATTTATTGTAGTTGCCAGTATGGTTCTGT**

**H allel fam35-07 gDNA** **ATTAATTGTGATACATATTATATTTGTATTTCTTGTAGTTGCCAGTATGGTTCTGT**

**hv ms 07 11**  **ATTArTTGTGATACATATTATATTTGTATTTCTTGTAGTTGCsAGTATGGTT**

**hv ms 07 13**  **ATTAATTGTGATACATATTATATTTGTATTTCTTGTAGTTGCCAGTATGGTT**

**hv ms 07 18**  **ATTAATTGTGATACATATTATATTTGTATTTCTTGTAGTTGCCAGTATGGTT**

**hv ms 07 3**  **ATTAATTGTGATACATATTATATTTGTATTTCTTGTAGTTGCCAGTATGGTT**

**hv ms 08 G4**  **ATTAATTGTGATACATATTATATTTGTATTTCTTGTAGTTGCCAGTATGGTT**

**hv ms 08 G 18**  **ATTAATTGTGATACATATTATATTTGTATTTCTTGTAGTTGCCAGTATGGTT**

**hv ms 08 v1**  **ATTAATTGTGATACATATTATATTTGTATTTmTTGTAGTTGyCAGTATGGTT**

**hv ms 08 vl4**  **ATTAATTGTGATACATATTATATTTGTATTTCTTGTAGTTGCCAGTATGGTT**

**hv ms 08 vl 18**  **ATTAATTGTGATACATATTATATTTGTATTTCTTGTAGTTGCCAGTATGGTT**

**hv ms 14**  **ATTAATTGTGATACATATTAyATTTGTATTTmTTGTAGTTGyCAGTATGGTT**

**hv ms 5**  **ATTwATTGTGATACATATTATATTTGTATTTCTTGTAGTTGCCAGTATGGTT**

**hv ms vl 12 08**  **xTTAATTGTGATACATATTATATTTGTATTTCTTGTAGTTGCCAGTATGGTT**

**hv mxe 06 10**  **ATTAATTGTGATACATATTATATTTGTATTTCTTGTAGTTGyCAGTATGGTT**

**hv mxe 06 11**  **ATTAATTGTGATACATATTATATTTGTATTTCTTGTAGTTGyCAGTATGGTT**

**hv mxe D 121F 06**  **ATTxxxxGTGATACATATTATATTTGTATTTCTTGTAGTTGCCAGTATGGTT**

**hv mxe D 138F 06**  **ATTAATTGTGATACATATTATATTTGTATTTCTTGTAGTTGCCAGTATGGTT**

**hv mxe E6**  **ATTAATTrTGATACATATTATATTTGTATTTmTTrTAGTTGCCAGTATGGTT**

**hv mxe E7 06**  **ATTAATTGTGATACATATTATATTTGTATTTCTTGTAGTTGCCAGTATGGTT**

**hv nc E140F 06**  **ATTAATTGTGATACATATTATATTTGTATTTCTTGTAGTTGyCAGTATGGTT**

**hv nc E142F 06**  **xTTAATTGTGATACATATTATATTTGTATTTCTTGTAGTTGCCAGTATGGTT**

**hv nc E 135F**  **ATTAATTGTGATACATATTATATTTGTATTTCTTGTAGTTGyCAGTATGGTT**

**hv nc F4**  **ATTAATTGTGATACATATTATATTTGTATTTCTTGTAGTTGCCAGTATGGTT**

**hv nc m07-28**  **ATTAATTGTGATACATATTATATTTGTATTTCTTGTAGTTGCCAGTATGGTT**

**hv tx 06-11a**  **ATTAATTGTGATACATATTrTATTTGTATTTmTTGTAGTTGCCAsTATGGTT**

**hv tx 06 1A**  **ATTAATTGTGATACATATTATATTTGTATTTCTTGTAGTTGCCAGTATGGTT**

**hv tx 2 07 16**  **ATTAATTGTGATACATATTATATTTGTATTTCTTGTAGTTGCCAGTATGGTT**

**hv tx 2 07 22**  **xTTAATTGTGATACATATTATATTTGTATTTCTTGTAGTTGCCAGTATGGTT**

**hv tx 2 07 25**  **ATTAATTGTGATACATATTrTATTTGTATTTmTTGTAGTTGCCAGTATkGTT**

**hv tx 2 07 27**  **ATTAATTGTGrTACATATTGTATTTGTATTTATTGTAGTTGCCAGTATGGTT**

**hv tx 2 07 28**  **ATTArTTGTGATACATATTrTATTTGTATTTmTTGTAGTTGCCAGTATGGTT**

**hv tx 2 07 6**  **ATTAATTGTGATACATATTrTATTTGTATTTmTTGTAGTTGCCAGTATkGTT**

**hv tx 2 07 9**  **xTTAATTGTGATACATATTATATTTGTATTTATTGTAGTtGCCAGTATGGTT**
