# Supplementary material for: Within-population variability in a moth sex pheromone blend, part 2: selection towards fixation
Source: R Soc Open Sci. 2019 Mar 13;6(3):182050. doi: 10.1098/rsos.182050 (PMC6458377; doi:10.1098/rsos.182050)
Supplement: Suppl. File 5 [file rsos182050supp6.docx]

Supplementary File 5 to Groot AT, van Wijk M, Villacis-Perez E, Kuperus P, Schöfl G, van Veldhuizen D, Heckel D. Within-population variability in a moth sex pheromone blend, part 2: Selection towards fixation. Royal Society Open Science.

Aligned nucleotide sequences of the second intron after the start codon of LPAQ delta-11-desaturase of field-collected females

1010 1020 1030 1040 1050 1060 1070 1080 1090 1100

....|....|....|....|....|....|....|....|....|....|....|....|....|....|....|....|....|....|....|....|

**L allel gDNA**  **AAAA<GTAAGTATGCTAAACTGATTTTTTTTT----AAGTA------------AATAATAAATA----TTAGATCAACATCAG-----------------**

**L allel cDNA**  **AAAA<-----------------------------------------------------------------------------------------------**

**H allel fam35-07 gDNA** **AAAA<GTAAGT-GTGAAAATA----TTTTTTATTAGA--TATTTTTT-GGA-------------------------------------------------**

**hv nc b202**  **-----GTAAGTACGCTAAACTGATTTTTTTWW----WTGTA------------AATAATAAATA----TTAGATCAACATCAG-----------------**

**hv nc f8 R3.03**  **-----GTAAGTATGCTAAACTGAT-TTTTTTA----AAGTA------------AATAATAAATA----TTAGATCAACATCAG-----------------**

**hv nc ell3f R0.43**  **-----GTAAGTATGCTAAACTGAT-TTTTTTA----AAGTA------------AATAATAAATA----TTAGATCAACATCAG-----------------**

**hv nc f9 R0.38**  **-----GTAAGTATGCTAAACTGAT-TTTTTTA----AAGTA------------AATAATAAATA----TTAGATCAACATCAG-----------------**

**hv nc E-111f R0.51**  **-----GTAAGTATGCTAAACTGAT-TTTTTTA----AAGTA------------AATAATAAATA----TTAGATCAACATCAG-----------------**

**hv nc z 57c R0.27**  **-----GTAAGTATGCTAAACTGAT-TTTTTTA----AAGTA------------AATAATAAATA----TTAGATCAACATCAG-----------------**

**hv tx 06-12a R0.26**  **-----GTAAGTATGCTAAACTGAT-TTTGTAT----AGGCT------------AATAATAAATACTAATTAGATCATCAACGATAAGTATC--ATTTTAA**

**hv nc f1 R0.13**  **-----GTAAGTATGCTAAACTGTT-TTTTTTTTT-TAAGTA------------AATAATAAATA----TTAGATCATCAACGATTGGTATA--ATTTTAA**

**hv nc li co R0.12**  **-----GTAAGTATGCTAAACTGATTTTTTTATAAGTAGGTACCTGTTATAGGTAATAATAAATA----TTAGATCATCAACGATTGGTATATTATTTTAA**

**hv nc p p6 R0.34**  **-----GTAAGTGTGCTAAATA----TTTTTTATTATA--TATTTTTTTGGA-------------------------------------------------**

**hv c 55 lf R0.30**  **-----GTAAGTGTGCAAAATA----TTTTTTATTCGA--TATTTTTTTGGA-------------------------------------------------**

**hv nc 08 28 R0.15**  **-----GTAAGTGTGCAAAATA----TTTTTTATTCGA--TATTTTTTTGGA-------------------------------------------------**

**hv nc b209**  **-----GTAAGTATGCAAAATA----TTTTTTATTCGA--TATTTTTTTGGA-------------------------------------------------**

**hv mxe D-137f R0.44**  **-----GTAAGTGTGCAAAATA----TTTTTTATTCGA--TATTTTTTTGGA-------------------------------------------------**

**hv mxe ao7 R1.01**  **-----GTAAGTGTGCTAAATA----TTTTTTATTCGA--TATTTTTTTGGA-------------------------------------------------**

**hs nc f106f**  **-----GTAAGTAGGTATATTACACAGATTTGATTAGA--TATTTTTTTGGATGCTACTGATTATGAGTGGTACAATTTTAAACTGTTATCTTCTA-----**

**hv mxe a4**  **-----GTAAGTAGGTATATTACACAGATTTGATTAGA--TATTTTTTTGGATGTAACTGATTATGATTTGTACAATTTTAAACTGTTATCTTCTA-----**

**hv mxe a5**  **-----GTAAGTAGGTATATTACACAGATTTGATTAGA--TATTTTTTTGGATGTAACTGATTATGATTTGTACAATTTTAAACTGTTATCTTCTA-----**

1110 1120 1130 1140 1150 1160 1170 1180 1190 1200

....|....|....|....|....|....|....|....|....|....|....|....|....|....|....|....|....|....|....|....|

**L allel gDNA**  **---------------AGGAATTATATACCCAGATTATGATAAAGAACATAAAAAGGATCGGTTTTCCCAATTTTTGATTAGG-AAAGTACTAAATCAAAG**

**L allel cDNA**  **----------------------------------------------------------------------------------------------------**

**H allel fam35-07 gDNA** **----------------------------------------------------------------------------------------------------**

**hv nc b202**  **---------------AGGAATTATATACCATGATTATGATAAAGAACATAAAAAGGATCGGTTTTCCCAATTTTTGATTATGTAAAGTACTAAGTCTTAG**

**hv nc f8 R3.03**  **---------------AGGAATTATATACCAAGATTATGATAAAGAACATAAAAAGGATCGGTTTTCCCAATTTTTGATTAGG-AAAGTACTAAATCAAAG**

**hv nc ell3f R0.43**  **---------------AGGAATTATATACCAAGATTATGATAAAGAACATAAAAAGGATCGGTTTTCCCAATTTTTGATTAGG-AAAGTACTAAATCAAAG**

**hv nc f9 R0.38**  **---------------AGGAATTATATACCAAGATTATGATAAAGAACATAAAAAGGATCGGTTTTCCCAATTTTTGATTAGG-AAAGTACTAAATCAAAG**

**hv nc E-111f R0.51**  **---------------AGGAATTATATACCAAGATTATGATAAAGAACATAAAAAGGATCGGTTTTCCCAATTTTTGATTAGG-AAAGTACTAAATCAAAG**

**hv nc z 57c R0.27**  **---------------AGGAATTATATACCAAGATTATGATAAAGAACATAAAAAGGATCGGTTTTCCCAATTTTTGATTATGTAAAGTATTAAATCAAAG**

**hv tx 06-12a R0.26**  **ATTATAACCAGCGTCGGGAATTATATACACTGGTTATGATAAAGAATATAAAGATGATCGGTTTTCCCAATTTTTGATTATGTAAAGTACTAAATCATAG**

**hv nc f1 R0.13**  **ATTATAACCAGCGTCAGGAATTATAAACACAGATTATGATAAAGAATATAAAGATGATCGGTTTTCTCAATTTTTGATTAGGTAAAGTACTAAATCATAG**

**hv nc li co R0.12**  **ATTATCACCAGCGTCAGAAATTATATACACAG---ATGATAAAGAATGTAAAGATGTTCGGTTTTCCCAATCTTTGATTAGGTAAAGTAATAAATCATAG**

**hv nc p p6 R0.34**  **----------------------------------------------------------------------------------------------------**

**hv c 55 lf R0.30**  **----------------------------------------------------------------------------------------------------**

**hv nc 08 28 R0.15**  **----------------------------------------------------------------------------------------------------**

**hv nc b209**  **----------------------------------------------------------------------------------------------------**

**hv mxe D-137f R0.44**  **----------------------------------------------------------------------------------------------------**

**hv mxe ao7 R1.01**  **----------------------------------------------------------------------------------------------------**

**hs nc f106f**  **----------GCGCCAGGAATGGAAAAATCACCAGGTTATTTTTTAAA---------------TTACCAATTTT--------------------------**

**hv mxe a4**  **----------GCGCCAGGAATGGAAAAATCACCAGGTTATTTTTTAAA---------------TTACCAATTTT--------------------------**

**hv mxe a5**  **----------GCGCCAGGAATGGAAAAATCACCAGGTTATTTTTTAAA---------------TTACCAATTTT--------------------------**

1210 1220 1230 1240 1250 1260 1270 1280 1290 1300

....|....|....|....|....|....|....|....|....|....|....|....|....|....|....|....|....|....|....|....|

**L allel gDNA**  **AAAACTAAACGCCAATTTCACAGGTTCTAGATAA-GTTCTAAATAGATATCTGACAGATCATGTCATAGAAAAGTTATAAAAATTAAGAATATTTCT---**

**L allel cDNA**  **----------------------------------------------------------------------------------------------------**

**H allel fam35-07 gDNA** **----------------------------------------------------------------------------------------------------**

**hv nc b202**  **AAAACTAAACGCCAATTTCACAGGTTCTAGATAA-GTTCTAAATAGATATTTGACAGATCATGTCATACAAAAGCAATAAAAATTAAGAACATTTCT---**

**hv nc f8 R3.03**  **AAAACTAAACGCCAATTTCACAG----------------------------------ATCATATCATAGAAAAGTAATAAAAATTAAGAACATTTCT---**

**hv nc ell3f R0.43**  **AAAACTAAACGCCAATTTCACAG----------------------------------ATCATATCATAGAAAAGTAATAAAAATTAAGAACATTTCT---**

**hv nc f9 R0.38**  **AAAACTAAACGCCAATTTCACAG----------------------------------ATCATATCATAGAAAAGTAATAAAAATTAAGAACATTTCT---**

**hv nc E-111f R0.51**  **AAAACTAAACGCCAATTTCACAG----------------------------------ATCATATCATAGAAAAGTAATAAAAATTAAGAACATTTCT---**

**hv nc z 57c R0.27**  **AAAACTAAACGCCAATTTCACAG-------------------------------------GTTCTATAGATAAGTTCTAAAT----AGATATCTGAC---**

**hv tx 06-12a R0.26**  **AAATTTAAGCGCCAATTTCACAGGTTCTAGATAAAGTTCTAAATAGATATCTGACAGATCATGCCATAGAAGAGTAATAAAAATTAAGAACATTTCT---**

**hv nc f1 R0.13**  **AAAACTAAGCGCCAATTTCACAGGTTCTAGATAAAGTTCTAAATATGTAG----------ATATCTGACAGATGCCATAGAAAAGTAGAATATTTAT---**

**hv nc li co R0.12**  **AATACTAAGCGCCAATTTCACAGGTTCTAGATAAAGTTCTAAATATGTAG----------ATATCTGACCGATGCCATAAAAAAGTAGAATACCTATTTC**

**hv nc p p6 R0.34**  **----------------------------------------------------------------------------------------------------**

**hv c 55 lf R0.30**  **----------------------------------------------------------------------------------------------------**

**hv nc 08 28 R0.15**  **----------------------------------------------------------------------------------------------------**

**hv nc b209**  **----------------------------------------------------------------------------------------------------**

**hv mxe D-137f R0.44**  **----------------------------------------------------------------------------------------------------**

**hv mxe ao7 R1.01**  **----------------------------------------------------------------------------------------------------**

**hs nc f106f**  **----------------------------------------------------------------------------------------------------**

**hv mxe a4**  **----------------------------------------------------------------------------------------------------**

**hv mxe a5**  **----------------------------------------------------------------------------------------------------**

1310 1320 1330 1340 1350 1360 1370 1380 1390 1400

....|....|....|....|....|....|....|....|....|....|....|....|....|....|....|....|....|....|....|....|

**L allel gDNA**  **-ATGGCATCTCCCATAACATGATCTGTCAGATAGCTATCTGGGACTCTACCTGGAACTTGTGAAACTGGCCGTAAATCTTGTTCTAAACTCAATACGCAG**

**L allel cDNA**  **----------------------------------------------------------------------------------------------------**

**H allel fam35-07 gDNA** **-------------------------------------------------------------------------------------------CATCGCAAA**

**hv nc b202**  **-ATGGCATCTCCCATAGCATGATCTGTCAGATAGCTATCTGGGACTCTACCTGGAACTTGTGAAACTGGCCGTAAATCTTATTCTAAACTCAGTACGCAG**

**hv nc f8 R3.03**  **-GTGGCATCTCCCATAGCATGATCTGTCAGATAGCTATCTGGGACTCTACTTGGAACTTGTGAAACTGGCCGTAAATCTTACTCTAATCTCAATACGCAG**

**hv nc ell3f R0.43**  **-GTGGCATCTCCCATAGCATGATCTGTCAGATAGCTATCTGGGACTCTACTTGGAACTTGTGAAACTGGCCGTAAATCTTACTCTAATCTCAATACGCAG**

**hv nc f9 R0.38**  **-GTGGCATCTCCCATAGCATGATCTGTCAGATAGCTATCTGGGACTCTACTTGGAACTTGTGAAACTGGCCGTAAATCTTACTCTAATCTCAATACGCAG**

**hv nc E-111f R0.51**  **-GTGGCATCTCCCATAGCATGATCTGTCAGATAGCTATCTGGGACTCTACTTGGAACTTGTGAAACTGGCCGTAAATCTTACTCTAATCTCAATACGCAG**

**hv nc z 57c R0.27**  **-AGATCATCTCCCATAGCATGATCTGTCAGATAGCTATCTGGGACTCTACTTGCAACTTGTGAAACTGGTCGTAAATCTTACTCTTAACTTAATACGCAG**

**hv tx 06-12a R0.26**  **-ATGGCATCTCCCATAGCATGATCTGTCAGATAGCTATCTGGGACTCTACCTAGAACTTGTGAAACTGGCCGTAAATCTTATTCTAAACTCAATACGCAG**

**hv nc f1 R0.13**  **-ATGGCATCTCCCATAGCATGATCTGTCAGATAGCTATCTGGGACTCTACCTGGAACTTGTGAAACTGGCCGTAAATCTTATTCTAAACTCAATACGCAG**

**hv nc li co R0.12**  **TATGGCATCTCCCATAGCCTGGTCTGTGAGATAGCTATCTGGGACTCTACCTGGAACTTGTGAAACTGGCCGTAAATCTTATTCTAAACTCAATACGCAG**

**hv nc p p6 R0.34**  **-------------------------------------------------------------------------------------------CATCGCAAA**

**hv c 55 lf R0.30**  **-------------------------------------------------------------------------------------------CATCGCAAA**

**hv nc 08 28 R0.15**  **-------------------------------------------------------------------------------------------CATCGCAAA**

**hv nc b209**  **-------------------------------------------------------------------------------------------CATCGCAAA**

**hv mxe D-137f R0.44**  **-------------------------------------------------------------------------------------------CATCGCAAA**

**hv mxe ao7 R1.01**  **-------------------------------------------------------------------------------------------CATCGCAAA**

**hs nc f106f**  **-------------------------------------------------------------------GTGCCAAAATC-------AAAACACACAGCACG**

**hv mxe a4**  **-------------------------------------------------------------------GTGCCAAAATC-------AAAACACACAGCACT**

**hv mxe a5**  **-------------------------------------------------------------------GTGCCAAAATC-------AAAACACACAGCACT**

1410 1420 1430 1440 1450 1460 1470 1480 1490 1500

....|....|....|....|....|....|....|....|....|....|....|....|....|....|....|....|....|....|....|....|

**L allel gDNA**  **AACATAGATATACATAAATTGTGACAAAACTTTCGTTTTTC-TGCAG>ATACGCCGTACCCTTCATTGGAGCTGTTTGTTTCGTCTTACCTACATTAATA**

**L allel cDNA**  **----------------------------------------------->ATACGCCGTACCCTTCATTGGAGCTGTTTGTTTCGTCTTACCTACATTAATA**

**H allel fam35-07 gDNA** **TACATATATATTCAGAAATTGTGTAAAAACCTCCGTTTTCCTTGCAG>GTACGCCATACCCTTCATCGGAGCTGTTTGTTTCGTCTTACCTACATTGATA**

**hv nc b202**  **AACATAGATATACAGAAATTGTGACAAANNNNNNGTTTTTC-TGCAG**

**hv nc f8 R3.03**  **AAAATATATGTACGTAAATTGTGAAAAGACTTTCGTTTTTC-TGCAG**

**hv nc ell3f R0.43**  **AAAATATATGTACGTAAATTGTGAAAAGACTTTCGTTTTTC-TGCAG**

**hv nc f9 R0.38**  **AAAATATATGTACGTAAATTGTGAAAAGACTTTCGTTTTTC-TGCAG**

**hv nc E-111f R0.51**  **AAAATATATGTACGTAAATTGTGAAAAGACTTTCGTTTTTC-TGCAG**

**hv nc z 57c R0.27**  **AACATAGATATACAGAAATTGTGACAAAACTTTCGTTTTTC-TGCAG**

**hv tx 06-12a R0.26**  **GACATAGATATACAGAAATTATGACAAAACTTTCGTTTTTC-TGCAG**

**hv nc f1 R0.13**  **AACATAGATATA--GAAATTGTAAAAAAACCTCCGTTTTCC-TGCAG**

**hv nc li co R0.12**  **AACATAGATATACAGAAATTGTGACAAAACTTTCGTTTTTC-TGCAG**

**hv nc p p6 R0.34**  **TACATATAGATTCAGAAATTGTGTAAAAACCTCCGTTTTCCTTGCAG**

**hv c 55 lf R0.30**  **TACATATAGATTCAGAAATTGTGTAAAAACCTCCGTTTTCCTTGCAG**

**hv nc 08 28 R0.15**  **TACATATAGATTCAGAAATTGTGTAAAAACCTCCGTTTTCCTTGCAG**

**hv nc b209**  **TACATATATATTCAGAAATTGTGTAAAAACCTCCGTTTTCCTTGCAG**

**hv mxe D-137f R0.44**  **TACATATATATTCAGAAATTGTGTAAAAACCTCCGTTTTCCTTGCAG**

**hv mxe ao7 R1.01**  **TACATATATATTCAGAAATTGTGTAAAAACCTCCGTTTTCCTTGCAG**

**hs nc f106f**  **AGG--ATATA--CAGAAATTGGGTAAAAATCTCATCTTTCTTTGCAG**

**hv mxe a4**  **AGG--ATATA--CAGAAATTGGGTAAAAATCTCATCTTTCTTTGCAG**

**hv mxe a5**  **AGG--ATATA--CAGAAATTGGGTAAAAATCTCATCTTTCTTTGCAG**
