# Supplementary material for: Within-population variability in a moth sex pheromone blend, part 2: selection towards fixation
Source: R Soc Open Sci. 2019 Mar 13;6(3):182050. doi: 10.1098/rsos.182050 (PMC6458377; doi:10.1098/rsos.182050)
Supplement: Suppl. File 6 [file rsos182050supp7.docx]

Supplementary File 6 to Groot AT, van Wijk M, Villacis-Perez E, Kuperus P, Schöfl G, van Veldhuizen D, Heckel D. Within-population variability in a moth sex pheromone blend, part 2: Selection towards fixation. Royal Society Open Science.

Gels showing the intron-size polymorphism of the second intron of LPAQ delta-11-desaturase.

=================================================================

Plate 1-4: Field-collected females (see Suppl. File 8 for details on these plates)
